# Incidence, costs and post-operative complications following ankle fracture – A US claims database analysis

**DOI:** 10.1186/s12891-022-06095-x

**Published:** 2022-12-26

**Authors:** Mari F. Vanderkarr, Jill W. Ruppenkamp, Mollie Vanderkarr, Anjani Parikh, Chantal E. Holy, Matthew Putnam

**Affiliations:** 1 Epidemiology & Real-World Data Sciences, Johnson & Johnson MedTech, New Brunswick, NJ USA; 2 DePuy Synthes, Inc. , West Chester, PA USA

**Keywords:** Ankle fractures, Comorbidities, Complications, Costs, Payments, Claims database, Pain, Infection, Reoperation

## Abstract

**Background:**

The epidemiology and payer costs for ankle fractures are not well documented. This study evaluated: (1) the incidence of ankle fracture and ankle surgery following fracture in the US population; and (2) the clinical presentation of patients presenting with ankle fractures requiring surgery, their complication rates, and payer costs.

**Methods:**

Patients in the IBM® MarketScan® Commercial and Medicare Supplemental databases with an inpatient/outpatient diagnosis of ankle fracture from 2016 to 2019 were stratified by age group and gender, and rates of fracture per 10,000 enrollees were estimated. Surgically-treated patients between January 2016 – October 2021 were further analyzed. One-year post-surgical outcomes evaluated complication rates (e.g., infection, residual pain), reoperations, and 1-year payments. Standard descriptive statistics were calculated for all variables and outcomes. Generalized linear models were designed to estimate payments for surgical care and incremental payments associated with postoperative complications.

**Results:**

Fracture cases affected 0.14% of the population; 23.4% of fractures required surgery. Pediatric and elderly patients were at increased risk. From 3 weeks to 12 months following index ankle surgery, 5.5% (5.3% - 5.7%) of commercially insured and 5.9% (5.1% - 6.8%) of Medicare patients required a new surgery. Infection was observed in 4.4% (4.2% - 4.6%) commercially insured and 9.8% (8.8% - 10.9%) Medicare patients, and residual pain 3 months post-surgery was observed in 29.5% (28.7% - 30.3%) commercially-insured and 39.3% (36.0% - 42.6%) Medicare patients. Commercial payments for index surgery ranged from $9,821 (95% CI: $9,697 - $9,945) in the ambulatory surgical center to $28,169 (95% CI: $27,780 - $28,559) in the hospital inpatient setting, and from $16,775 (95% CI: $16,668 - $16,882) in patients with closed fractures, to $41,206 (95% CI: $38,795 - $43,617) in patients with Gustilo III fractures. Incremental commercial payments for pain and infection averaged $5,200 (95% CI: $4,261 - $6,139) and $27,510 (95% CI: $21,759 - $33,261), respectively.

**Conclusion:**

Ankle fracture has a high incidence and complication rate. Residual pain affects more than one-third of all patients. Ankle fracture thus presents a significant societal impact in terms of patient outcomes and payer burden.

**Supplementary Information:**

The online version contains supplementary material available at 10.1186/s12891-022-06095-x.

## Background

Ankle fractures are common, most often due to sports injuries in younger people or falls in the elderly, with an incidence rate of inpatient hospitalization with ankle fracture estimated at 4.22 per 10,000 person-years in the United States [1]. Up to 25% of all ankle fractures require surgical care [2]. A decision to operate is usually based on fracture stability and the patient’s fitness for surgery [3]. Surgical repair of ankle fractures has been shown to be safe and effective, with low rates of 30-day reoperation (1.15%) and emergency room visits (4.39%) [4]. These surgeries have increasingly been performed in low-acuity care settings such as outpatient hospitals or ambulatory surgical centers (ASCs) [5].

The outcomes of ankle fracture surgery are generally favorable, but when postoperative complications occur, they can lead to morbidity, decreased quality of life, and increased health care costs [6]. Surgical site infections (SSI) are one of the most common postoperative complications of ankle fracture surgery. Overall SSI rates reported in the literature vary from 1.4 to 13.0% [7, 8]. Other potential complications of ankle fracture surgery include necrosis of the soft tissue, malunion, nonunion, muscular atrophy, and pain [9, 10]. When surgery is not indicated, the primary complication of non-operative treatments, which includes physical therapy, removable casts, and pharmaceuticals, is the risk of displacement of fragments or widening of the ankle mortise [11]. Predisposing factors for complications following ankle fracture include older age, smoking, diabetes, open fractures, and alcoholism [7]. Among patients with diabetes, severe ankle fractures can lead to Charcot arthropathy with destruction of the bone and possible amputation [12, 13]. Moreover, insulin dependance may increase the risk of 30-day postoperative complications in patients with ankle fracture [14].

Various studies have evaluated ankle fracture complication rates and associated risk factors, as well as costs of ankle fracture surgery [4, 15–17]. Studies published to date represent either a subset of Medicare patients or patients treated specifically in the inpatient setting [18, 19]. A 1987 epidemiological study of ankle fracture was conducted in the US, followed by two more recent ones in Denmark and Sweden [20–22]. To the best of our knowledge, no other recent, US-based, comprehensive analysis of ankle fracture epidemiology, patient characteristics, their clinical journey, costs and outcomes, has been published. Therefore, we conducted a United States-based healthcare claims database analysis to assess: (1) the epidemiology of ankle fracture patients, both identified in the in- and outpatient settings, estimates of surgically vs. non-surgically treated populations; and (2) for surgically-treated patients: the clinical characteristics, treatment types, complication rates, and payments associated with ankle fracture, from the perspective of a US Commercial healthcare payer.

## Methods

### Data source

This retrospective cohort study utilized data from the IBM ® MarketScan® Commercial Claims and Encounters (CCAE) and Medicare Supplemental databases. The CCAE database comprises enrollment information, demographics, and adjudicated inpatient medical, outpatient medical, and outpatient pharmacy claims data collected from over 300 large, self-insured United States (US) employers and over 25 US health plans. From 2000 to 2021, there were 157 million patients included in CCAE. The Commercial database includes information for individuals who are under the age of 65 and are the primary insured or a spouse or dependent thereof. The Medicare Supplemental database contains similar patient-level information as the CCAE database, but for patients with supplemental Medicare coverage, and thus includes patients aged 65 and above, and currently includes approximately 10 million patients.

### Study population

Two distinct patient cohorts were created. To answer the question of ankle fracture occurrence and surgical care epidemiology, all patients from the CCAE and Medicare Supplemental databases were identified if they had one of the following International Classification of Disease (ICD)-10 diagnosis code: fracture of the lower end of the tibia, fracture of the medial or lateral malleolus, or bimalleolar or trimalleolar or Maisonneuve’s or pilon tibial fracture. Only those codes indicative of the initial encounter were included. A complete list of codes used in this study is included in the Supplemental Files. Each calendar year (2016, 2017, 2018 and 2019) was analyzed separately, thus a patient with initial encounters of ankle fractures in 2 distinct and separate calendar years was counted twice, once in each year; however, patients with multiple diagnoses within the same calendar year, or diagnoses for subsequent encounter of the same fracture, were counted for only once. Presence of repair surgery, and the site of the surgery [inpatient, outpatient or office, ambulatory surgical center (ASC), other], were analyzed by identifying patients with Common Procedural Terminology (CPT-4) codes indicative of open surgical treatment for ankle fracture repair, between the date of the fracture diagnosis and up to 15 days post-initial diagnosis. To avoid baseline noise in the data due to cases with very short enrollment periods, only patients with a full 12-months continuous enrollment during the calendar year were included.

To evaluate clinical characteristics, post-operative complication rates, and payments among patients with surgical treatment for ankle fractures, only patients with an actual surgical treatment for ankle fracture repair at time of fracture were further analyzed. The “index” is defined in this study as the date of surgery. Patients were required to have continuous medical enrollment at least three months pre-index to ensure identification of the first surgery, and baseline assessment of comorbidities.

### Variables

The following baseline characteristics were collected from the surgical cohort: patient demographics (i.e., age and sex), comorbidities (i.e., Elixhauser comorbidity index and associated comorbid conditions [23–25]), fracture anatomy and surgical intervention.

### Outcomes

The presence of diagnoses indicative of the following complications was analyzed in the 12 months post-index: infection (deep or SSI), necrosis, deformity, joint derangements, instability, mechanical complications, nonunion, malunion, delayed healing, or refracture. Post-operative arthritis was identified as a diagnosis of ankle arthritis in patients that did not have any ankle arthritis diagnosis before surgery. Pain was defined as a new diagnosis of lower leg pain from 91 days post-index and up to 12 months post index. The period of time from surgery to 90-day post-surgery was excluded in the pain analysis, to avoid including patients with normal, post-operative pain, in the postoperative complication category of continued ankle pain. This threshold was also selected because chronic (vs. acute postsurgical) pain is defined as pain that persists at least 3 months after the surgery [26].

Ankle reoperation, defined as a new open ankle procedure, or closed ankle procedure requiring anesthesia, in the 12-months post-index, was analyzed for all patients, from the day after index surgery. Ankle revision was defined as a reoperation occurring after the first 3 weeks following the index surgery. This distinction between reoperation and revision was made to exclude, in the revision rates, cases that were reoperated after surgery due to imperfect alignment, and were therefore not associated with postoperative complications, but simply underwent a further surgery to perfect the first procedure. These secondary procedures are not uncommon and should really be regarded as part of the initial surgery, not a revision surgery. This is why we excluded them from the actual “revision” counts.

Index (from admission to discharge for inpatient care, or for the entire day of treatment, for outpatient care) and index to 12-month all-cause payments were analyzed for all commercially-insured patients.

### Data analysis

For the epidemiological analysis of ankle fracture and ankle surgical treatment rates, the incidence of fractures and surgeries per 10,000 patient-years were estimated for the entire ankle fracture population, as well as by age- and sex strata. (Age strata: from 0 to 85 and older, by 5-year increment; Each stratum: separate analysis for males and females).

To estimate outcomes and payments following surgery, descriptive statistics were reported for all study variables (i.e., means and standard deviations [SDs] for continuous variables and frequencies and percentages for categorical variables). Generalized linear models were built to estimate payments of complications following surgery. The healthcare payments associated with each complication were estimated using the least means estimates of all-cause payments in patients with vs. without each complication (separately). All payments were adjusted for inflation to 2021 US dollars consumer price index (CPI). For all analyses, a critical value for significance was set at *p* < 0.05. All analyses were performed in R, version 4.0.5.

## Results

### Incidence of ankle fracture and ankle fracture surgery in the United States

**Fig. 1:**
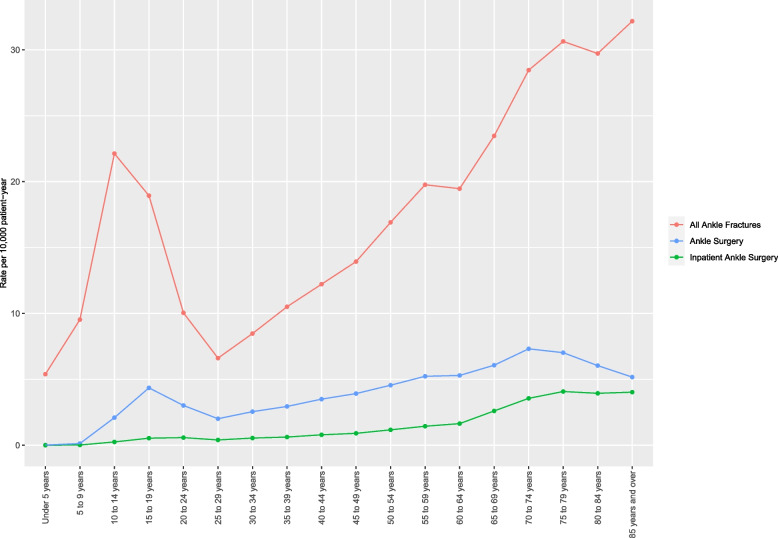
Rate of ankle fracture, ankle surgery (any site) and inpatient-only ankle surgery, per 10,000 person-years, in the United States, from 2016 to 2019, by age group. Ankle fracture cases increase during childhood and peak at age 14. A gradual decrease in fracture cases is then observed until age 29. Fracture cases increase again with age, from age 29 onwards. Rates of surgery follows similar trends, however inpatient cases become more prevalent with increased age

**Fig. 2:**
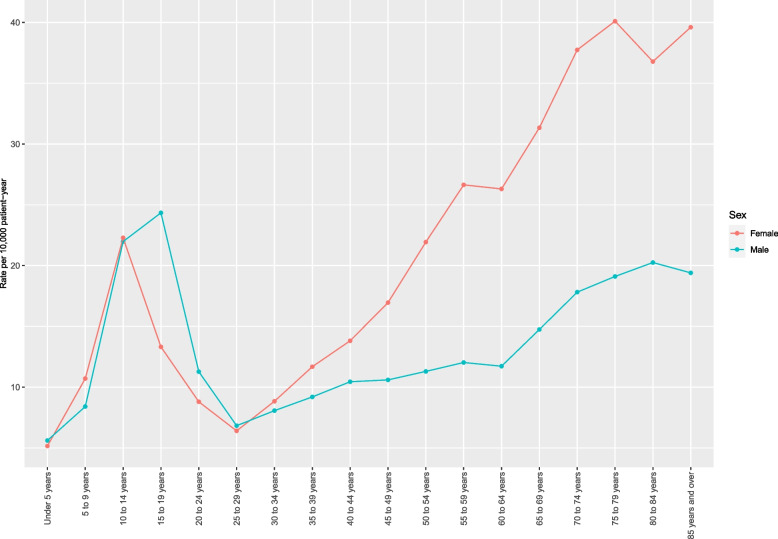
Rate of ankle fracture per 10,000 person-years in the United States, from 2016 to 2019, by sex and age group. In pediatric cases, the rate of ankle fracture is similar between males and females up to age 14, but is greater in males vs. females between ages 15–19. From age 30 onwards, ankle fracture is more prevalent in females vs. males for all age groups

The incidence of ankle fracture in the United States between 2016 and 2019 was 14.1 per 10,000 patient-years. The incidence of surgeries for ankle fracture was 3.3 per 10,000 patient-years (23.4% of all fractures), of which inpatient care was required for 0.8 per 10,000 (24.2% of all surgeries). An analysis by age-group is shown in Fig. 1. Ankle fracture cases increase during childhood and peak at age 14. A gradual decrease in fracture cases is then observed until age 29. Fracture cases increase again with age, from age 29 onwards. Inpatient care increased gradually with age and was mostly performed in older patients. The incidence of ankle fracture by sex is shown in Fig. 2. In the pediatric population, males aged 15–19 had greater incidence of fracture than their female counterparts. For adults 30 and above, incidence of ankle fracture was greater for female vs. males for all age groups. The gender gap increased with increasing age.

**Fig. 3:**
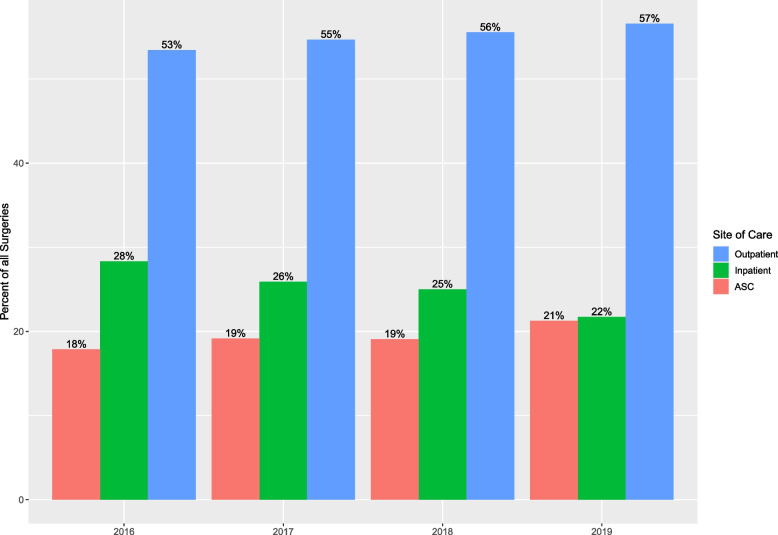
Site of care for ankle fracture surgeries, from 2016 to 2019, based on US nationwide projections. Inpatient cases represented 28% of all ankle fracture surgery cases in 2016 and declined to 22% in 2019. Outpatient and ambulatory surgical center (ASC) cases increased from 53% and 18% of all ankle surgeries in 2016 to 57% and 21% in 2019, respectively

A site of care analysis is shown in Fig. 3. From 2016 to 2019, the percentage of cases treated in the inpatient vs. outpatient and ASC sites changed. A decrease in inpatient cases was observed, while both outpatient and ASC cases have increased during that time frame.

#### Analysis of patients treated surgically for ankle fracture

Patient demographics are shown in Table 1. A total of 50,732 patients were identified, 46,619 from the Commercial database and 4,113 from the Medicare Supplemental database. The difference in population size between Commercial and Medicare is not a reflection of actual prevalence in the US population, it is due to the difference in size of the Commercial and Medicare databases used for this analysis. Because two distinct databases were used, results from commercial and Medicare patients were not pooled but are shown separately. In the commercial cohorts, patients less than 15 represented 5% of the total cohort, with most patients between 30 and 64 years of age. In the Medicare cohort, the majority of patients were between 65 and 74 years of age. In the commercial cohort, 58% of patients were female, whereas in Medicare, 72% were female. Site of care differences were also noticeable: in the commercial cohort, only 21% of patients were treated in the inpatient setting. For Medicare, this percentage increased to 50%.

**Table 1:** Demographic characteristics, year of procedure, and site of care for patients with ankle fracture surgery included in the analysis

**Variable**	**Commercial**	**Medicare**
**All**	46,619	4,113
**Age: mean (SD)**	41.11(15.88)	74.41(7.31)
**Age group**		
** 0 to 14 years**	2,190 (4.7%)	-
** 15 to 29 years**	10,923 (23.4%)	-
** 30 to 64 years**	33,506 (71.9%)	-
** 65 to 74 years**	-	2,375 (57.7%)
** 75 to 84 years**	-	1,267 (30.8%)
** 85 years and over**	-	471 (11.5%)
**Gender: Female**	26,816 (57.5%)	2,945 (71.6%)
**Year of Procedure**		
** 2016**	9,742 (20.9%)	1,326 (32.2%)
** 2017**	8,749 (18.8%)	879 (21.4%)
** 2018**	8,771 (18.8%)	581 (14.1%)
** 2019**	7,398 (15.9%)	403 (9.8%)
** 2020**	6,822 (14.6%)	521 (12.7%)
** 2021 (partial year)**	5,137 (11.0%)	403 (9.8%)
**Site of surgical care**		
** Outpatient**	26,880 (57.7%)	1,741 (42.0%)
** Inpatient**	10,037 (21.5%)	2,072 (50.4%)
** Ambulatory surgery center**	9,702 (20.8%)	300 (7.3%)

*Abbreviation*: *SD *standard deviation

Patient comorbidities are shown in Table 2. The increased Elixhauser index between commercial and Medicare patients (commercial: 0.85 (SD: 1.33); Medicare: 2.69 (SD: 2.3)) reflects increases in age-related comorbidities between these cohorts. Detailed rates for all major comorbid conditions are shown in Table 2.

**Table 2:** Baseline Elixhauser comorbidity score and top 10 comorbidities for patients with ankle fracture surgery

**Variable**	**Commercial**	**Medicare**
**Elixhauser: mean (SD)**	0.85 (1.33)	2.69 (2.3)
**Elixhauser Index**		
** 0**	26,550 (57.0%)	703 (17.1%)
** 1 or 2**	15,250 (32.7%)	1,548 (37.6%)
** 3 or 4**	3,667 (7.9%)	1,049 (25.5%)
** 5 or greater**	1,152 (2.5%)	813 (19.8%)
**Key Comorbidities**		
** All Hypertension**	9,292 (19.9%)	2,677 (65.1%)
* Hypertension with complication*	619 (1.3%)	544 (13.2%)
** Depression**	4,597 (9.9%)	580 (14.1%)
** Obesity**	4,098 (8.8%)	515 (12.5%)
** All Diabetes**	3,497 (7.5%)	1,117 (27.2%)
* Diabetes with complication*	1,794 (3.8%)	701 (17.0%)
** Chronic pulmonary disease**	2,865 (6.2%)	639 (15.5%)
** Hypothyroidism**	2,581 (5.5%)	737 (17.9%)
** Cardiac arrhythmia**	2,233 (4.8%)	900 (21.9%)
** Fluid and electrolyte disorders**	1,602 (3.4%)	522 (12.7%)
** Alcohol Abuse**	1,258 (2.7%)	108 (2.6%)
** Other neurological disorders**	870 (1.9%)	307 (7.5%)
** Rheumatoid Arthritis/ collagen**	782 (1.7%)	208 (5.1%)
** Renal Failure**	528 (1.1%)	514 (12.5%)
** Congestive Heart Failure**	490 (1.1%)	446 (10.8%)

*Abbreviation*: *SD *standard deviation

The presenting diagnoses of patients with ankle fractures are shown in Table 3. The majority of commercial patients (63%) and half of the Medicare patients presented with only one ankle-related fracture diagnosis, the most common being fracture of the lateral malleolus of the fibula, followed by bimalleolar fracture of the lower leg. The remaining 37% of commercial and 50% of Medicare patients presented with more than one fracture diagnosis.

**Table 3:** Ankle fracture diagnoses of patients with ankle fracture, at index

**Variables**	**Commercial**	**Medicare**
**Cohort of Patients with Only 1 Fracture Diagnosis at Time of Surgery**	**29,187 (62.6%)**	**2,070 (50.3%)**
* Fracture of Lateral Malleolus of Fibula*	*8,818 (30.2%)*	*480 (23.2%)*
* Bimalleolar Fracture of Lower Leg*	*5,020 (17.2%)*	*594 (28.7%)*
* Trimalleolar Fracture of Lower Leg*	*4,348 (14.9%)*	*477 (23.0%)*
* Other Fracture of Lower Leg*	*4,174 (14.3%)*	*211 (10.2%)*
* Fracture of Medial Malleolus of Tibia*	*3,079 (10.5%)*	*161 (7.8%)*
* Fracture of Lower End of Tibia*	*2,741 (9.4%)*	*116 (5.6%)*
* Pilon Fracture of Tibia*	*532 (1.8%)*	*16 (0.8%)*
* Maisonneuve’s Fracture of Leg*	*475 (1.6%)*	*15 (0.7%)*
**Cohort of Patients with Multiple Ankle Fracture Diagnoses at Time of Surgery**	**17,432 (37.4%)**	**2,043 (49.7%)**
* 2 Diagnoses*	*12,073 (25.9%)*	*1,295 (31.5%)*
* 3 Diagnoses*	*4,156 (8.9%)*	*574 (14.0%)*
* 4 or more Diagnoses*	*1,203 (2.6%)*	*174 (4.2%)*

One-year postoperative complication, reoperation and revision rates for all patients are shown in Table 4. For all patients, the most common complications were residual pain, followed by joint derangements. All other complications had rates less than 5% in the commercial population. In the Medicare population, re-fracture and infection were common, at 11% and 10%, respectively.

**Table 4:** Complication, reoperation and revision rates. Reoperation includes any new ankle surgery from day after index to 365 days post-index. Revision only includes reoperations of the ankle performed after 21 days, to exclude touch-up cases observed immediately post-surgery and indicative of possible misalignment, versus actual revisions due to post-operative complications

**Outcome**	**Commercially Insured Patients (95%CI)**	**Medicare Supplemental Insured Patients (95%CI)**
**Any reoperation (From Day 1 to Day 365 Post Index)**	10.3% (10.0% − 10.6%)	9.4% (8.4% − 10.5%)
**Revisions (From Day 22 to Day 365 Post Index)**	5.5% (5.3% − 5.7%)	5.9% (5.1% − 6.8%)
**Complication Types**^a^		
Necrosis	0.1% (0.1% − 0.1%)	0.1% (0.0% − 0.2%)
Deformity	0.3% (0.2% − 0.3%)	0.6% (0.4% − 0.9%)
Malunion	0.8% (0.7% − 0.9%)	1.2% (0.9% − 1.6%)
New Arthritis	1.8% (1.7% − 2.0%)	2.5% (1.9% − 3.0%)
Instability	1.9% (1.8% − 2.0%)	2.2% (1.7% − 2.6%)
Nonunion	2.5% (2.3% − 2.7%)	3.6% (2.9% − 4.2%)
Delayed Healing	2.7% (2.5% − 2.9%)	4.5% (3.8% − 5.2%)
Refracture	3.6% (3.4% − 3.8%)	11.4% (10.3% − 12.5%)
Mechanical Complications	3.9% (3.7% − 4.1%)	4.2% (3.5% − 4.9%)
Infection	4.4% (4.2% − 4.6%)	9.8% (8.8% − 10.9%)
Joint Derangements	24.6% (24.1% − 25.1%)	17.3% (15.8% − 18.7%)
Residual Pain > 3 Months Post Surgery	29.5% (28.7% − 30.3%)	39.3% (36.0% − 42.6%)

^a^Patients could present with multiple complication types

Table 5 shows commercial payments for the index ankle fracture surgery, by site of care (inpatient, outpatient or ASC) or fracture severity (open, Gustilo I-II or Gustilo III). Patients treated in the ASC had the average lowest payments, slightly below US$ 10K ($9,821 (95% CI: $9,697 - $9,945)). In contrast, for patients requiring inpatient care, payments averaged $28,169 (95% CI: $27,780 - $28,559). Index payments were also, as expected, strongly associated with fracture severity. Average payments for closed fractures were $16,775 (95% CI: $16,668 - $16,882), whereas Gustilo III fractures averaged $41,206 (95% CI: $38,795 - $43,617). Complications resulted in incremental commercial payments over the 12 months post-index ranging from $5,200 for pain, to $27,510 for infection. An average incremental payment of $13,577 (95%CI: $9,692-$17,462) was observed for patients that required reoperation.

**Table 5:** 5A: Index commercial payments for ankle fracture surgery, by fracture severity and site of care. 5B: Incremental 12-month commercial payments in patients with continued pain or complications from ankle fracture surgery

**5 A: Index Payment for Ankle Fracture Treatment**	
** By Fracture Severity**	
Closed	$ 16,775 (95% CI: $ 16,668 - $ 16,882)
Gustilo I-II	$ 27,216 (95% CI: $ 26,322 - $ 28,111)
Gustilo III	$ 41,206 (95% CI: $ 38,795 - $ 43,617)
** By Site of Care**	
ASC	$ 9,821 (95% CI: $ 9,697 - $ 9,945)
Outpatient	$ 16,444 (95% CI: $ 16,317 - $ 16,571)
Inpatient	$ 28,169 (95% CI: $ 27,780 - $ 28,559)
**5B: Incremental 12-Month Postoperative Payments, by Complication Type**	
** Delayed Healing**	$ 8,058 (95% CI: $ 3,716 - $ 12,400)
** Nonunion**	$ 5,395 (95% CI: $ 1,122 - $ 9,669)
** Infection**	$ 27,510 (95% CI: $ 21,759 - $ 33,261)
** Pain**	$ 5,200 (95% CI: $ 4,261 - $ 6,139)
** Reoperation**	$ 13,577 (95% CI: $ 9,692 - $ 17,462)

## Discussion

Trends of ankle fracture incidence in the United States are not well documented because these fractures are mostly treated in the outpatient setting and are therefore not consistently captured in large hospital nationwide databases. Our analysis used nationally representative claims databases to estimate an incidence of approximately 14.1 cases per 10,000 patient-years, of which 3.3 required surgical care. A further in-depth analysis of patients that required surgery showed that 37% of patients less than 65 (commercially-insured), and half of the Medicare patients (65 and above), presented with 2 or more fracture types, thus potentially complex cases. Post-operative complications were surprisingly common. Residual pain was very frequent: between 30% and 40% of patients experienced continuous pain more than 3 months after surgery. Joint derangements were also very common, with 25% of commercially-insured and 17% of Medicare patients reporting joint derangements in the post-operative period. Re-fracture and infection affected 11% and 10% of Medicare patients and 4% (each) of commercial patients, all other complications each affecting individually less than 5% of patients.  Approximately 10% of patients underwent a secondary procedure (reoperation). Revision procedures, defined as a new ankle surgery or procedure at least 3-weeks after index, affected approximately 6% of patients. Interestingly, the majority of patients with joint derangements or pain did not have a reoperation within 12 months of index, suggesting conservative management of these post-operative conditions.

The increased risk of complications in Medicare patients vs. commercial patients may simply reflect age differences, and the fact that Medicare patients presented with far more comorbidities, as shown in Table 2. Hypertension (commercial: 19.9%, Medicare: 65.1%) and diabetes (commercial: 7.5%, Medicare: 27.2%) are 2 examples, but most rates of comorbidities were significantly greater in the Medicare cohort vs. the commercial cohort. These comorbidities have been shown to affect bone healing rates and increase risk for postoperative complications [27].

Our findings highlight the potential significant societal impact of ankle fracture in the United States: with relative high incidence rates, and complication and residual pain rates, ankle fracture may represent lasting burden on patients and healthcare systems.

Another recent analysis by Scheer et al. estimated the incidence of ankle fracture reported to emergency rooms at 4.2 per 10,000 person-years [1]. We found 14.1 fractures per 10,000 – observed in all care settings (not only emergency departments), of which 3.3 required surgical intervention. The 4.2 value reported by Scheer et al. is lower than our overall estimate of 14.1, possibly because not all fractures go to emergency departments, but higher than our estimate of fractures requiring surgery (3.3), as not all fractures identified in the emergency department may require a surgical fixation. Milstrey et al. evaluated distal fibula fractures in the German Federal Statistical Office, from 2005 to 2019, and reported an estimated 7.4 per 10,000 person/year (+/- 3.2) [28]. In our analysis, malleolus, bimalleolar and trimalleolar fractures (involving therefore the distal fibula) represented approximately 50% of all ankle fractures, thus about half of the 14.1 fractures per 10,000 person/year. Again, our estimate was therefore very close to the 7.4 reported in Germany. Finally, a Danish study from 2018 also reported incidence of overall ankle fracture of 16.8 per 10,000 patient-years [21].

The trends identified in our analysis, with increasing fractures in pediatrics, decreasing in adulthood until age 30 and increasing again, especially in female patients, with increasing age, has also been observed in Denmark by Elsoe et al. [21]. There are many causes for ankle fractures, childhood fractures are mostly linked to sports and high energy activity done in the presence of open growth plates, whereas fractures in older patients may be linked to increased rates of osteoporosis, propensity for falls, increased weight, and polypharmacy, which can lead to poor bone quality [29].

Surgery for the treatment of ankle fracture is particularly important when stability is compromised. Ankle fractures are commonly managed using open reduction and internal fixation. Whereas the long-term outcomes of these procedures are favorable, multiple articles have identified ongoing pain and stiffness in the early post-operative periods, as we have in our study. Specifically, Beckenkamp et al. reported reduced activity in patients with ankle fractures at 6-months post-index, due to ongoing stiffness and pain [30]. Another recent study further confirmed a slow return to pre-fracture activity and limitations in range of motion following surgery, up to 12 months post-index [31]. In our study, nearly a third of the commercial population, and more than a third of the Medicare population, had continuous pain. Our findings are therefore aligned with prior reports of relative slow recovery. An analysis of pain medication utilization was not included in this study and may constitute a limitation of this work.

The healthcare costs of ankle fracture, from the perspective of the payer, are not well documented. Pasic et al. evaluated cost of care from the perspective of the provider, reporting, as expected, higher costs in the inpatient vs. outpatient setting [32]. Our study identified the same trend, with ASCs having lower insurance payments than in- or outpatient sites. Severity of fracture was also a key determinant in index payments, with Gustilo III fractures being 2.5-fold higher payments than closed fractures. The difficulty of treating complex cases also explains why inpatient payments are higher: 85% of all Gustilo III fractures were treated in the inpatient setting, whereas more than 98% of all cases treated in the ASCs or outpatient cases were closed fractures. Incremental insurance payment analyses were conducted in the 12 months post-surgery, to understand the financial impact of post-operative complications. Continued pain, by itself, was associated with incremental payments exceeding $5K. Infection averaged an incremental payment of $27K.

The limitations of this study are mainly those inherent to the use of administrative claims data, which are not collected specifically for research purposes. Administrative data lack information, particularly clinical variables, limiting the inferences that can be made. They are also at risk of having clerical inaccuracies, recording bias secondary to financial incentives, and temporal changes in billing codes [33, 34]. As noted above, an addition limitation includes the fact that prescription patterns in relations to ankle fracture treatments were not analyzed in this study. This study is also limited in that the findings from this commercially-insured US population may not be generalizable to other patients with ankle fracture surgery, particularly those without health insurance or with other types of health insurance, and to patients in other countries. Despite these limitations, this study provides an informative overview of the experience of care among commercially-insured and Medicare-insured US patients with ankle fracture surgery. A key strength of our analysis is the inclusion of cases identified in the outpatient setting, in addition to those reported in the inpatient and emergency departments. Ankle fractures reported only in the outpatient setting are not consistently included in other studies that rely on hospital data, but as observed in our study, represent a large volume of patients [1, 35].

### Conclusion

Our study identified an incidence of ankle fracture in the 2016–2019 time period of 14.1 per 10,000 patient-years, of which 3.3 required surgery. Complication rates were high, with approximately 10% of patients requiring a secondary surgery. Residual pain was the main complication and affected nearly a third of all patients. Due to its high incidence, and high complications at 12 months post-surgery, ankle fracture may have a significant societal impact in terms of patient quality of life and return to normal activities, and healthcare burden.

## Supplementary Information

**Additional file 1.**

## Data Availability

The data that support the findings of this study are available from IBM^®^ MarketScan^®^ but restrictions apply to the availability of these data, which were used under license for the current study, and so are not publicly available. Aggregate data are however available from the authors (CEH) upon reasonable request and with permission of IBM^®^ MarketScan^®^.

## Abbreviations

ASA: American Society of Anesthesiologists
ASC: Ambulatory surgery center
CCAE: Commercial Claims and Encounters
CI: Confidence interval
CPI: Consumer Price Index
CPT: Common Procedural Terminology
ECI: Elixhauser Comorbidity Index
ICD: International Classification of Disease
OR: Odds ratio
ORIF: Open reduction and internal fixation
SD: Standard deviation
SSI: Surgical site infections
