# Supplementary material for: Incidence, costs and post-operative complications following ankle fracture – A US claims database analysis
Source: BMC Musculoskelet Disord. 2022 Dec 26;23:1129. doi: 10.1186/s12891-022-06095-x (PMC9791722; doi:10.1186/s12891-022-06095-x)
Supplement: Supplementary file 1 — Additional file 1. [file 12891_2022_6095_MOESM1_ESM.pdf]

Variable Name	Code	Code Type	Code Description
Ankle Fracture Diagnosis	S82.301A	ICD-10 CM	UNSPECIFIED FRACTURE OF LOWER END OF RIGHT TIBIA INITIAL ENCOUNTER FOR CLOSED FRACTURE
Ankle Fracture Diagnosis	S82.301B	ICD-10 CM	UNSPECIFIED FRACTURE OF LOWER END OF RIGHT TIBIA INITIAL ENCOUNTER FOR OPEN FRACTURE TYPE I OR II
Ankle Fracture Diagnosis	S82.301C	ICD-10 CM	UNSPECIFIED FRACTURE OF LOWER END OF RIGHT TIBIA INITIAL ENCOUNTER FOR OPEN FRACTURE TYPE IIIA IIIB OR IIIC
Ankle Fracture Diagnosis	S82.302A	ICD-10 CM	UNSPECIFIED FRACTURE OF LOWER END OF LEFT TIBIA INITIAL ENCOUNTER FOR CLOSED FRACTURE
Ankle Fracture Diagnosis	S82.302B	ICD-10 CM	UNSPECIFIED FRACTURE OF LOWER END OF LEFT TIBIA INITIAL ENCOUNTER FOR OPEN FRACTURE TYPE I OR II
Ankle Fracture Diagnosis	S82.302C	ICD-10 CM	UNSPECIFIED FRACTURE OF LOWER END OF LEFT TIBIA INITIAL ENCOUNTER FOR OPEN FRACTURE TYPE IIIA IIIB OR IIIC
Ankle Fracture Diagnosis	S82.309A	ICD-10 CM	UNSPECIFIED FRACTURE OF LOWER END OF UNSPECIFIED TIBIA INITIAL ENCOUNTER FOR CLOSED FRACTURE
Ankle Fracture Diagnosis	S82.309B	ICD-10 CM	UNSPECIFIED FRACTURE OF LOWER END OF UNSPECIFIED TIBIA INITIAL ENCOUNTER FOR OPEN FRACTURE TYPE I OR II
Ankle Fracture Diagnosis	S82.309C	ICD-10 CM	UNSPECIFIED FRACTURE OF LOWER END OF UNSPECIFIED TIBIA INITIAL ENCOUNTER FOR OPEN FRACTURE TYPE IIIA IIIB OR IIIC
Ankle Fracture Diagnosis	S82.311A	ICD-10 CM	TORUS FRACTURE OF LOWER END OF RIGHT TIBIA INITIAL ENCOUNTER FOR CLOSED FRACTURE
Ankle Fracture Diagnosis	S82.312A	ICD-10 CM	TORUS FRACTURE OF LOWER END OF LEFT TIBIA INITIAL ENCOUNTER FOR CLOSED FRACTURE
Ankle Fracture Diagnosis	S82.319A	ICD-10 CM	TORUS FRACTURE OF LOWER END OF UNSPECIFIED TIBIA INITIAL ENCOUNTER FOR CLOSED FRACTURE
Ankle Fracture Diagnosis	S82.391A	ICD-10 CM	OTHER FRACTURE OF LOWER END OF RIGHT TIBIA INITIAL ENCOUNTER FOR CLOSED FRACTURE
Ankle Fracture Diagnosis	S82.391B	ICD-10 CM	OTHER FRACTURE OF LOWER END OF RIGHT TIBIA INITIAL ENCOUNTER FOR OPEN FRACTURE TYPE I OR II
Ankle Fracture Diagnosis	S82.391C	ICD-10 CM	OTHER FRACTURE OF LOWER END OF RIGHT TIBIA INITIAL ENCOUNTER FOR OPEN FRACTURE TYPE IIIA IIIB OR IIIC
Ankle Fracture Diagnosis	S82.392A	ICD-10 CM	OTHER FRACTURE OF LOWER END OF LEFT TIBIA INITIAL ENCOUNTER FOR CLOSED FRACTURE
Ankle Fracture Diagnosis	S82.392B	ICD-10 CM	OTHER FRACTURE OF LOWER END OF LEFT TIBIA INITIAL ENCOUNTER FOR OPEN FRACTURE TYPE I OR II
Ankle Fracture Diagnosis	S82.392C	ICD-10 CM	OTHER FRACTURE OF LOWER END OF LEFT TIBIA INITIAL ENCOUNTER FOR OPEN FRACTURE TYPE IIIA IIIB OR IIIC
Ankle Fracture Diagnosis	S82.399A	ICD-10 CM	OTHER FRACTURE OF LOWER END OF UNSPECIFIED TIBIA INITIAL ENCOUNTER FOR CLOSED FRACTURE
Ankle Fracture Diagnosis	S82.399B	ICD-10 CM	OTHER FRACTURE OF LOWER END OF UNSPECIFIED TIBIA INITIAL ENCOUNTER FOR OPEN FRACTURE TYPE I OR II
Ankle Fracture Diagnosis	S82.399C	ICD-10 CM	OTHER FRACTURE OF LOWER END OF UNSPECIFIED TIBIA INITIAL ENCOUNTER FOR OPEN FRACTURE TYPE IIIA IIIB OR IIIC
Ankle Fracture Diagnosis	S82.51XA	ICD-10 CM	DISPLACED FRACTURE OF MEDIAL MALLEOLUS OF RIGHT TIBIA INITIAL ENCOUNTER FOR CLOSED FRACTURE
Ankle Fracture Diagnosis	S82.51XB	ICD-10 CM	DISPLACED FRACTURE OF MEDIAL MALLEOLUS OF RIGHT TIBIA INITIAL ENCOUNTER FOR OPEN FRACTURE TYPE I OR II
Ankle Fracture Diagnosis	S82.51XC	ICD-10 CM	DISPLACED FRACTURE OF MEDIAL MALLEOLUS OF RIGHT TIBIA INITIAL ENCOUNTER FOR OPEN FRACTURE TYPE IIIA IIIB OR IIIC
Ankle Fracture Diagnosis	S82.52XA	ICD-10 CM	DISPLACED FRACTURE OF MEDIAL MALLEOLUS OF LEFT TIBIA INITIAL ENCOUNTER FOR CLOSED FRACTURE
Ankle Fracture Diagnosis	S82.52XB	ICD-10 CM	DISPLACED FRACTURE OF MEDIAL MALLEOLUS OF LEFT TIBIA INITIAL ENCOUNTER FOR OPEN FRACTURE TYPE I OR II
Ankle Fracture Diagnosis	S82.52XC	ICD-10 CM	DISPLACED FRACTURE OF MEDIAL MALLEOLUS OF LEFT TIBIA INITIAL ENCOUNTER FOR OPEN FRACTURE TYPE IIIA IIIB OR IIIC
Ankle Fracture Diagnosis	S82.53XA	ICD-10 CM	DISPLACED FRACTURE OF MEDIAL MALLEOLUS OF UNSPECIFIED TIBIA INITIAL ENCOUNTER FOR CLOSED FRACTURE
Ankle Fracture Diagnosis	S82.53XB	ICD-10 CM	DISPLACED FRACTURE OF MEDIAL MALLEOLUS OF UNSPECIFIED TIBIA INITIAL ENCOUNTER FOR OPEN FRACTURE TYPE I OR II
Ankle Fracture Diagnosis	S82.53XC	ICD-10 CM	DISPLACED FRACTURE OF MEDIAL MALLEOLUS OF UNSPECIFIED TIBIA INITIAL ENCOUNTER FOR OPEN FRACTURE TYPE IIIA IIIB OR IIIC
Ankle Fracture Diagnosis	S82.54XA	ICD-10 CM	NONDISPLACED FRACTURE OF MEDIAL MALLEOLUS OF RIGHT TIBIA INITIAL ENCOUNTER FOR CLOSED FRACTURE
Ankle Fracture Diagnosis	S82.54XB	ICD-10 CM	NONDISPLACED FRACTURE OF MEDIAL MALLEOLUS OF RIGHT TIBIA INITIAL ENCOUNTER FOR OPEN FRACTURE TYPE I OR II
Ankle Fracture Diagnosis	S82.54XC	ICD-10 CM	NONDISPLACED FRACTURE OF MEDIAL MALLEOLUS OF RIGHT TIBIA INITIAL ENCOUNTER FOR OPEN FRACTURE TYPE IIIA IIIB OR IIIC
Ankle Fracture Diagnosis	S82.55XA	ICD-10 CM	NONDISPLACED FRACTURE OF MEDIAL MALLEOLUS OF LEFT TIBIA INITIAL ENCOUNTER FOR CLOSED FRACTURE
Ankle Fracture Diagnosis	S82.55XB	ICD-10 CM	NONDISPLACED FRACTURE OF MEDIAL MALLEOLUS OF LEFT TIBIA INITIAL ENCOUNTER FOR OPEN FRACTURE TYPE I OR II

Ankle Fracture Diagnosis	S82.55XC	ICD-10 CM	NONDISPLACED FRACTURE OF MEDIAL MALLEOLUS OF LEFT TIBIA INITIAL ENCOUNTER FOR OPEN FRACTURE TYPE IIIA IIIB OR IIIC
Ankle Fracture Diagnosis	S82.56XA	ICD-10 CM	NONDISPLACED FRACTURE OF MEDIAL MALLEOLUS OF UNSPECIFIED TIBIA INITIAL ENCOUNTER FOR CLOSED FRACTURE
Ankle Fracture Diagnosis	S82.56XB	ICD-10 CM	NONDISPLACED FRACTURE OF MEDIAL MALLEOLUS OF UNSPECIFIED TIBIA INITIAL ENCOUNTER FOR OPEN FRACTURE TYPE I OR II
Ankle Fracture Diagnosis	S82.56XC	ICD-10 CM	NONDISPLACED FRACTURE OF MEDIAL MALLEOLUS OF UNSPECIFIED TIBIA INITIAL ENCOUNTER FOR OPEN FRACTURE TYPE IIIA IIIB OR IIIC
Ankle Fracture Diagnosis	S82.61XA	ICD-10 CM	DISPLACED FRACTURE OF LATERAL MALLEOLUS OF RIGHT FIBULA INITIAL ENCOUNTER FOR CLOSED FRACTURE
Ankle Fracture Diagnosis	S82.61XB	ICD-10 CM	DISPLACED FRACTURE OF LATERAL MALLEOLUS OF RIGHT FIBULA INITIAL ENCOUNTER FOR OPEN FRACTURE TYPE I OR II
Ankle Fracture Diagnosis	S82.61XC	ICD-10 CM	DISPLACED FRACTURE OF LATERAL MALLEOLUS OF RIGHT FIBULA INITIAL ENCOUNTER FOR OPEN FRACTURE TYPE IIIA IIIB OR IIIC
Ankle Fracture Diagnosis	S82.62XA	ICD-10 CM	DISPLACED FRACTURE OF LATERAL MALLEOLUS OF LEFT FIBULA INITIAL ENCOUNTER FOR CLOSED FRACTURE
Ankle Fracture Diagnosis	S82.62XB	ICD-10 CM	DISPLACED FRACTURE OF LATERAL MALLEOLUS OF LEFT FIBULA INITIAL ENCOUNTER FOR OPEN FRACTURE TYPE I OR II
Ankle Fracture Diagnosis	S82.62XC	ICD-10 CM	DISPLACED FRACTURE OF LATERAL MALLEOLUS OF LEFT FIBULA INITIAL ENCOUNTER FOR OPEN FRACTURE TYPE IIIA IIIB OR IIIC
Ankle Fracture Diagnosis	S82.63XA	ICD-10 CM	DISPLACED FRACTURE OF LATERAL MALLEOLUS OF UNSPECIFIED FIBULA INITIAL ENCOUNTER FOR CLOSED FRACTURE
Ankle Fracture Diagnosis	S82.63XB	ICD-10 CM	DISPLACED FRACTURE OF LATERAL MALLEOLUS OF UNSPECIFIED FIBULA INITIAL ENCOUNTER FOR OPEN FRACTURE TYPE I OR II
Ankle Fracture Diagnosis	S82.63XC	ICD-10 CM	DISPLACED FRACTURE OF LATERAL MALLEOLUS OF UNSPECIFIED FIBULA INITIAL ENCOUNTER FOR OPEN FRACTURE TYPE IIIA IIIB OR IIIC
Ankle Fracture Diagnosis	S82.64XA	ICD-10 CM	NONDISPLACED FRACTURE OF LATERAL MALLEOLUS OF RIGHT FIBULA INITIAL ENCOUNTER FOR CLOSED FRACTURE
Ankle Fracture Diagnosis	S82.64XB	ICD-10 CM	NONDISPLACED FRACTURE OF LATERAL MALLEOLUS OF RIGHT FIBULA INITIAL ENCOUNTER FOR OPEN FRACTURE TYPE I OR II
Ankle Fracture Diagnosis	S82.64XC	ICD-10 CM	NONDISPLACED FRACTURE OF LATERAL MALLEOLUS OF RIGHT FIBULA INITIAL ENCOUNTER FOR OPEN FRACTURE TYPE IIIA IIIB OR IIIC
Ankle Fracture Diagnosis	S82.65XA	ICD-10 CM	NONDISPLACED FRACTURE OF LATERAL MALLEOLUS OF LEFT FIBULA INITIAL ENCOUNTER FOR CLOSED FRACTURE
Ankle Fracture Diagnosis	S82.65XB	ICD-10 CM	NONDISPLACED FRACTURE OF LATERAL MALLEOLUS OF LEFT FIBULA INITIAL ENCOUNTER FOR OPEN FRACTURE TYPE I OR II
Ankle Fracture Diagnosis	S82.65XC	ICD-10 CM	NONDISPLACED FRACTURE OF LATERAL MALLEOLUS OF LEFT FIBULA INITIAL ENCOUNTER FOR OPEN FRACTURE TYPE IIIA IIIB OR IIIC
Ankle Fracture Diagnosis	S82.66XA	ICD-10 CM	NONDISPLACED FRACTURE OF LATERAL MALLEOLUS OF UNSPECIFIED FIBULA INITIAL ENCOUNTER FOR CLOSED FRACTURE
Ankle Fracture Diagnosis	S82.66XB	ICD-10 CM	NONDISPLACED FRACTURE OF LATERAL MALLEOLUS OF UNSPECIFIED FIBULA INITIAL ENCOUNTER FOR OPEN FRACTURE TYPE I OR II
Ankle Fracture Diagnosis	S82.66XC	ICD-10 CM	NONDISPLACED FRACTURE OF LATERAL MALLEOLUS OF UNSPECIFIED FIBULA INITIAL ENCOUNTER FOR OPEN FRACTURE TYPE IIIA IIIB OR IIIC
Ankle Fracture Diagnosis	S82.841A	ICD-10 CM	DISPLACED BIMALLEOLAR FRACTURE OF RIGHT LOWER LEG INITIAL ENCOUNTER FOR CLOSED FRACTURE
Ankle Fracture Diagnosis	S82.841B	ICD-10 CM	DISPLACED BIMALLEOLAR FRACTURE OF RIGHT LOWER LEG INITIAL ENCOUNTER FOR OPEN FRACTURE TYPE I OR II
Ankle Fracture Diagnosis	S82.841C	ICD-10 CM	DISPLACED BIMALLEOLAR FRACTURE OF RIGHT LOWER LEG INITIAL ENCOUNTER FOR OPEN FRACTURE TYPE IIIA IIIB OR IIIC
Ankle Fracture Diagnosis	S82.842A	ICD-10 CM	DISPLACED BIMALLEOLAR FRACTURE OF LEFT LOWER LEG INITIAL ENCOUNTER FOR CLOSED FRACTURE
Ankle Fracture Diagnosis	S82.842B	ICD-10 CM	DISPLACED BIMALLEOLAR FRACTURE OF LEFT LOWER LEG INITIAL ENCOUNTER FOR OPEN FRACTURE TYPE I OR II
Ankle Fracture Diagnosis	S82.842C	ICD-10 CM	DISPLACED BIMALLEOLAR FRACTURE OF LEFT LOWER LEG INITIAL ENCOUNTER FOR OPEN FRACTURE TYPE IIIA IIIB OR IIIC
Ankle Fracture Diagnosis	S82.843A	ICD-10 CM	DISPLACED BIMALLEOLAR FRACTURE OF UNSPECIFIED LOWER LEG INITIAL ENCOUNTER FOR CLOSED FRACTURE
Ankle Fracture Diagnosis	S82.843B	ICD-10 CM	DISPLACED BIMALLEOLAR FRACTURE OF UNSPECIFIED LOWER LEG INITIAL ENCOUNTER FOR OPEN FRACTURE TYPE I OR II
Ankle Fracture Diagnosis	S82.843C	ICD-10 CM	DISPLACED BIMALLEOLAR FRACTURE OF UNSPECIFIED LOWER LEG INITIAL ENCOUNTER FOR OPEN FRACTURE TYPE IIIA IIIB OR IIIC
Ankle Fracture Diagnosis	S82.844A	ICD-10 CM	NONDISPLACED BIMALLEOLAR FRACTURE OF RIGHT LOWER LEG INITIAL ENCOUNTER FOR CLOSED FRACTURE
Ankle Fracture Diagnosis	S82.844B	ICD-10 CM	NONDISPLACED BIMALLEOLAR FRACTURE OF RIGHT LOWER LEG INITIAL ENCOUNTER FOR OPEN FRACTURE TYPE I OR II
Ankle Fracture Diagnosis	S82.844C	ICD-10 CM	NONDISPLACED BIMALLEOLAR FRACTURE OF RIGHT LOWER LEG INITIAL ENCOUNTER FOR OPEN FRACTURE TYPE IIIA IIIB OR IIIC

Ankle Fracture Diagnosis	S82.845A	ICD-10 CM	NONDISPLACED BIMALLEOLAR FRACTURE OF LEFT LOWER LEG INITIAL ENCOUNTER FOR CLOSED FRACTURE
Ankle Fracture Diagnosis	S82.845B	ICD-10 CM	NONDISPLACED BIMALLEOLAR FRACTURE OF LEFT LOWER LEG INITIAL ENCOUNTER FOR OPEN FRACTURE TYPE I OR II
Ankle Fracture Diagnosis	S82.845C	ICD-10 CM	NONDISPLACED BIMALLEOLAR FRACTURE OF LEFT LOWER LEG INITIAL ENCOUNTER FOR OPEN FRACTURE TYPE IIIA IIIB OR IIIC
Ankle Fracture Diagnosis	S82.846A	ICD-10 CM	NONDISPLACED BIMALLEOLAR FRACTURE OF UNSPECIFIED LOWER LEG INITIAL ENCOUNTER FOR CLOSED FRACTURE
Ankle Fracture Diagnosis	S82.846B	ICD-10 CM	NONDISPLACED BIMALLEOLAR FRACTURE OF UNSPECIFIED LOWER LEG INITIAL ENCOUNTER FOR OPEN FRACTURE TYPE I OR II
Ankle Fracture Diagnosis	S82.846C	ICD-10 CM	NONDISPLACED BIMALLEOLAR FRACTURE OF UNSPECIFIED LOWER LEG INITIAL ENCOUNTER FOR OPEN FRACTURE TYPE IIIA IIIB OR IIIC
Ankle Fracture Diagnosis	S82.851A	ICD-10 CM	DISPLACED TRIMALLEOLAR FRACTURE OF RIGHT LOWER LEG INITIAL ENCOUNTER FOR CLOSED FRACTURE
Ankle Fracture Diagnosis	S82.851B	ICD-10 CM	DISPLACED TRIMALLEOLAR FRACTURE OF RIGHT LOWER LEG INITIAL ENCOUNTER FOR OPEN FRACTURE TYPE I OR II
Ankle Fracture Diagnosis	S82.851C	ICD-10 CM	DISPLACED TRIMALLEOLAR FRACTURE OF RIGHT LOWER LEG INITIAL ENCOUNTER FOR OPEN FRACTURE TYPE IIIA IIIB OR IIIC
Ankle Fracture Diagnosis	S82.852A	ICD-10 CM	DISPLACED TRIMALLEOLAR FRACTURE OF LEFT LOWER LEG INITIAL ENCOUNTER FOR CLOSED FRACTURE
Ankle Fracture Diagnosis	S82.852B	ICD-10 CM	DISPLACED TRIMALLEOLAR FRACTURE OF LEFT LOWER LEG INITIAL ENCOUNTER FOR OPEN FRACTURE TYPE I OR II
Ankle Fracture Diagnosis	S82.852C	ICD-10 CM	DISPLACED TRIMALLEOLAR FRACTURE OF LEFT LOWER LEG INITIAL ENCOUNTER FOR OPEN FRACTURE TYPE IIIA IIIB OR IIIC
Ankle Fracture Diagnosis	S82.853A	ICD-10 CM	DISPLACED TRIMALLEOLAR FRACTURE OF UNSPECIFIED LOWER LEG INITIAL ENCOUNTER FOR CLOSED FRACTURE
Ankle Fracture Diagnosis	S82.853B	ICD-10 CM	DISPLACED TRIMALLEOLAR FRACTURE OF UNSPECIFIED LOWER LEG INITIAL ENCOUNTER FOR OPEN FRACTURE TYPE I OR II
Ankle Fracture Diagnosis	S82.853C	ICD-10 CM	DISPLACED TRIMALLEOLAR FRACTURE OF UNSPECIFIED LOWER LEG INITIAL ENCOUNTER FOR OPEN FRACTURE TYPE IIIA IIIB OR IIIC
Ankle Fracture Diagnosis	S82.854A	ICD-10 CM	NONDISPLACED TRIMALLEOLAR FRACTURE OF RIGHT LOWER LEG INITIAL ENCOUNTER FOR CLOSED FRACTURE
Ankle Fracture Diagnosis	S82.854B	ICD-10 CM	NONDISPLACED TRIMALLEOLAR FRACTURE OF RIGHT LOWER LEG INITIAL ENCOUNTER FOR OPEN FRACTURE TYPE I OR II
Ankle Fracture Diagnosis	S82.854C	ICD-10 CM	NONDISPLACED TRIMALLEOLAR FRACTURE OF RIGHT LOWER LEG INITIAL ENCOUNTER FOR OPEN FRACTURE TYPE IIIA IIIB OR IIIC
Ankle Fracture Diagnosis	S82.855A	ICD-10 CM	NONDISPLACED TRIMALLEOLAR FRACTURE OF LEFT LOWER LEG INITIAL ENCOUNTER FOR CLOSED FRACTURE
Ankle Fracture Diagnosis	S82.855B	ICD-10 CM	NONDISPLACED TRIMALLEOLAR FRACTURE OF LEFT LOWER LEG INITIAL ENCOUNTER FOR OPEN FRACTURE TYPE I OR II
Ankle Fracture Diagnosis	S82.855C	ICD-10 CM	NONDISPLACED TRIMALLEOLAR FRACTURE OF LEFT LOWER LEG INITIAL ENCOUNTER FOR OPEN FRACTURE TYPE IIIA IIIB OR IIIC
Ankle Fracture Diagnosis	S82.856A	ICD-10 CM	NONDISPLACED TRIMALLEOLAR FRACTURE OF UNSPECIFIED LOWER LEG INITIAL ENCOUNTER FOR CLOSED FRACTURE
Ankle Fracture Diagnosis	S82.856B	ICD-10 CM	NONDISPLACED TRIMALLEOLAR FRACTURE OF UNSPECIFIED LOWER LEG INITIAL ENCOUNTER FOR OPEN FRACTURE TYPE I OR II
Ankle Fracture Diagnosis	S82.856C	ICD-10 CM	NONDISPLACED TRIMALLEOLAR FRACTURE OF UNSPECIFIED LOWER LEG INITIAL ENCOUNTER FOR OPEN FRACTURE TYPE IIIA IIIB OR IIIC
Ankle Fracture Diagnosis	S82.861A	ICD-10 CM	DISPLACED MAISONNEUVE'S FRACTURE OF RIGHT LEG INITIAL ENCOUNTER FOR CLOSED FRACTURE
Ankle Fracture Diagnosis	S82.861B	ICD-10 CM	DISPLACED MAISONNEUVE'S FRACTURE OF RIGHT LEG INITIAL ENCOUNTER FOR OPEN FRACTURE TYPE I OR II
Ankle Fracture Diagnosis	S82.861C	ICD-10 CM	DISPLACED MAISONNEUVE'S FRACTURE OF RIGHT LEG INITIAL ENCOUNTER FOR OPEN FRACTURE TYPE IIIA IIIB OR IIIC
Ankle Fracture Diagnosis	S82.862A	ICD-10 CM	DISPLACED MAISONNEUVE'S FRACTURE OF LEFT LEG INITIAL ENCOUNTER FOR CLOSED FRACTURE
Ankle Fracture Diagnosis	S82.862B	ICD-10 CM	DISPLACED MAISONNEUVE'S FRACTURE OF LEFT LEG INITIAL ENCOUNTER FOR OPEN FRACTURE TYPE I OR II
Ankle Fracture Diagnosis	S82.862C	ICD-10 CM	DISPLACED MAISONNEUVE'S FRACTURE OF LEFT LEG INITIAL ENCOUNTER FOR OPEN FRACTURE TYPE IIIA IIIB OR IIIC
Ankle Fracture Diagnosis	S82.863A	ICD-10 CM	DISPLACED MAISONNEUVE'S FRACTURE OF UNSPECIFIED LEG INITIAL ENCOUNTER FOR CLOSED FRACTURE
Ankle Fracture Diagnosis	S82.863B	ICD-10 CM	DISPLACED MAISONNEUVE'S FRACTURE OF UNSPECIFIED LEG INITIAL ENCOUNTER FOR OPEN FRACTURE TYPE I OR II
Ankle Fracture Diagnosis	S82.863C	ICD-10 CM	DISPLACED MAISONNEUVE'S FRACTURE OF UNSPECIFIED LEG INITIAL ENCOUNTER FOR OPEN FRACTURE TYPE IIIA IIIB OR IIIC
Ankle Fracture Diagnosis	S82.864A	ICD-10 CM	NONDISPLACED MAISONNEUVE'S FRACTURE OF RIGHT LEG INITIAL ENCOUNTER FOR CLOSED FRACTURE

Ankle Fracture Diagnosis	S82.864B	ICD-10 CM	NONDISPLACED MAISONNEUVE'S FRACTURE OF RIGHT LEG INITIAL ENCOUNTER FOR OPEN FRACTURE TYPE I OR II
Ankle Fracture Diagnosis	S82.864C	ICD-10 CM	NONDISPLACED MAISONNEUVE'S FRACTURE OF RIGHT LEG INITIAL ENCOUNTER FOR OPEN FRACTURE TYPE IIIA IIIB OR IIIC
Ankle Fracture Diagnosis	S82.865A	ICD-10 CM	NONDISPLACED MAISONNEUVE'S FRACTURE OF LEFT LEG INITIAL ENCOUNTER FOR CLOSED FRACTURE
Ankle Fracture Diagnosis	S82.865B	ICD-10 CM	NONDISPLACED MAISONNEUVE'S FRACTURE OF LEFT LEG INITIAL ENCOUNTER FOR OPEN FRACTURE TYPE I OR II
Ankle Fracture Diagnosis	S82.865C	ICD-10 CM	NONDISPLACED MAISONNEUVE'S FRACTURE OF LEFT LEG INITIAL ENCOUNTER FOR OPEN FRACTURE TYPE IIIA IIIB OR IIIC
Ankle Fracture Diagnosis	S82.866A	ICD-10 CM	NONDISPLACED MAISONNEUVE'S FRACTURE OF UNSPECIFIED LEG INITIAL ENCOUNTER FOR CLOSED FRACTURE
Ankle Fracture Diagnosis	S82.866B	ICD-10 CM	NONDISPLACED MAISONNEUVE'S FRACTURE OF UNSPECIFIED LEG INITIAL ENCOUNTER FOR OPEN FRACTURE TYPE I OR II
Ankle Fracture Diagnosis	S82.866C	ICD-10 CM	NONDISPLACED MAISONNEUVE'S FRACTURE OF UNSPECIFIED LEG INITIAL ENCOUNTER FOR OPEN FRACTURE TYPE IIIA IIIB OR IIIC
Ankle Fracture Diagnosis	S82.871A	ICD-10 CM	DISPLACED PILON FRACTURE OF RIGHT TIBIA INITIAL ENCOUNTER FOR CLOSED FRACTURE
Ankle Fracture Diagnosis	S82.871B	ICD-10 CM	DISPLACED PILON FRACTURE OF RIGHT TIBIA INITIAL ENCOUNTER FOR OPEN FRACTURE TYPE I OR II
Ankle Fracture Diagnosis	S82.871C	ICD-10 CM	DISPLACED PILON FRACTURE OF RIGHT TIBIA INITIAL ENCOUNTER FOR OPEN FRACTURE TYPE IIIA IIIB OR IIIC
Ankle Fracture Diagnosis	S82.872A	ICD-10 CM	DISPLACED PILON FRACTURE OF LEFT TIBIA INITIAL ENCOUNTER FOR CLOSED FRACTURE
Ankle Fracture Diagnosis	S82.872B	ICD-10 CM	DISPLACED PILON FRACTURE OF LEFT TIBIA INITIAL ENCOUNTER FOR OPEN FRACTURE TYPE I OR II
Ankle Fracture Diagnosis	S82.872C	ICD-10 CM	DISPLACED PILON FRACTURE OF LEFT TIBIA INITIAL ENCOUNTER FOR OPEN FRACTURE TYPE IIIA IIIB OR IIIC
Ankle Fracture Diagnosis	S82.873A	ICD-10 CM	DISPLACED PILON FRACTURE OF UNSPECIFIED TIBIA INITIAL ENCOUNTER FOR CLOSED FRACTURE
Ankle Fracture Diagnosis	S82.873B	ICD-10 CM	DISPLACED PILON FRACTURE OF UNSPECIFIED TIBIA INITIAL ENCOUNTER FOR OPEN FRACTURE TYPE I OR II
Ankle Fracture Diagnosis	S82.873C	ICD-10 CM	DISPLACED PILON FRACTURE OF UNSPECIFIED TIBIA INITIAL ENCOUNTER FOR OPEN FRACTURE TYPE IIIA IIIB OR IIIC
Ankle Fracture Diagnosis	S82.874A	ICD-10 CM	NONDISPLACED PILON FRACTURE OF RIGHT TIBIA INITIAL ENCOUNTER FOR CLOSED FRACTURE
Ankle Fracture Diagnosis	S82.874B	ICD-10 CM	NONDISPLACED PILON FRACTURE OF RIGHT TIBIA INITIAL ENCOUNTER FOR OPEN FRACTURE TYPE I OR II
Ankle Fracture Diagnosis	S82.874C	ICD-10 CM	NONDISPLACED PILON FRACTURE OF RIGHT TIBIA INITIAL ENCOUNTER FOR OPEN FRACTURE TYPE IIIA IIIB OR IIIC
Ankle Fracture Diagnosis	S82.875A	ICD-10 CM	NONDISPLACED PILON FRACTURE OF LEFT TIBIA INITIAL ENCOUNTER FOR CLOSED FRACTURE
Ankle Fracture Diagnosis	S82.875B	ICD-10 CM	NONDISPLACED PILON FRACTURE OF LEFT TIBIA INITIAL ENCOUNTER FOR OPEN FRACTURE TYPE I OR II
Ankle Fracture Diagnosis	S82.875C	ICD-10 CM	NONDISPLACED PILON FRACTURE OF LEFT TIBIA INITIAL ENCOUNTER FOR OPEN FRACTURE TYPE IIIA IIIB OR IIIC
Ankle Fracture Diagnosis	S82.876A	ICD-10 CM	NONDISPLACED PILON FRACTURE OF UNSPECIFIED TIBIA INITIAL ENCOUNTER FOR CLOSED FRACTURE
Ankle Fracture Diagnosis	S82.876B	ICD-10 CM	NONDISPLACED PILON FRACTURE OF UNSPECIFIED TIBIA INITIAL ENCOUNTER FOR OPEN FRACTURE TYPE I OR II
Ankle Fracture Diagnosis	S82.876C	ICD-10 CM	NONDISPLACED PILON FRACTURE OF UNSPECIFIED TIBIA INITIAL ENCOUNTER FOR OPEN FRACTURE TYPE IIIA IIIB OR IIIC
Ankle Fracture Diagnosis	S82.891A	ICD-10 CM	OTHER FRACTURE OF RIGHT LOWER LEG INITIAL ENCOUNTER FOR CLOSED FRACTURE
Ankle Fracture Diagnosis	S82.891B	ICD-10 CM	OTHER FRACTURE OF RIGHT LOWER LEG INITIAL ENCOUNTER FOR OPEN FRACTURE TYPE I OR II
Ankle Fracture Diagnosis	S82.891C	ICD-10 CM	OTHER FRACTURE OF RIGHT LOWER LEG INITIAL ENCOUNTER FOR OPEN FRACTURE TYPE IIIA IIIB OR IIIC
Ankle Fracture Diagnosis	S82.892A	ICD-10 CM	OTHER FRACTURE OF LEFT LOWER LEG INITIAL ENCOUNTER FOR CLOSED FRACTURE
Ankle Fracture Diagnosis	S82.892B	ICD-10 CM	OTHER FRACTURE OF LEFT LOWER LEG INITIAL ENCOUNTER FOR OPEN FRACTURE TYPE I OR II
Ankle Fracture Diagnosis	S82.892C	ICD-10 CM	OTHER FRACTURE OF LEFT LOWER LEG INITIAL ENCOUNTER FOR OPEN FRACTURE TYPE IIIA IIIB OR IIIC
Ankle Fracture Diagnosis	S82.899A	ICD-10 CM	OTHER FRACTURE OF UNSPECIFIED LOWER LEG INITIAL ENCOUNTER FOR CLOSED FRACTURE
Ankle Fracture Diagnosis	S82.899B	ICD-10 CM	OTHER FRACTURE OF UNSPECIFIED LOWER LEG INITIAL ENCOUNTER FOR OPEN FRACTURE TYPE I OR II
Ankle Fracture Diagnosis	S82.899C	ICD-10 CM	OTHER FRACTURE OF UNSPECIFIED LOWER LEG INITIAL ENCOUNTER FOR OPEN FRACTURE TYPE IIIA IIIB OR IIIC
Ankle Fracture Procedure	27766	CPT	Open treatment of medial malleolus fracture includes internalfixation when performed
Ankle Fracture Procedure	27769	CPT	Open treatment of posterior malleolus fracture includes internalfixation when performed
Ankle Fracture Procedure	27792	CPT	Open treatment of distal fibular fracture (lateral malleolus) includes internal fixation when performed
Ankle Fracture Procedure	27814	CPT	Open treatment of bimalleolar ankle fracture (eg lateral and medialmalleoli or lateral and posterior malleoli or medial and posteriormalleoli) includes internal fixation when performed

Ankle Fracture Procedure	27822	CPT	Open treatment of trimalleolar ankle fracture includes internalfixation when performed medial and/or lateral malleolus; withoutfixation of posterior lip
Ankle Fracture Procedure	27823	CPT	Open treatment of trimalleolar ankle fracture includes internalfixation when performed medial and/or lateral malleolus; withfixation of posterior lip
Ankle Fracture Procedure	27826	CPT	Open treatment of fracture of weight bearing articular surface/portionof distal tibia (eg pilon or tibial plafond) with internal fixation when performed; of fibula only
Ankle Fracture Procedure	27827	CPT	Open treatment of fracture of weight bearing articular surface/portionof distal tibia (eg pilon or tibial plafond) with internal fixation when performed; of tibia only
Ankle Fracture Procedure	27828	CPT	Open treatment of fracture of weight bearing articular surface/portionof distal tibia (eg pilon or tibial plafond) with internal fixation when performed; of both tibia and fibula
Ankle Fracture Procedure	27829	CPT	Open treatment of distal tibiofibular joint (syndesmosis) disruption includes internal fixation when performed
Ankle Fracture Procedure	27870	CPT	Arthrodesis ankle open
Ankle Fracture Procedure	27871	CPT	Arthrodesis tibiofibular joint proximal or distal
Ankle Fracture Procedure	29899	CPT	Arthroscopy ankle (tibiotalar and fibulotalar joints) surgical; withankle arthrodesis
Arthritis	M14.879	ICD-10 CM	ARTHROPATHIES IN OTHER SPECIFIED DISEASES CLASSIFIED ELSEWHERE UNSPECIFIED ANKLE AND FOOT
Arthritis	M13.171	ICD-10 CM	MONOARTHRITIS NOT ELSEWHERE CLASSIFIED RIGHT ANKLE AND FOOT
Arthritis	M14.872	ICD-10 CM	ARTHROPATHIES IN OTHER SPECIFIED DISEASES CLASSIFIED ELSEWHERE LEFT ANKLE AND FOOT
Arthritis	M19.172	ICD-10 CM	POST-TRAUMATIC OSTEOARTHRITIS LEFT ANKLE AND FOOT
Arthritis	M14.871	ICD-10 CM	ARTHROPATHIES IN OTHER SPECIFIED DISEASES CLASSIFIED ELSEWHERE RIGHT ANKLE AND FOOT
Arthritis	M19.272	ICD-10 CM	SECONDARY OSTEOARTHRITIS LEFT ANKLE AND FOOT
Arthritis	M13.172	ICD-10 CM	MONOARTHRITIS NOT ELSEWHERE CLASSIFIED LEFT ANKLE AND FOOT
Arthritis	M19.171	ICD-10 CM	POST-TRAUMATIC OSTEOARTHRITIS RIGHT ANKLE AND FOOT
Arthritis	M19.279	ICD-10 CM	SECONDARY OSTEOARTHRITIS UNSPECIFIED ANKLE AND FOOT
Arthritis	M14.671	ICD-10 CM	CHARCOT'S JOINT RIGHT ANKLE AND FOOT
Arthritis	M14.679	ICD-10 CM	CHARCOT'S JOINT UNSPECIFIED ANKLE AND FOOT
Arthritis	M13.871	ICD-10 CM	OTHER SPECIFIED ARTHRITIS RIGHT ANKLE AND FOOT
Arthritis	M19.179	ICD-10 CM	POST-TRAUMATIC OSTEOARTHRITIS UNSPECIFIED ANKLE AND FOOT
Arthritis	M19.271	ICD-10 CM	SECONDARY OSTEOARTHRITIS RIGHT ANKLE AND FOOT
Arthritis	M13.879	ICD-10 CM	OTHER SPECIFIED ARTHRITIS UNSPECIFIED ANKLE AND FOOT
Arthritis	M14.672	ICD-10 CM	CHARCOT'S JOINT LEFT ANKLE AND FOOT
Arthritis	M13.872	ICD-10 CM	OTHER SPECIFIED ARTHRITIS LEFT ANKLE AND FOOT
Arthritis	M13.179	ICD-10 CM	MONOARTHRITIS NOT ELSEWHERE CLASSIFIED UNSPECIFIED ANKLE AND FOOT
Deformity Compliction	M21.272	ICD-10 CM	FLEXION DEFORMITY LEFT ANKLE AND TOES
Deformity Compliction	M21.279	ICD-10 CM	FLEXION DEFORMITY UNSPECIFIED ANKLE AND TOES
Deformity Compliction	M21.172	ICD-10 CM	VARUS DEFORMITY NOT ELSEWHERE CLASSIFIED LEFT ANKLE
Deformity Compliction	M21.171	ICD-10 CM	VARUS DEFORMITY NOT ELSEWHERE CLASSIFIED RIGHT ANKLE
Deformity Compliction	M21.071	ICD-10 CM	VALGUS DEFORMITY NOT ELSEWHERE CLASSIFIED RIGHT ANKLE
Deformity Compliction	M21.079	ICD-10 CM	VALGUS DEFORMITY NOT ELSEWHERE CLASSIFIED UNSPECIFIED ANKLE
Deformity Compliction	M21.271	ICD-10 CM	FLEXION DEFORMITY RIGHT ANKLE AND TOES
Deformity Compliction	M21.072	ICD-10 CM	VALGUS DEFORMITY NOT ELSEWHERE CLASSIFIED LEFT ANKLE
Deformity Compliction	M21.179	ICD-10 CM	VARUS DEFORMITY NOT ELSEWHERE CLASSIFIED UNSPECIFIED ANKLE
Delayed Healing	S82.863H	ICD-10 CM	DISPLACED MAISONNEUVE'S FRACTURE OF UNSPECIFIED LEG SUBSEQUENT ENCOUNTER FOR OPEN FRACTURE TYPE I OR II WITH DELAYED HEALING
Delayed Healing	S82.51XH	ICD-10 CM	DISPLACED FRACTURE OF MEDIAL MALLEOLUS OF RIGHT TIBIA SUBSEQUENT ENCOUNTER FOR OPEN FRACTURE TYPE I OR II WITH DELAYED HEALING
Delayed Healing	S82.302G	ICD-10 CM	UNSPECIFIED FRACTURE OF LOWER END OF LEFT TIBIA SUBSEQUENT ENCOUNTER FOR CLOSED FRACTURE WITH DELAYED HEALING
Delayed Healing	S82.52XJ	ICD-10 CM	DISPLACED FRACTURE OF MEDIAL MALLEOLUS OF LEFT TIBIA SUBSEQUENT ENCOUNTER FOR OPEN FRACTURE TYPE IIIA IIIB OR IIIC WITH DELAYED HEALING
Delayed Healing	S82.862G	ICD-10 CM	DISPLACED MAISONNEUVE'S FRACTURE OF LEFT LEG SUBSEQUENT ENCOUNTER FOR CLOSED FRACTURE WITH DELAYED HEALING
Delayed Healing	S82.844J	ICD-10 CM	NONDISPLACED BIMALLEOLAR FRACTURE OF RIGHT LOWER LEG SUBSEQUENT ENCOUNTER FOR OPEN FRACTURE TYPE IIIA IIIB OR IIIC WITH DELAYED HEALING
Delayed Healing	S82.54XG	ICD-10 CM	NONDISPLACED FRACTURE OF MEDIAL MALLEOLUS OF RIGHT TIBIA SUBSEQUENT ENCOUNTER FOR CLOSED FRACTURE WITH DELAYED HEALING
Delayed Healing	S82.876H	ICD-10 CM	NONDISPLACED PILON FRACTURE OF UNSPECIFIED TIBIA SUBSEQUENT ENCOUNTER FOR OPEN FRACTURE TYPE I OR II WITH DELAYED HEALING
Delayed Healing	S82.844H	ICD-10 CM	NONDISPLACED BIMALLEOLAR FRACTURE OF RIGHT LOWER LEG SUBSEQUENT ENCOUNTER FOR OPEN FRACTURE TYPE I OR II WITH DELAYED HEALING
Delayed Healing	S82.863G	ICD-10 CM	DISPLACED MAISONNEUVE'S FRACTURE OF UNSPECIFIED LEG SUBSEQUENT ENCOUNTER FOR CLOSED FRACTURE WITH DELAYED HEALING
Delayed Healing	S82.301G	ICD-10 CM	UNSPECIFIED FRACTURE OF LOWER END OF RIGHT TIBIA SUBSEQUENT ENCOUNTER FOR CLOSED FRACTURE WITH DELAYED HEALING
Delayed Healing	S82.853H	ICD-10 CM	DISPLACED TRIMALLEOLAR FRACTURE OF UNSPECIFIED LOWER LEG SUBSEQUENT ENCOUNTER FOR OPEN FRACTURE TYPE I OR II WITH DELAYED HEALING

Delayed Healing	S82.874J	ICD-10 CM	NONDISPLACED PILON FRACTURE OF RIGHT TIBIA SUBSEQUENT ENCOUNTER FOR OPEN FRACTURE TYPE IIIA IIIB OR IIIC WITH DELAYED HEALING
Delayed Healing	S82.841H	ICD-10 CM	DISPLACED BIMALLEOLAR FRACTURE OF RIGHT LOWER LEG SUBSEQUENT ENCOUNTER FOR OPEN FRACTURE TYPE I OR II WITH DELAYED HEALING
Delayed Healing	S82.874H	ICD-10 CM	NONDISPLACED PILON FRACTURE OF RIGHT TIBIA SUBSEQUENT ENCOUNTER FOR OPEN FRACTURE TYPE I OR II WITH DELAYED HEALING
Delayed Healing	S82.846H	ICD-10 CM	NONDISPLACED BIMALLEOLAR FRACTURE OF UNSPECIFIED LOWER LEG SUBSEQUENT ENCOUNTER FOR OPEN FRACTURE TYPE I OR II WITH DELAYED HEALING
Delayed Healing	S82.311G	ICD-10 CM	TORUS FRACTURE OF LOWER END OF RIGHT TIBIA SUBSEQUENT ENCOUNTER FOR FRACTURE WITH DELAYED HEALING
Delayed Healing	S82.865J	ICD-10 CM	NONDISPLACED MAISONNEUVE'S FRACTURE OF LEFT LEG SUBSEQUENT ENCOUNTER FOR OPEN FRACTURE TYPE IIIA IIIB OR IIIC WITH DELAYED HEALING
Delayed Healing	S82.855J	ICD-10 CM	NONDISPLACED TRIMALLEOLAR FRACTURE OF LEFT LOWER LEG SUBSEQUENT ENCOUNTER FOR OPEN FRACTURE TYPE IIIA IIIB OR IIIC WITH DELAYED HEALING
Delayed Healing	S82.56XJ	ICD-10 CM	NONDISPLACED FRACTURE OF MEDIAL MALLEOLUS OF UNSPECIFIED TIBIA SUBSEQUENT ENCOUNTER FOR OPEN FRACTURE TYPE IIIA IIIB OR IIIC WITH DELAYED HE
Delayed Healing	S82.302J	ICD-10 CM	UNSPECIFIED FRACTURE OF LOWER END OF LEFT TIBIA SUBSEQUENT ENCOUNTER FOR OPEN FRACTURE TYPE IIIA IIIB OR IIIC WITH DELAYED HEALING
Delayed Healing	S82.864J	ICD-10 CM	NONDISPLACED MAISONNEUVE'S FRACTURE OF RIGHT LEG SUBSEQUENT ENCOUNTER FOR OPEN FRACTURE TYPE IIIA IIIB OR IIIC WITH DELAYED HEALING
Delayed Healing	S82.865H	ICD-10 CM	NONDISPLACED MAISONNEUVE'S FRACTURE OF LEFT LEG SUBSEQUENT ENCOUNTER FOR OPEN FRACTURE TYPE I OR II WITH DELAYED HEALING
Delayed Healing	S82.856J	ICD-10 CM	NONDISPLACED TRIMALLEOLAR FRACTURE OF UNSPECIFIED LOWER LEG SUBSEQUENT ENCOUNTER FOR OPEN FRACTURE TYPE IIIA IIIB OR IIIC WITH DELAYED HEALI
Delayed Healing	S82.53XJ	ICD-10 CM	DISPLACED FRACTURE OF MEDIAL MALLEOLUS OF UNSPECIFIED TIBIA SUBSEQUENT ENCOUNTER FOR OPEN FRACTURE TYPE IIIA IIIB OR IIIC WITH DELAYED HEALI
Delayed Healing	S82.62XG	ICD-10 CM	DISPLACED FRACTURE OF LATERAL MALLEOLUS OF LEFT FIBULA SUBSEQUENT ENCOUNTER FOR CLOSED FRACTURE WITH DELAYED HEALING
Delayed Healing	S82.53XH	ICD-10 CM	DISPLACED FRACTURE OF MEDIAL MALLEOLUS OF UNSPECIFIED TIBIA SUBSEQUENT ENCOUNTER FOR OPEN FRACTURE TYPE I OR II WITH DELAYED HEALING
Delayed Healing	S82.55XH	ICD-10 CM	NONDISPLACED FRACTURE OF MEDIAL MALLEOLUS OF LEFT TIBIA SUBSEQUENT ENCOUNTER FOR OPEN FRACTURE TYPE I OR II WITH DELAYED HEALING
Delayed Healing	S82.854H	ICD-10 CM	NONDISPLACED TRIMALLEOLAR FRACTURE OF RIGHT LOWER LEG SUBSEQUENT ENCOUNTER FOR OPEN FRACTURE TYPE I OR II WITH DELAYED HEALING
Delayed Healing	S82.864G	ICD-10 CM	NONDISPLACED MAISONNEUVE'S FRACTURE OF RIGHT LEG SUBSEQUENT ENCOUNTER FOR CLOSED FRACTURE WITH DELAYED HEALING
Delayed Healing	S82.55XJ	ICD-10 CM	NONDISPLACED FRACTURE OF MEDIAL MALLEOLUS OF LEFT TIBIA SUBSEQUENT ENCOUNTER FOR OPEN FRACTURE TYPE IIIA IIIB OR IIIC WITH DELAYED HEALING
Delayed Healing	S82.876G	ICD-10 CM	NONDISPLACED PILON FRACTURE OF UNSPECIFIED TIBIA SUBSEQUENT ENCOUNTER FOR CLOSED FRACTURE WITH DELAYED HEALING
Delayed Healing	S82.56XH	ICD-10 CM	NONDISPLACED FRACTURE OF MEDIAL MALLEOLUS OF UNSPECIFIED TIBIA SUBSEQUENT ENCOUNTER FOR OPEN FRACTURE TYPE I OR II WITH DELAYED HEALING
Delayed Healing	S82.872G	ICD-10 CM	DISPLACED PILON FRACTURE OF LEFT TIBIA SUBSEQUENT ENCOUNTER FOR CLOSED FRACTURE WITH DELAYED HEALING
Delayed Healing	S82.845H	ICD-10 CM	NONDISPLACED BIMALLEOLAR FRACTURE OF LEFT LOWER LEG SUBSEQUENT ENCOUNTER FOR OPEN FRACTURE TYPE I OR II WITH DELAYED HEALING
Delayed Healing	S82.861J	ICD-10 CM	DISPLACED MAISONNEUVE'S FRACTURE OF RIGHT LEG SUBSEQUENT ENCOUNTER FOR OPEN FRACTURE TYPE IIIA IIIB OR IIIC WITH DELAYED HEALING
Delayed Healing	S82.309H	ICD-10 CM	UNSPECIFIED FRACTURE OF LOWER END OF UNSPECIFIED TIBIA SUBSEQUENT ENCOUNTER FOR OPEN FRACTURE TYPE I OR II WITH DELAYED HEALING
Delayed Healing	S82.892J	ICD-10 CM	OTHER FRACTURE OF LEFT LOWER LEG SUBSEQUENT ENCOUNTER FOR OPEN FRACTURE TYPE IIIA IIIB OR IIIC WITH DELAYED HEALING
Delayed Healing	S82.853G	ICD-10 CM	DISPLACED TRIMALLEOLAR FRACTURE OF UNSPECIFIED LOWER LEG SUBSEQUENT ENCOUNTER FOR CLOSED FRACTURE WITH DELAYED HEALING
Delayed Healing	S82.892H	ICD-10 CM	OTHER FRACTURE OF LEFT LOWER LEG SUBSEQUENT ENCOUNTER FOR OPEN FRACTURE TYPE I OR II WITH DELAYED HEALING
Delayed Healing	S82.392G	ICD-10 CM	OTHER FRACTURE OF LOWER END OF LEFT TIBIA SUBSEQUENT ENCOUNTER FOR CLOSED FRACTURE WITH DELAYED HEALING
Delayed Healing	S82.64XH	ICD-10 CM	NONDISPLACED FRACTURE OF LATERAL MALLEOLUS OF RIGHT FIBULA SUBSEQUENT ENCOUNTER FOR OPEN FRACTURE TYPE I OR II WITH DELAYED HEALING
Delayed Healing	S82.63XJ	ICD-10 CM	DISPLACED FRACTURE OF LATERAL MALLEOLUS OF UNSPECIFIED FIBULA SUBSEQUENT ENCOUNTER FOR OPEN FRACTURE TYPE IIIA IIIB OR IIIC WITH DELAYED HEA
Delayed Healing	S82.65XJ	ICD-10 CM	NONDISPLACED FRACTURE OF LATERAL MALLEOLUS OF LEFT FIBULA SUBSEQUENT ENCOUNTER FOR OPEN FRACTURE TYPE IIIA IIIB OR IIIC WITH DELAYED HEALING
Delayed Healing	S82.842H	ICD-10 CM	DISPLACED BIMALLEOLAR FRACTURE OF LEFT LOWER LEG SUBSEQUENT ENCOUNTER FOR OPEN FRACTURE TYPE I OR II WITH DELAYED HEALING
Delayed Healing	S82.854G	ICD-10 CM	NONDISPLACED TRIMALLEOLAR FRACTURE OF RIGHT LOWER LEG SUBSEQUENT ENCOUNTER FOR CLOSED FRACTURE WITH DELAYED HEALING

Delayed Healing	S82.52XH	ICD-10 CM	DISPLACED FRACTURE OF MEDIAL MALLEOLUS OF LEFT TIBIA SUBSEQUENT ENCOUNTER FOR OPEN FRACTURE TYPE I OR II WITH DELAYED HEALING
Delayed Healing	S82.843J	ICD-10 CM	DISPLACED BIMALLEOLAR FRACTURE OF UNSPECIFIED LOWER LEG SUBSEQUENT ENCOUNTER FOR OPEN FRACTURE TYPE IIIA IIIB OR IIIC WITH DELAYED HEALING
Delayed Healing	S82.856G	ICD-10 CM	NONDISPLACED TRIMALLEOLAR FRACTURE OF UNSPECIFIED LOWER LEG SUBSEQUENT ENCOUNTER FOR CLOSED FRACTURE WITH DELAYED HEALING
Delayed Healing	S82.54XH	ICD-10 CM	NONDISPLACED FRACTURE OF MEDIAL MALLEOLUS OF RIGHT TIBIA SUBSEQUENT ENCOUNTER FOR OPEN FRACTURE TYPE I OR II WITH DELAYED HEALING
Delayed Healing	S82.862J	ICD-10 CM	DISPLACED MAISONNEUVE'S FRACTURE OF LEFT LEG SUBSEQUENT ENCOUNTER FOR OPEN FRACTURE TYPE IIIA IIIB OR IIIC WITH DELAYED HEALING
Delayed Healing	S82.841J	ICD-10 CM	DISPLACED BIMALLEOLAR FRACTURE OF RIGHT LOWER LEG SUBSEQUENT ENCOUNTER FOR OPEN FRACTURE TYPE IIIA IIIB OR IIIC WITH DELAYED HEALING
Delayed Healing	S82.856H	ICD-10 CM	NONDISPLACED TRIMALLEOLAR FRACTURE OF UNSPECIFIED LOWER LEG SUBSEQUENT ENCOUNTER FOR OPEN FRACTURE TYPE I OR II WITH DELAYED HEALING
Delayed Healing	S82.842G	ICD-10 CM	DISPLACED BIMALLEOLAR FRACTURE OF LEFT LOWER LEG SUBSEQUENT ENCOUNTER FOR CLOSED FRACTURE WITH DELAYED HEALING
Delayed Healing	S82.863J	ICD-10 CM	DISPLACED MAISONNEUVE'S FRACTURE OF UNSPECIFIED LEG SUBSEQUENT ENCOUNTER FOR OPEN FRACTURE TYPE IIIA IIIB OR IIIC WITH DELAYED HEALING
Delayed Healing	S82.851H	ICD-10 CM	DISPLACED TRIMALLEOLAR FRACTURE OF RIGHT LOWER LEG SUBSEQUENT ENCOUNTER FOR OPEN FRACTURE TYPE I OR II WITH DELAYED HEALING
Delayed Healing	S82.66XG	ICD-10 CM	NONDISPLACED FRACTURE OF LATERAL MALLEOLUS OF UNSPECIFIED FIBULA SUBSEQUENT ENCOUNTER FOR CLOSED FRACTURE WITH DELAYED HEALING
Delayed Healing	S82.61XJ	ICD-10 CM	DISPLACED FRACTURE OF LATERAL MALLEOLUS OF RIGHT FIBULA SUBSEQUENT ENCOUNTER FOR OPEN FRACTURE TYPE IIIA IIIB OR IIIC WITH DELAYED HEALING
Delayed Healing	S82.852J	ICD-10 CM	DISPLACED TRIMALLEOLAR FRACTURE OF LEFT LOWER LEG SUBSEQUENT ENCOUNTER FOR OPEN FRACTURE TYPE IIIA IIIB OR IIIC WITH DELAYED HEALING
Delayed Healing	S82.899G	ICD-10 CM	OTHER FRACTURE OF UNSPECIFIED LOWER LEG SUBSEQUENT ENCOUNTER FOR CLOSED FRACTURE WITH DELAYED HEALING
Delayed Healing	S82.64XG	ICD-10 CM	NONDISPLACED FRACTURE OF LATERAL MALLEOLUS OF RIGHT FIBULA SUBSEQUENT ENCOUNTER FOR CLOSED FRACTURE WITH DELAYED HEALING
Delayed Healing	S82.54XJ	ICD-10 CM	NONDISPLACED FRACTURE OF MEDIAL MALLEOLUS OF RIGHT TIBIA SUBSEQUENT ENCOUNTER FOR OPEN FRACTURE TYPE IIIA IIIB OR IIIC WITH DELAYED HEALING
Delayed Healing	S82.842J	ICD-10 CM	DISPLACED BIMALLEOLAR FRACTURE OF LEFT LOWER LEG SUBSEQUENT ENCOUNTER FOR OPEN FRACTURE TYPE IIIA IIIB OR IIIC WITH DELAYED HEALING
Delayed Healing	S82.846G	ICD-10 CM	NONDISPLACED BIMALLEOLAR FRACTURE OF UNSPECIFIED LOWER LEG SUBSEQUENT ENCOUNTER FOR CLOSED FRACTURE WITH DELAYED HEALING
Delayed Healing	S82.873G	ICD-10 CM	DISPLACED PILON FRACTURE OF UNSPECIFIED TIBIA SUBSEQUENT ENCOUNTER FOR CLOSED FRACTURE WITH DELAYED HEALING
Delayed Healing	S82.63XG	ICD-10 CM	DISPLACED FRACTURE OF LATERAL MALLEOLUS OF UNSPECIFIED FIBULA SUBSEQUENT ENCOUNTER FOR CLOSED FRACTURE WITH DELAYED HEALING
Delayed Healing	S82.309J	ICD-10 CM	UNSPECIFIED FRACTURE OF LOWER END OF UNSPECIFIED TIBIA SUBSEQUENT ENCOUNTER FOR OPEN FRACTURE TYPE IIIA IIIB OR IIIC WITH DELAYED HEALING
Delayed Healing	S82.851J	ICD-10 CM	DISPLACED TRIMALLEOLAR FRACTURE OF RIGHT LOWER LEG SUBSEQUENT ENCOUNTER FOR OPEN FRACTURE TYPE IIIA IIIB OR IIIC WITH DELAYED HEALING
Delayed Healing	S82.866H	ICD-10 CM	NONDISPLACED MAISONNEUVE'S FRACTURE OF UNSPECIFIED LEG SUBSEQUENT ENCOUNTER FOR OPEN FRACTURE TYPE I OR II WITH DELAYED HEALING
Delayed Healing	S82.55XG	ICD-10 CM	NONDISPLACED FRACTURE OF MEDIAL MALLEOLUS OF LEFT TIBIA SUBSEQUENT ENCOUNTER FOR CLOSED FRACTURE WITH DELAYED HEALING
Delayed Healing	S82.301J	ICD-10 CM	UNSPECIFIED FRACTURE OF LOWER END OF RIGHT TIBIA SUBSEQUENT ENCOUNTER FOR OPEN FRACTURE TYPE IIIA IIIB OR IIIC WITH DELAYED HEALING
Delayed Healing	S82.66XH	ICD-10 CM	NONDISPLACED FRACTURE OF LATERAL MALLEOLUS OF UNSPECIFIED FIBULA SUBSEQUENT ENCOUNTER FOR OPEN FRACTURE TYPE I OR II WITH DELAYED HEALING
Delayed Healing	S82.865G	ICD-10 CM	NONDISPLACED MAISONNEUVE'S FRACTURE OF LEFT LEG SUBSEQUENT ENCOUNTER FOR CLOSED FRACTURE WITH DELAYED HEALING
Delayed Healing	S82.62XH	ICD-10 CM	DISPLACED FRACTURE OF LATERAL MALLEOLUS OF LEFT FIBULA SUBSEQUENT ENCOUNTER FOR OPEN FRACTURE TYPE I OR II WITH DELAYED HEALING
Delayed Healing	S82.851G	ICD-10 CM	DISPLACED TRIMALLEOLAR FRACTURE OF RIGHT LOWER LEG SUBSEQUENT ENCOUNTER FOR CLOSED FRACTURE WITH DELAYED HEALING
Delayed Healing	S82.309G	ICD-10 CM	UNSPECIFIED FRACTURE OF LOWER END OF UNSPECIFIED TIBIA SUBSEQUENT ENCOUNTER FOR CLOSED FRACTURE WITH DELAYED HEALING
Delayed Healing	S82.392H	ICD-10 CM	OTHER FRACTURE OF LOWER END OF LEFT TIBIA SUBSEQUENT ENCOUNTER FOR OPEN FRACTURE TYPE I OR II WITH DELAYED HEALING
Delayed Healing	S82.855G	ICD-10 CM	NONDISPLACED TRIMALLEOLAR FRACTURE OF LEFT LOWER LEG SUBSEQUENT ENCOUNTER FOR CLOSED FRACTURE WITH DELAYED HEALING
Delayed Healing	S82.61XH	ICD-10 CM	DISPLACED FRACTURE OF LATERAL MALLEOLUS OF RIGHT FIBULA SUBSEQUENT ENCOUNTER FOR OPEN FRACTURE TYPE I OR II WITH DELAYED HEALING
Delayed Healing	S82.845G	ICD-10 CM	NONDISPLACED BIMALLEOLAR FRACTURE OF LEFT LOWER LEG SUBSEQUENT ENCOUNTER FOR CLOSED FRACTURE WITH DELAYED HEALING

Delayed Healing	S82.392J	ICD-10 CM	OTHER FRACTURE OF LOWER END OF LEFT TIBIA SUBSEQUENT ENCOUNTER FOR OPEN FRACTURE TYPE IIIA IIIB OR IIIC WITH DELAYED HEALING
Delayed Healing	S82.853J	ICD-10 CM	DISPLACED TRIMALLEOLAR FRACTURE OF UNSPECIFIED LOWER LEG SUBSEQUENT ENCOUNTER FOR OPEN FRACTURE TYPE IIIA IIIB OR IIIC WITH DELAYED HEALING
Delayed Healing	S82.843H	ICD-10 CM	DISPLACED BIMALLEOLAR FRACTURE OF UNSPECIFIED LOWER LEG SUBSEQUENT ENCOUNTER FOR OPEN FRACTURE TYPE I OR II WITH DELAYED HEALING
Delayed Healing	S82.399G	ICD-10 CM	OTHER FRACTURE OF LOWER END OF UNSPECIFIED TIBIA SUBSEQUENT ENCOUNTER FOR CLOSED FRACTURE WITH DELAYED HEALING
Delayed Healing	S82.65XH	ICD-10 CM	NONDISPLACED FRACTURE OF LATERAL MALLEOLUS OF LEFT FIBULA SUBSEQUENT ENCOUNTER FOR OPEN FRACTURE TYPE I OR II WITH DELAYED HEALING
Delayed Healing	S82.844G	ICD-10 CM	NONDISPLACED BIMALLEOLAR FRACTURE OF RIGHT LOWER LEG SUBSEQUENT ENCOUNTER FOR CLOSED FRACTURE WITH DELAYED HEALING
Delayed Healing	S82.51XJ	ICD-10 CM	DISPLACED FRACTURE OF MEDIAL MALLEOLUS OF RIGHT TIBIA SUBSEQUENT ENCOUNTER FOR OPEN FRACTURE TYPE IIIA IIIB OR IIIC WITH DELAYED HEALING
Delayed Healing	S82.864H	ICD-10 CM	NONDISPLACED MAISONNEUVE'S FRACTURE OF RIGHT LEG SUBSEQUENT ENCOUNTER FOR OPEN FRACTURE TYPE I OR II WITH DELAYED HEALING
Delayed Healing	S82.53XG	ICD-10 CM	DISPLACED FRACTURE OF MEDIAL MALLEOLUS OF UNSPECIFIED TIBIA SUBSEQUENT ENCOUNTER FOR CLOSED FRACTURE WITH DELAYED HEALING
Delayed Healing	S82.56XG	ICD-10 CM	NONDISPLACED FRACTURE OF MEDIAL MALLEOLUS OF UNSPECIFIED TIBIA SUBSEQUENT ENCOUNTER FOR CLOSED FRACTURE WITH DELAYED HEALING
Delayed Healing	S82.61XG	ICD-10 CM	DISPLACED FRACTURE OF LATERAL MALLEOLUS OF RIGHT FIBULA SUBSEQUENT ENCOUNTER FOR CLOSED FRACTURE WITH DELAYED HEALING
Delayed Healing	S82.866G	ICD-10 CM	NONDISPLACED MAISONNEUVE'S FRACTURE OF UNSPECIFIED LEG SUBSEQUENT ENCOUNTER FOR CLOSED FRACTURE WITH DELAYED HEALING
Delayed Healing	S82.62XJ	ICD-10 CM	DISPLACED FRACTURE OF LATERAL MALLEOLUS OF LEFT FIBULA SUBSEQUENT ENCOUNTER FOR OPEN FRACTURE TYPE IIIA IIIB OR IIIC WITH DELAYED HEALING
Delayed Healing	S82.871G	ICD-10 CM	DISPLACED PILON FRACTURE OF RIGHT TIBIA SUBSEQUENT ENCOUNTER FOR CLOSED FRACTURE WITH DELAYED HEALING
Delayed Healing	S82.391H	ICD-10 CM	OTHER FRACTURE OF LOWER END OF RIGHT TIBIA SUBSEQUENT ENCOUNTER FOR OPEN FRACTURE TYPE I OR II WITH DELAYED HEALING
Delayed Healing	S82.875G	ICD-10 CM	NONDISPLACED PILON FRACTURE OF LEFT TIBIA SUBSEQUENT ENCOUNTER FOR CLOSED FRACTURE WITH DELAYED HEALING
Delayed Healing	S82.862H	ICD-10 CM	DISPLACED MAISONNEUVE'S FRACTURE OF LEFT LEG SUBSEQUENT ENCOUNTER FOR OPEN FRACTURE TYPE I OR II WITH DELAYED HEALING
Delayed Healing	S82.891G	ICD-10 CM	OTHER FRACTURE OF RIGHT LOWER LEG SUBSEQUENT ENCOUNTER FOR CLOSED FRACTURE WITH DELAYED HEALING
Delayed Healing	S82.874G	ICD-10 CM	NONDISPLACED PILON FRACTURE OF RIGHT TIBIA SUBSEQUENT ENCOUNTER FOR CLOSED FRACTURE WITH DELAYED HEALING
Delayed Healing	S82.892G	ICD-10 CM	OTHER FRACTURE OF LEFT LOWER LEG SUBSEQUENT ENCOUNTER FOR CLOSED FRACTURE WITH DELAYED HEALING
Delayed Healing	S82.51XG	ICD-10 CM	DISPLACED FRACTURE OF MEDIAL MALLEOLUS OF RIGHT TIBIA SUBSEQUENT ENCOUNTER FOR CLOSED FRACTURE WITH DELAYED HEALING
Delayed Healing	S82.876J	ICD-10 CM	NONDISPLACED PILON FRACTURE OF UNSPECIFIED TIBIA SUBSEQUENT ENCOUNTER FOR OPEN FRACTURE TYPE IIIA IIIB OR IIIC WITH DELAYED HEALING
Delayed Healing	S82.872H	ICD-10 CM	DISPLACED PILON FRACTURE OF LEFT TIBIA SUBSEQUENT ENCOUNTER FOR OPEN FRACTURE TYPE I OR II WITH DELAYED HEALING
Delayed Healing	S82.866J	ICD-10 CM	NONDISPLACED MAISONNEUVE'S FRACTURE OF UNSPECIFIED LEG SUBSEQUENT ENCOUNTER FOR OPEN FRACTURE TYPE IIIA IIIB OR IIIC WITH DELAYED HEALING
Delayed Healing	S82.855H	ICD-10 CM	NONDISPLACED TRIMALLEOLAR FRACTURE OF LEFT LOWER LEG SUBSEQUENT ENCOUNTER FOR OPEN FRACTURE TYPE I OR II WITH DELAYED HEALING
Delayed Healing	S82.399H	ICD-10 CM	OTHER FRACTURE OF LOWER END OF UNSPECIFIED TIBIA SUBSEQUENT ENCOUNTER FOR OPEN FRACTURE TYPE I OR II WITH DELAYED HEALING
Delayed Healing	S82.852H	ICD-10 CM	DISPLACED TRIMALLEOLAR FRACTURE OF LEFT LOWER LEG SUBSEQUENT ENCOUNTER FOR OPEN FRACTURE TYPE I OR II WITH DELAYED HEALING
Delayed Healing	S82.861H	ICD-10 CM	DISPLACED MAISONNEUVE'S FRACTURE OF RIGHT LEG SUBSEQUENT ENCOUNTER FOR OPEN FRACTURE TYPE I OR II WITH DELAYED HEALING
Delayed Healing	S82.852G	ICD-10 CM	DISPLACED TRIMALLEOLAR FRACTURE OF LEFT LOWER LEG SUBSEQUENT ENCOUNTER FOR CLOSED FRACTURE WITH DELAYED HEALING
Delayed Healing	S82.899H	ICD-10 CM	OTHER FRACTURE OF UNSPECIFIED LOWER LEG SUBSEQUENT ENCOUNTER FOR OPEN FRACTURE TYPE I OR II WITH DELAYED HEALING
Delayed Healing	S82.899J	ICD-10 CM	OTHER FRACTURE OF UNSPECIFIED LOWER LEG SUBSEQUENT ENCOUNTER FOR OPEN FRACTURE TYPE IIIA IIIB OR IIIC WITH DELAYED HEALING
Delayed Healing	S82.872J	ICD-10 CM	DISPLACED PILON FRACTURE OF LEFT TIBIA SUBSEQUENT ENCOUNTER FOR OPEN FRACTURE TYPE IIIA IIIB OR IIIC WITH DELAYED HEALING
Delayed Healing	S82.391G	ICD-10 CM	OTHER FRACTURE OF LOWER END OF RIGHT TIBIA SUBSEQUENT ENCOUNTER FOR CLOSED FRACTURE WITH DELAYED HEALING
Delayed Healing	S82.66XJ	ICD-10 CM	NONDISPLACED FRACTURE OF LATERAL MALLEOLUS OF UNSPECIFIED FIBULA SUBSEQUENT ENCOUNTER FOR OPEN FRACTURE TYPE IIIA IIIB OR IIIC WITH DELAYED

Delayed Healing	S82.301H	ICD-10 CM	UNSPECIFIED FRACTURE OF LOWER END OF RIGHT TIBIA SUBSEQUENT ENCOUNTER FOR OPEN FRACTURE TYPE I OR II WITH DELAYED HEALING
Delayed Healing	S82.875H	ICD-10 CM	NONDISPLACED PILON FRACTURE OF LEFT TIBIA SUBSEQUENT ENCOUNTER FOR OPEN FRACTURE TYPE I OR II WITH DELAYED HEALING
Delayed Healing	S82.391J	ICD-10 CM	OTHER FRACTURE OF LOWER END OF RIGHT TIBIA SUBSEQUENT ENCOUNTER FOR OPEN FRACTURE TYPE IIIA IIIB OR IIIC WITH DELAYED HEALING
Delayed Healing	S82.845J	ICD-10 CM	NONDISPLACED BIMALLEOLAR FRACTURE OF LEFT LOWER LEG SUBSEQUENT ENCOUNTER FOR OPEN FRACTURE TYPE IIIA IIIB OR IIIC WITH DELAYED HEALING
Delayed Healing	S82.873J	ICD-10 CM	DISPLACED PILON FRACTURE OF UNSPECIFIED TIBIA SUBSEQUENT ENCOUNTER FOR OPEN FRACTURE TYPE IIIA IIIB OR IIIC WITH DELAYED HEALING
Delayed Healing	S82.873H	ICD-10 CM	DISPLACED PILON FRACTURE OF UNSPECIFIED TIBIA SUBSEQUENT ENCOUNTER FOR OPEN FRACTURE TYPE I OR II WITH DELAYED HEALING
Delayed Healing	S82.319G	ICD-10 CM	TORUS FRACTURE OF LOWER END OF UNSPECIFIED TIBIA SUBSEQUENT ENCOUNTER FOR FRACTURE WITH DELAYED HEALING
Delayed Healing	S82.861G	ICD-10 CM	DISPLACED MAISONNEUVE'S FRACTURE OF RIGHT LEG SUBSEQUENT ENCOUNTER FOR CLOSED FRACTURE WITH DELAYED HEALING
Delayed Healing	S82.63XH	ICD-10 CM	DISPLACED FRACTURE OF LATERAL MALLEOLUS OF UNSPECIFIED FIBULA SUBSEQUENT ENCOUNTER FOR OPEN FRACTURE TYPE I OR II WITH DELAYED HEALING
Delayed Healing	S82.891H	ICD-10 CM	OTHER FRACTURE OF RIGHT LOWER LEG SUBSEQUENT ENCOUNTER FOR OPEN FRACTURE TYPE I OR II WITH DELAYED HEALING
Delayed Healing	S82.875J	ICD-10 CM	NONDISPLACED PILON FRACTURE OF LEFT TIBIA SUBSEQUENT ENCOUNTER FOR OPEN FRACTURE TYPE IIIA IIIB OR IIIC WITH DELAYED HEALING
Delayed Healing	S82.891J	ICD-10 CM	OTHER FRACTURE OF RIGHT LOWER LEG SUBSEQUENT ENCOUNTER FOR OPEN FRACTURE TYPE IIIA IIIB OR IIIC WITH DELAYED HEALING
Delayed Healing	S82.871J	ICD-10 CM	DISPLACED PILON FRACTURE OF RIGHT TIBIA SUBSEQUENT ENCOUNTER FOR OPEN FRACTURE TYPE IIIA IIIB OR IIIC WITH DELAYED HEALING
Delayed Healing	S82.843G	ICD-10 CM	DISPLACED BIMALLEOLAR FRACTURE OF UNSPECIFIED LOWER LEG SUBSEQUENT ENCOUNTER FOR CLOSED FRACTURE WITH DELAYED HEALING
Delayed Healing	S82.399J	ICD-10 CM	OTHER FRACTURE OF LOWER END OF UNSPECIFIED TIBIA SUBSEQUENT ENCOUNTER FOR OPEN FRACTURE TYPE IIIA IIIB OR IIIC WITH DELAYED HEALING
Delayed Healing	S82.841G	ICD-10 CM	DISPLACED BIMALLEOLAR FRACTURE OF RIGHT LOWER LEG SUBSEQUENT ENCOUNTER FOR CLOSED FRACTURE WITH DELAYED HEALING
Delayed Healing	S82.64XJ	ICD-10 CM	NONDISPLACED FRACTURE OF LATERAL MALLEOLUS OF RIGHT FIBULA SUBSEQUENT ENCOUNTER FOR OPEN FRACTURE TYPE IIIA IIIB OR IIIC WITH DELAYED HEALIN
Delayed Healing	S82.846J	ICD-10 CM	NONDISPLACED BIMALLEOLAR FRACTURE OF UNSPECIFIED LOWER LEG SUBSEQUENT ENCOUNTER FOR OPEN FRACTURE TYPE IIIA IIIB OR IIIC WITH DELAYED HEALIN
Delayed Healing	S82.302H	ICD-10 CM	UNSPECIFIED FRACTURE OF LOWER END OF LEFT TIBIA SUBSEQUENT ENCOUNTER FOR OPEN FRACTURE TYPE I OR II WITH DELAYED HEALING
Delayed Healing	S82.854J	ICD-10 CM	NONDISPLACED TRIMALLEOLAR FRACTURE OF RIGHT LOWER LEG SUBSEQUENT ENCOUNTER FOR OPEN FRACTURE TYPE IIIA IIIB OR IIIC WITH DELAYED HEALING
Delayed Healing	S82.52XG	ICD-10 CM	DISPLACED FRACTURE OF MEDIAL MALLEOLUS OF LEFT TIBIA SUBSEQUENT ENCOUNTER FOR CLOSED FRACTURE WITH DELAYED HEALING
Delayed Healing	S82.312G	ICD-10 CM	TORUS FRACTURE OF LOWER END OF LEFT TIBIA SUBSEQUENT ENCOUNTER FOR FRACTURE WITH DELAYED HEALING
Delayed Healing	S82.65XG	ICD-10 CM	NONDISPLACED FRACTURE OF LATERAL MALLEOLUS OF LEFT FIBULA SUBSEQUENT ENCOUNTER FOR CLOSED FRACTURE WITH DELAYED HEALING
Delayed Healing	S82.871H	ICD-10 CM	DISPLACED PILON FRACTURE OF RIGHT TIBIA SUBSEQUENT ENCOUNTER FOR OPEN FRACTURE TYPE I OR II WITH DELAYED HEALING
Infection	T84.623S	ICD-10 CM	INFECTION AND INFLAMMATORY REACTION DUE TO INTERNAL FIXATION DEVICE OF LEFT TIBIA SEQUELA
Infection	M65.172	ICD-10 CM	OTHER INFECTIVE (TENO)SYNOVITIS LEFT ANKLE AND FOOT
Infection	T81.10XD	ICD-10 CM	POSTPROCEDURAL SHOCK UNSPECIFIED SUBSEQUENT ENCOUNTER
Infection	T81.19XA	ICD-10 CM	OTHER POSTPROCEDURAL SHOCK INITIAL ENCOUNTER
Infection	M86.68	ICD-10 CM	OTHER CHRONIC OSTEOMYELITIS OTHER SITE
Infection	M86.562	ICD-10 CM	OTHER CHRONIC HEMATOGENOUS OSTEOMYELITIS LEFT TIBIA AND FIBULA
Infection	T84.7XXD	ICD-10 CM	INFECTION AND INFLAMMATORY REACTION DUE TO OTHER INTERNAL ORTHOPEDIC PROSTHETIC DEVICES IMPLANTS AND GRAFTS SUBSEQUENT ENCOUNTER
Infection	M65.071	ICD-10 CM	ABSCESS OF TENDON SHEATH RIGHT ANKLE AND FOOT
Infection	M86.362	ICD-10 CM	CHRONIC MULTIFOCAL OSTEOMYELITIS LEFT TIBIA AND FIBULA
Infection	T81.11XS	ICD-10 CM	POSTPROCEDURAL CARDIOGENIC SHOCK SEQUELA
Infection	M00.872	ICD-10 CM	ARTHRITIS DUE TO OTHER BACTERIA LEFT ANKLE AND FOOT
Infection	M86.569	ICD-10 CM	OTHER CHRONIC HEMATOGENOUS OSTEOMYELITIS UNSPECIFIED TIBIA AND FIBULA
Infection	M86.461	ICD-10 CM	CHRONIC OSTEOMYELITIS WITH DRAINING SINUS RIGHT TIBIA AND FIBULA
Infection	T84.625A	ICD-10 CM	INFECTION AND INFLAMMATORY REACTION DUE TO INTERNAL FIXATION DEVICE OF LEFT FIBULA INITIAL ENCOUNTER
Infection	T84.624A	ICD-10 CM	INFECTION AND INFLAMMATORY REACTION DUE TO INTERNAL FIXATION DEVICE OF RIGHT FIBULA INITIAL ENCOUNTER
Infection	M86.169	ICD-10 CM	OTHER ACUTE OSTEOMYELITIS UNSPECIFIED TIBIA AND FIBULA

Infection	T81.32XA	ICD-10 CM	DISRUPTION OF INTERNAL OPERATION (SURGICAL) WOUND NOT ELSEWHERE CLASSIFIED INITIAL ENCOUNTER
Infection	M86.58	ICD-10 CM	OTHER CHRONIC HEMATOGENOUS OSTEOMYELITIS OTHER SITE
Infection	M86.379	ICD-10 CM	CHRONIC MULTIFOCAL OSTEOMYELITIS UNSPECIFIED ANKLE AND FOOT
Infection	T81.32XS	ICD-10 CM	DISRUPTION OF INTERNAL OPERATION (SURGICAL) WOUND NOT ELSEWHERE CLASSIFIED SEQUELA
Infection	T84.622D	ICD-10 CM	INFECTION AND INFLAMMATORY REACTION DUE TO INTERNAL FIXATION DEVICE OF RIGHT TIBIA SUBSEQUENT ENCOUNTER
Infection	M86.48	ICD-10 CM	CHRONIC OSTEOMYELITIS WITH DRAINING SINUS OTHER SITE
Infection	T84.625D	ICD-10 CM	INFECTION AND INFLAMMATORY REACTION DUE TO INTERNAL FIXATION DEVICE OF LEFT FIBULA SUBSEQUENT ENCOUNTER
Infection	M00.272	ICD-10 CM	OTHER STREPTOCOCCAL ARTHRITIS LEFT ANKLE AND FOOT
Infection	T84.629S	ICD-10 CM	INFECTION AND INFLAMMATORY REACTION DUE TO INTERNAL FIXATION DEVICE OF UNSPECIFIED BONE OF LEG SEQUELA
Infection	M86.471	ICD-10 CM	CHRONIC OSTEOMYELITIS WITH DRAINING SINUS RIGHT ANKLE AND FOOT
Infection	M65.179	ICD-10 CM	OTHER INFECTIVE (TENO)SYNOVITIS UNSPECIFIED ANKLE AND FOOT
Infection	T84.624S	ICD-10 CM	INFECTION AND INFLAMMATORY REACTION DUE TO INTERNAL FIXATION DEVICE OF RIGHT FIBULA SEQUELA
Infection	M86.261	ICD-10 CM	SUBACUTE OSTEOMYELITIS RIGHT TIBIA AND FIBULA
Infection	M00.172	ICD-10 CM	PNEUMOCOCCAL ARTHRITIS LEFT ANKLE AND FOOT
Infection	M86.462	ICD-10 CM	CHRONIC OSTEOMYELITIS WITH DRAINING SINUS LEFT TIBIA AND FIBULA
Infection	M86.479	ICD-10 CM	CHRONIC OSTEOMYELITIS WITH DRAINING SINUS UNSPECIFIED ANKLE AND FOOT
Infection	M00.171	ICD-10 CM	PNEUMOCOCCAL ARTHRITIS RIGHT ANKLE AND FOOT
Infection	M86.662	ICD-10 CM	OTHER CHRONIC OSTEOMYELITIS LEFT TIBIA AND FIBULA
Infection	M86.179	ICD-10 CM	OTHER ACUTE OSTEOMYELITIS UNSPECIFIED ANKLE AND FOOT
Infection	M86.171	ICD-10 CM	OTHER ACUTE OSTEOMYELITIS RIGHT ANKLE AND FOOT
Infection	M86.679	ICD-10 CM	OTHER CHRONIC OSTEOMYELITIS UNSPECIFIED ANKLE AND FOOT
Infection	M71.171	ICD-10 CM	OTHER INFECTIVE BURSITIS RIGHT ANKLE AND FOOT
Infection	M86.571	ICD-10 CM	OTHER CHRONIC HEMATOGENOUS OSTEOMYELITIS RIGHT ANKLE AND FOOT
Infection	M86.38	ICD-10 CM	CHRONIC MULTIFOCAL OSTEOMYELITIS OTHER SITE
Infection	T84.629A	ICD-10 CM	INFECTION AND INFLAMMATORY REACTION DUE TO INTERNAL FIXATION DEVICE OF UNSPECIFIED BONE OF LEG INITIAL ENCOUNTER
Infection	T84.7XXA	ICD-10 CM	INFECTION AND INFLAMMATORY REACTION DUE TO OTHER INTERNAL ORTHOPEDIC PROSTHETIC DEVICES IMPLANTS AND GRAFTS INITIAL ENCOUNTER
Infection	M00.179	ICD-10 CM	PNEUMOCOCCAL ARTHRITIS UNSPECIFIED ANKLE AND FOOT
Infection	M86.28	ICD-10 CM	SUBACUTE OSTEOMYELITIS OTHER SITE
Infection	M86.172	ICD-10 CM	OTHER ACUTE OSTEOMYELITIS LEFT ANKLE AND FOOT
Infection	M86.572	ICD-10 CM	OTHER CHRONIC HEMATOGENOUS OSTEOMYELITIS LEFT ANKLE AND FOOT
Infection	M71.172	ICD-10 CM	OTHER INFECTIVE BURSITIS LEFT ANKLE AND FOOT
Infection	M86.269	ICD-10 CM	SUBACUTE OSTEOMYELITIS UNSPECIFIED TIBIA AND FIBULA
Infection	M86.271	ICD-10 CM	SUBACUTE OSTEOMYELITIS RIGHT ANKLE AND FOOT
Infection	T81.33XD	ICD-10 CM	DISRUPTION OF TRAUMATIC INJURY WOUND REPAIR SUBSEQUENT ENCOUNTER
Infection	M00.079	ICD-10 CM	STAPHYLOCOCCAL ARTHRITIS UNSPECIFIED ANKLE AND FOOT
Infection	M65.072	ICD-10 CM	ABSCESS OF TENDON SHEATH LEFT ANKLE AND FOOT
Infection	T84.622A	ICD-10 CM	INFECTION AND INFLAMMATORY REACTION DUE TO INTERNAL FIXATION DEVICE OF RIGHT TIBIA INITIAL ENCOUNTER
Infection	T81.33XS	ICD-10 CM	DISRUPTION OF TRAUMATIC INJURY WOUND REPAIR SEQUELA
Infection	M86.279	ICD-10 CM	SUBACUTE OSTEOMYELITIS UNSPECIFIED ANKLE AND FOOT
Infection	M86.061	ICD-10 CM	ACUTE HEMATOGENOUS OSTEOMYELITIS RIGHT TIBIA AND FIBULA
Infection	M86.672	ICD-10 CM	OTHER CHRONIC OSTEOMYELITIS LEFT ANKLE AND FOOT
Infection	M00.071	ICD-10 CM	STAPHYLOCOCCAL ARTHRITIS RIGHT ANKLE AND FOOT
Infection	M00.879	ICD-10 CM	ARTHRITIS DUE TO OTHER BACTERIA UNSPECIFIED ANKLE AND FOOT
Infection	M00.279	ICD-10 CM	OTHER STREPTOCOCCAL ARTHRITIS UNSPECIFIED ANKLE AND FOOT
Infection	M86.18	ICD-10 CM	OTHER ACUTE OSTEOMYELITIS OTHER SITE
Infection	T81.4XXD	ICD-10 CM	INFECTION FOLLOWING A PROCEDURE SUBSEQUENT ENCOUNTER
Infection	M00.271	ICD-10 CM	OTHER STREPTOCOCCAL ARTHRITIS RIGHT ANKLE AND FOOT
Infection	T81.4XXS	ICD-10 CM	INFECTION FOLLOWING A PROCEDURE SEQUELA
Infection	M86.561	ICD-10 CM	OTHER CHRONIC HEMATOGENOUS OSTEOMYELITIS RIGHT TIBIA AND FIBULA
Infection	T81.32XD	ICD-10 CM	DISRUPTION OF INTERNAL OPERATION (SURGICAL) WOUND NOT ELSEWHERE CLASSIFIED SUBSEQUENT ENCOUNTER
Infection	T81.33XA	ICD-10 CM	DISRUPTION OF TRAUMATIC INJURY WOUND REPAIR INITIAL ENCOUNTER
Infection	M86.161	ICD-10 CM	OTHER ACUTE OSTEOMYELITIS RIGHT TIBIA AND FIBULA
Infection	T81.11XD	ICD-10 CM	POSTPROCEDURAL CARDIOGENIC SHOCK SUBSEQUENT ENCOUNTER
Infection	M86.062	ICD-10 CM	ACUTE HEMATOGENOUS OSTEOMYELITIS LEFT TIBIA AND FIBULA
Infection	M86.469	ICD-10 CM	CHRONIC OSTEOMYELITIS WITH DRAINING SINUS UNSPECIFIED TIBIA AND FIBULA
Infection	T81.4XXA	ICD-10 CM	INFECTION FOLLOWING A PROCEDURE INITIAL ENCOUNTER
Infection	T84.622S	ICD-10 CM	INFECTION AND INFLAMMATORY REACTION DUE TO INTERNAL FIXATION DEVICE OF RIGHT TIBIA SEQUELA

Infection	T84.623A	ICD-10 CM	INFECTION AND INFLAMMATORY REACTION DUE TO INTERNAL FIXATION DEVICE OF LEFT TIBIA INITIAL ENCOUNTER
Infection	M86.372	ICD-10 CM	CHRONIC MULTIFOCAL OSTEOMYELITIS LEFT ANKLE AND FOOT
Infection	M86.069	ICD-10 CM	ACUTE HEMATOGENOUS OSTEOMYELITIS UNSPECIFIED TIBIA AND FIBULA
Infection	T81.30XS	ICD-10 CM	DISRUPTION OF WOUND UNSPECIFIED SEQUELA
Infection	T84.7XXS	ICD-10 CM	INFECTION AND INFLAMMATORY REACTION DUE TO OTHER INTERNAL ORTHOPEDIC PROSTHETIC DEVICES IMPLANTS AND GRAFTS SEQUELA
Infection	M86.472	ICD-10 CM	CHRONIC OSTEOMYELITIS WITH DRAINING SINUS LEFT ANKLE AND FOOT
Infection	T84.625S	ICD-10 CM	INFECTION AND INFLAMMATORY REACTION DUE TO INTERNAL FIXATION DEVICE OF LEFT FIBULA SEQUELA
Infection	T81.31XD	ICD-10 CM	DISRUPTION OF EXTERNAL OPERATION (SURGICAL) WOUND NOT ELSEWHERE CLASSIFIED SUBSEQUENT ENCOUNTER
Infection	M86.661	ICD-10 CM	OTHER CHRONIC OSTEOMYELITIS RIGHT TIBIA AND FIBULA
Infection	M86.669	ICD-10 CM	OTHER CHRONIC OSTEOMYELITIS UNSPECIFIED TIBIA AND FIBULA
Infection	T81.30XD	ICD-10 CM	DISRUPTION OF WOUND UNSPECIFIED SUBSEQUENT ENCOUNTER
Infection	M86.371	ICD-10 CM	CHRONIC MULTIFOCAL OSTEOMYELITIS RIGHT ANKLE AND FOOT
Infection	M65.079	ICD-10 CM	ABSCESS OF TENDON SHEATH UNSPECIFIED ANKLE AND FOOT
Infection	T84.623D	ICD-10 CM	INFECTION AND INFLAMMATORY REACTION DUE TO INTERNAL FIXATION DEVICE OF LEFT TIBIA SUBSEQUENT ENCOUNTER
Infection	M00.871	ICD-10 CM	ARTHRITIS DUE TO OTHER BACTERIA RIGHT ANKLE AND FOOT
Infection	T81.12XA	ICD-10 CM	POSTPROCEDURAL SEPTIC SHOCK INITIAL ENCOUNTER
Infection	T81.12XS	ICD-10 CM	POSTPROCEDURAL SEPTIC SHOCK SEQUELA
Infection	T84.624D	ICD-10 CM	INFECTION AND INFLAMMATORY REACTION DUE TO INTERNAL FIXATION DEVICE OF RIGHT FIBULA SUBSEQUENT ENCOUNTER
Infection	T81.31XA	ICD-10 CM	DISRUPTION OF EXTERNAL OPERATION (SURGICAL) WOUND NOT ELSEWHERE CLASSIFIED INITIAL ENCOUNTER
Infection	M86.08	ICD-10 CM	ACUTE HEMATOGENOUS OSTEOMYELITIS OTHER SITES
Infection	M86.579	ICD-10 CM	OTHER CHRONIC HEMATOGENOUS OSTEOMYELITIS UNSPECIFIED ANKLE AND FOOT
Infection	M86.071	ICD-10 CM	ACUTE HEMATOGENOUS OSTEOMYELITIS RIGHT ANKLE AND FOOT
Infection	T81.11XA	ICD-10 CM	POSTPROCEDURAL CARDIOGENIC SHOCK INITIAL ENCOUNTER
Infection	M86.369	ICD-10 CM	CHRONIC MULTIFOCAL OSTEOMYELITIS UNSPECIFIED TIBIA AND FIBULA
Infection	T81.31XS	ICD-10 CM	DISRUPTION OF EXTERNAL OPERATION (SURGICAL) WOUND NOT ELSEWHERE CLASSIFIED SEQUELA
Infection	T81.19XS	ICD-10 CM	OTHER POSTPROCEDURAL SHOCK SEQUELA
Infection	M86.361	ICD-10 CM	CHRONIC MULTIFOCAL OSTEOMYELITIS RIGHT TIBIA AND FIBULA
Infection	T81.12XD	ICD-10 CM	POSTPROCEDURAL SEPTIC SHOCK SUBSEQUENT ENCOUNTER
Infection	T81.10XS	ICD-10 CM	POSTPROCEDURAL SHOCK UNSPECIFIED SEQUELA
Infection	M71.179	ICD-10 CM	OTHER INFECTIVE BURSITIS UNSPECIFIED ANKLE AND FOOT
Infection	M86.079	ICD-10 CM	ACUTE HEMATOGENOUS OSTEOMYELITIS UNSPECIFIED ANKLE AND FOOT
Infection	M65.171	ICD-10 CM	OTHER INFECTIVE (TENO)SYNOVITIS RIGHT ANKLE AND FOOT
Infection	T81.30XA	ICD-10 CM	DISRUPTION OF WOUND UNSPECIFIED INITIAL ENCOUNTER
Infection	M86.671	ICD-10 CM	OTHER CHRONIC OSTEOMYELITIS RIGHT ANKLE AND FOOT
Infection	M86.162	ICD-10 CM	OTHER ACUTE OSTEOMYELITIS LEFT TIBIA AND FIBULA
Infection	T81.10XA	ICD-10 CM	POSTPROCEDURAL SHOCK UNSPECIFIED INITIAL ENCOUNTER
Infection	M86.272	ICD-10 CM	SUBACUTE OSTEOMYELITIS LEFT ANKLE AND FOOT
Infection	M86.262	ICD-10 CM	SUBACUTE OSTEOMYELITIS LEFT TIBIA AND FIBULA
Infection	M86.072	ICD-10 CM	ACUTE HEMATOGENOUS OSTEOMYELITIS LEFT ANKLE AND FOOT
Infection	T84.629D	ICD-10 CM	INFECTION AND INFLAMMATORY REACTION DUE TO INTERNAL FIXATION DEVICE OF UNSPECIFIED BONE OF LEG SUBSEQUENT ENCOUNTER
Infection	T81.19XD	ICD-10 CM	OTHER POSTPROCEDURAL SHOCK SUBSEQUENT ENCOUNTER
Infection	M00.072	ICD-10 CM	STAPHYLOCOCCAL ARTHRITIS LEFT ANKLE AND FOOT
Instability	M25.271	ICD-10 CM	FLAIL JOINT RIGHT ANKLE AND FOOT
Instability	M25.371	ICD-10 CM	OTHER INSTABILITY RIGHT ANKLE
Instability	M25.279	ICD-10 CM	FLAIL JOINT UNSPECIFIED ANKLE AND FOOT
Instability	M25.272	ICD-10 CM	FLAIL JOINT LEFT ANKLE AND FOOT
Instability	M25.372	ICD-10 CM	OTHER INSTABILITY LEFT ANKLE
Instability	M25.373	ICD-10 CM	OTHER INSTABILITY UNSPECIFIED ANKLE
Joint Derangements	M25.072	ICD-10 CM	HEMARTHROSIS LEFT ANKLE
Joint Derangements	M25.071	ICD-10 CM	HEMARTHROSIS RIGHT ANKLE
Joint Derangements	M24.472	ICD-10 CM	RECURRENT DISLOCATION LEFT ANKLE
Joint Derangements	M24.572	ICD-10 CM	CONTRACTURE LEFT ANKLE
Joint Derangements	M24.371	ICD-10 CM	PATHOLOGICAL DISLOCATION OF RIGHT ANKLE NOT ELSEWHERE CLASSIFIED
Joint Derangements	M24.071	ICD-10 CM	LOOSE BODY IN RIGHT ANKLE
Joint Derangements	M25.472	ICD-10 CM	EFFUSION LEFT ANKLE
Joint Derangements	M25.772	ICD-10 CM	OSTEOPHYTE LEFT ANKLE
Joint Derangements	M25.879	ICD-10 CM	OTHER SPECIFIED JOINT DISORDERS UNSPECIFIED ANKLE AND FOOT
Joint Derangements	M24.073	ICD-10 CM	LOOSE BODY IN UNSPECIFIED ANKLE
Joint Derangements	M25.671	ICD-10 CM	STIFFNESS OF RIGHT ANKLE NOT ELSEWHERE CLASSIFIED
Joint Derangements	M24.471	ICD-10 CM	RECURRENT DISLOCATION RIGHT ANKLE

Joint Derangements	M25.471	ICD-10 CM	EFFUSION RIGHT ANKLE
Joint Derangements	M24.873	ICD-10 CM	OTHER SPECIFIC JOINT DERANGEMENTS OF UNSPECIFIED ANKLE NOT ELSEWHERE CLASSIFIED
Joint Derangements	M25.672	ICD-10 CM	STIFFNESS OF LEFT ANKLE NOT ELSEWHERE CLASSIFIED
Joint Derangements	M24.473	ICD-10 CM	RECURRENT DISLOCATION UNSPECIFIED ANKLE
Joint Derangements	M24.273	ICD-10 CM	DISORDER OF LIGAMENT UNSPECIFIED ANKLE
Joint Derangements	M24.672	ICD-10 CM	ANKYLOSIS LEFT ANKLE
Joint Derangements	M25.773	ICD-10 CM	OSTEOPHYTE UNSPECIFIED ANKLE
Joint Derangements	M24.673	ICD-10 CM	ANKYLOSIS UNSPECIFIED ANKLE
Joint Derangements	M24.372	ICD-10 CM	PATHOLOGICAL DISLOCATION OF LEFT ANKLE NOT ELSEWHERE CLASSIFIED
Joint Derangements	M25.771	ICD-10 CM	OSTEOPHYTE RIGHT ANKLE
Joint Derangements	M25.173	ICD-10 CM	FISTULA UNSPECIFIED ANKLE
Joint Derangements	M25.673	ICD-10 CM	STIFFNESS OF UNSPECIFIED ANKLE NOT ELSEWHERE CLASSIFIED
Joint Derangements	M25.171	ICD-10 CM	FISTULA RIGHT ANKLE
Joint Derangements	M24.171	ICD-10 CM	OTHER ARTICULAR CARTILAGE DISORDERS RIGHT ANKLE
Joint Derangements	M25.872	ICD-10 CM	OTHER SPECIFIED JOINT DISORDERS LEFT ANKLE AND FOOT
Joint Derangements	M24.671	ICD-10 CM	ANKYLOSIS RIGHT ANKLE
Joint Derangements	M24.573	ICD-10 CM	CONTRACTURE UNSPECIFIED ANKLE
Joint Derangements	M24.272	ICD-10 CM	DISORDER OF LIGAMENT LEFT ANKLE
Joint Derangements	M25.172	ICD-10 CM	FISTULA LEFT ANKLE
Joint Derangements	M25.871	ICD-10 CM	OTHER SPECIFIED JOINT DISORDERS RIGHT ANKLE AND FOOT
Joint Derangements	M24.072	ICD-10 CM	LOOSE BODY IN LEFT ANKLE
Joint Derangements	M24.872	ICD-10 CM	OTHER SPECIFIC JOINT DERANGEMENTS OF LEFT ANKLE NOT ELSEWHERE CLASSIFIED
Joint Derangements	M25.073	ICD-10 CM	HEMARTHROSIS UNSPECIFIED ANKLE
Joint Derangements	M24.172	ICD-10 CM	OTHER ARTICULAR CARTILAGE DISORDERS LEFT ANKLE
Joint Derangements	M25.473	ICD-10 CM	EFFUSION UNSPECIFIED ANKLE
Joint Derangements	M24.271	ICD-10 CM	DISORDER OF LIGAMENT RIGHT ANKLE
Joint Derangements	M24.871	ICD-10 CM	OTHER SPECIFIC JOINT DERANGEMENTS OF RIGHT ANKLE NOT ELSEWHERE CLASSIFIED
Joint Derangements	M24.373	ICD-10 CM	PATHOLOGICAL DISLOCATION OF UNSPECIFIED ANKLE NOT ELSEWHERE CLASSIFIED
Joint Derangements	M24.571	ICD-10 CM	CONTRACTURE RIGHT ANKLE
Joint Derangements	M24.173	ICD-10 CM	OTHER ARTICULAR CARTILAGE DISORDERS UNSPECIFIED ANKLE
Malunion	S82.855R	ICD-10 CM	NONDISPLACED TRIMALLEOLAR FRACTURE OF LEFT LOWER LEG SUBSEQUENT ENCOUNTER FOR OPEN FRACTURE TYPE IIIA IIIB OR IIIC WITH MALUNION
Malunion	S82.871P	ICD-10 CM	DISPLACED PILON FRACTURE OF RIGHT TIBIA SUBSEQUENT ENCOUNTER FOR CLOSED FRACTURE WITH MALUNION
Malunion	S82.861R	ICD-10 CM	DISPLACED MAISONNEUVE'S FRACTURE OF RIGHT LEG SUBSEQUENT ENCOUNTER FOR OPEN FRACTURE TYPE IIIA IIIB OR IIIC WITH MALUNION
Malunion	S82.843R	ICD-10 CM	DISPLACED BIMALLEOLAR FRACTURE OF UNSPECIFIED LOWER LEG SUBSEQUENT ENCOUNTER FOR OPEN FRACTURE TYPE IIIA IIIB OR IIIC WITH MALUNION
Malunion	S82.63XP	ICD-10 CM	DISPLACED FRACTURE OF LATERAL MALLEOLUS OF UNSPECIFIED FIBULA SUBSEQUENT ENCOUNTER FOR CLOSED FRACTURE WITH MALUNION
Malunion	S82.872Q	ICD-10 CM	DISPLACED PILON FRACTURE OF LEFT TIBIA SUBSEQUENT ENCOUNTER FOR OPEN FRACTURE TYPE I OR II WITH MALUNION
Malunion	S82.863Q	ICD-10 CM	DISPLACED MAISONNEUVE'S FRACTURE OF UNSPECIFIED LEG SUBSEQUENT ENCOUNTER FOR OPEN FRACTURE TYPE I OR II WITH MALUNION
Malunion	S82.852R	ICD-10 CM	DISPLACED TRIMALLEOLAR FRACTURE OF LEFT LOWER LEG SUBSEQUENT ENCOUNTER FOR OPEN FRACTURE TYPE IIIA IIIB OR IIIC WITH MALUNION
Malunion	S82.862Q	ICD-10 CM	DISPLACED MAISONNEUVE'S FRACTURE OF LEFT LEG SUBSEQUENT ENCOUNTER FOR OPEN FRACTURE TYPE I OR II WITH MALUNION
Malunion	S82.875R	ICD-10 CM	NONDISPLACED PILON FRACTURE OF LEFT TIBIA SUBSEQUENT ENCOUNTER FOR OPEN FRACTURE TYPE IIIA IIIB OR IIIC WITH MALUNION
Malunion	S82.64XR	ICD-10 CM	NONDISPLACED FRACTURE OF LATERAL MALLEOLUS OF RIGHT FIBULA SUBSEQUENT ENCOUNTER FOR OPEN FRACTURE TYPE IIIA IIIB OR IIIC WITH MALUNION
Malunion	S82.865R	ICD-10 CM	NONDISPLACED MAISONNEUVE'S FRACTURE OF LEFT LEG SUBSEQUENT ENCOUNTER FOR OPEN FRACTURE TYPE IIIA IIIB OR IIIC WITH MALUNION
Malunion	S82.853R	ICD-10 CM	DISPLACED TRIMALLEOLAR FRACTURE OF UNSPECIFIED LOWER LEG SUBSEQUENT ENCOUNTER FOR OPEN FRACTURE TYPE IIIA IIIB OR IIIC WITH MALUNION
Malunion	S82.61XR	ICD-10 CM	DISPLACED FRACTURE OF LATERAL MALLEOLUS OF RIGHT FIBULA SUBSEQUENT ENCOUNTER FOR OPEN FRACTURE TYPE IIIA IIIB OR IIIC WITH MALUNION
Malunion	S82.841Q	ICD-10 CM	DISPLACED BIMALLEOLAR FRACTURE OF RIGHT LOWER LEG SUBSEQUENT ENCOUNTER FOR OPEN FRACTURE TYPE I OR II WITH MALUNION
Malunion	S82.856Q	ICD-10 CM	NONDISPLACED TRIMALLEOLAR FRACTURE OF UNSPECIFIED LOWER LEG SUBSEQUENT ENCOUNTER FOR OPEN FRACTURE TYPE I OR II WITH MALUNION
Malunion	S82.861P	ICD-10 CM	DISPLACED MAISONNEUVE'S FRACTURE OF RIGHT LEG SUBSEQUENT ENCOUNTER FOR CLOSED FRACTURE WITH MALUNION
Malunion	S82.841R	ICD-10 CM	DISPLACED BIMALLEOLAR FRACTURE OF RIGHT LOWER LEG SUBSEQUENT ENCOUNTER FOR OPEN FRACTURE TYPE IIIA IIIB OR IIIC WITH MALUNION
Malunion	S82.854Q	ICD-10 CM	NONDISPLACED TRIMALLEOLAR FRACTURE OF RIGHT LOWER LEG SUBSEQUENT ENCOUNTER FOR OPEN FRACTURE TYPE I OR II WITH MALUNION

Malunion	S82.854R	ICD-10 CM	NONDISPLACED TRIMALLEOLAR FRACTURE OF RIGHT LOWER LEG SUBSEQUENT ENCOUNTER FOR OPEN FRACTURE TYPE IIIA IIIB OR IIIC WITH MALUNION
Malunion	S82.899Q	ICD-10 CM	OTHER FRACTURE OF UNSPECIFIED LOWER LEG SUBSEQUENT ENCOUNTER FOR OPEN FRACTURE TYPE I OR II WITH MALUNION
Malunion	S82.874R	ICD-10 CM	NONDISPLACED PILON FRACTURE OF RIGHT TIBIA SUBSEQUENT ENCOUNTER FOR OPEN FRACTURE TYPE IIIA IIIB OR IIIC WITH MALUNION
Malunion	S82.861Q	ICD-10 CM	DISPLACED MAISONNEUVE'S FRACTURE OF RIGHT LEG SUBSEQUENT ENCOUNTER FOR OPEN FRACTURE TYPE I OR II WITH MALUNION
Malunion	S82.311P	ICD-10 CM	TORUS FRACTURE OF LOWER END OF RIGHT TIBIA SUBSEQUENT ENCOUNTER FOR FRACTURE WITH MALUNION
Malunion	S82.55XQ	ICD-10 CM	NONDISPLACED FRACTURE OF MEDIAL MALLEOLUS OF LEFT TIBIA SUBSEQUENT ENCOUNTER FOR OPEN FRACTURE TYPE I OR II WITH MALUNION
Malunion	S82.852Q	ICD-10 CM	DISPLACED TRIMALLEOLAR FRACTURE OF LEFT LOWER LEG SUBSEQUENT ENCOUNTER FOR OPEN FRACTURE TYPE I OR II WITH MALUNION
Malunion	S82.53XP	ICD-10 CM	DISPLACED FRACTURE OF MEDIAL MALLEOLUS OF UNSPECIFIED TIBIA SUBSEQUENT ENCOUNTER FOR CLOSED FRACTURE WITH MALUNION
Malunion	S82.55XR	ICD-10 CM	NONDISPLACED FRACTURE OF MEDIAL MALLEOLUS OF LEFT TIBIA SUBSEQUENT ENCOUNTER FOR OPEN FRACTURE TYPE IIIA IIIB OR IIIC WITH MALUNION
Malunion	S82.866Q	ICD-10 CM	NONDISPLACED MAISONNEUVE'S FRACTURE OF UNSPECIFIED LEG SUBSEQUENT ENCOUNTER FOR OPEN FRACTURE TYPE I OR II WITH MALUNION
Malunion	S82.301P	ICD-10 CM	UNSPECIFIED FRACTURE OF LOWER END OF RIGHT TIBIA SUBSEQUENT ENCOUNTER FOR CLOSED FRACTURE WITH MALUNION
Malunion	S82.56XR	ICD-10 CM	NONDISPLACED FRACTURE OF MEDIAL MALLEOLUS OF UNSPECIFIED TIBIA SUBSEQUENT ENCOUNTER FOR OPEN FRACTURE TYPE IIIA IIIB OR IIIC WITH MALUNION
Malunion	S82.66XR	ICD-10 CM	NONDISPLACED FRACTURE OF LATERAL MALLEOLUS OF UNSPECIFIED FIBULA SUBSEQUENT ENCOUNTER FOR OPEN FRACTURE TYPE IIIA IIIB OR IIIC WITH MALUNION
Malunion	S82.55XP	ICD-10 CM	NONDISPLACED FRACTURE OF MEDIAL MALLEOLUS OF LEFT TIBIA SUBSEQUENT ENCOUNTER FOR CLOSED FRACTURE WITH MALUNION
Malunion	S82.851Q	ICD-10 CM	DISPLACED TRIMALLEOLAR FRACTURE OF RIGHT LOWER LEG SUBSEQUENT ENCOUNTER FOR OPEN FRACTURE TYPE I OR II WITH MALUNION
Malunion	S82.852P	ICD-10 CM	DISPLACED TRIMALLEOLAR FRACTURE OF LEFT LOWER LEG SUBSEQUENT ENCOUNTER FOR CLOSED FRACTURE WITH MALUNION
Malunion	S82.391P	ICD-10 CM	OTHER FRACTURE OF LOWER END OF RIGHT TIBIA SUBSEQUENT ENCOUNTER FOR CLOSED FRACTURE WITH MALUNION
Malunion	S82.62XR	ICD-10 CM	DISPLACED FRACTURE OF LATERAL MALLEOLUS OF LEFT FIBULA SUBSEQUENT ENCOUNTER FOR OPEN FRACTURE TYPE IIIA IIIB OR IIIC WITH MALUNION
Malunion	S82.54XQ	ICD-10 CM	NONDISPLACED FRACTURE OF MEDIAL MALLEOLUS OF RIGHT TIBIA SUBSEQUENT ENCOUNTER FOR OPEN FRACTURE TYPE I OR II WITH MALUNION
Malunion	S82.51XR	ICD-10 CM	DISPLACED FRACTURE OF MEDIAL MALLEOLUS OF RIGHT TIBIA SUBSEQUENT ENCOUNTER FOR OPEN FRACTURE TYPE IIIA IIIB OR IIIC WITH MALUNION
Malunion	S82.51XP	ICD-10 CM	DISPLACED FRACTURE OF MEDIAL MALLEOLUS OF RIGHT TIBIA SUBSEQUENT ENCOUNTER FOR CLOSED FRACTURE WITH MALUNION
Malunion	S82.842Q	ICD-10 CM	DISPLACED BIMALLEOLAR FRACTURE OF LEFT LOWER LEG SUBSEQUENT ENCOUNTER FOR OPEN FRACTURE TYPE I OR II WITH MALUNION
Malunion	S82.65XP	ICD-10 CM	NONDISPLACED FRACTURE OF LATERAL MALLEOLUS OF LEFT FIBULA SUBSEQUENT ENCOUNTER FOR CLOSED FRACTURE WITH MALUNION
Malunion	S82.65XR	ICD-10 CM	NONDISPLACED FRACTURE OF LATERAL MALLEOLUS OF LEFT FIBULA SUBSEQUENT ENCOUNTER FOR OPEN FRACTURE TYPE IIIA IIIB OR IIIC WITH MALUNION
Malunion	S82.399Q	ICD-10 CM	OTHER FRACTURE OF LOWER END OF UNSPECIFIED TIBIA SUBSEQUENT ENCOUNTER FOR OPEN FRACTURE TYPE I OR II WITH MALUNION
Malunion	S82.62XQ	ICD-10 CM	DISPLACED FRACTURE OF LATERAL MALLEOLUS OF LEFT FIBULA SUBSEQUENT ENCOUNTER FOR OPEN FRACTURE TYPE I OR II WITH MALUNION
Malunion	S82.65XQ	ICD-10 CM	NONDISPLACED FRACTURE OF LATERAL MALLEOLUS OF LEFT FIBULA SUBSEQUENT ENCOUNTER FOR OPEN FRACTURE TYPE I OR II WITH MALUNION
Malunion	S82.844Q	ICD-10 CM	NONDISPLACED BIMALLEOLAR FRACTURE OF RIGHT LOWER LEG SUBSEQUENT ENCOUNTER FOR OPEN FRACTURE TYPE I OR II WITH MALUNION
Malunion	S82.862R	ICD-10 CM	DISPLACED MAISONNEUVE'S FRACTURE OF LEFT LEG SUBSEQUENT ENCOUNTER FOR OPEN FRACTURE TYPE IIIA IIIB OR IIIC WITH MALUNION
Malunion	S82.302R	ICD-10 CM	UNSPECIFIED FRACTURE OF LOWER END OF LEFT TIBIA SUBSEQUENT ENCOUNTER FOR OPEN FRACTURE TYPE IIIA IIIB OR IIIC WITH MALUNION
Malunion	S82.872P	ICD-10 CM	DISPLACED PILON FRACTURE OF LEFT TIBIA SUBSEQUENT ENCOUNTER FOR CLOSED FRACTURE WITH MALUNION
Malunion	S82.874P	ICD-10 CM	NONDISPLACED PILON FRACTURE OF RIGHT TIBIA SUBSEQUENT ENCOUNTER FOR CLOSED FRACTURE WITH MALUNION
Malunion	S82.892Q	ICD-10 CM	OTHER FRACTURE OF LEFT LOWER LEG SUBSEQUENT ENCOUNTER FOR OPEN FRACTURE TYPE I OR II WITH MALUNION
Malunion	S82.56XQ	ICD-10 CM	NONDISPLACED FRACTURE OF MEDIAL MALLEOLUS OF UNSPECIFIED TIBIA SUBSEQUENT ENCOUNTER FOR OPEN FRACTURE TYPE I OR II WITH MALUNION

Malunion	S82.873P	ICD-10 CM	DISPLACED PILON FRACTURE OF UNSPECIFIED TIBIA SUBSEQUENT ENCOUNTER FOR CLOSED FRACTURE WITH MALUNION
Malunion	S82.864P	ICD-10 CM	NONDISPLACED MAISONNEUVE'S FRACTURE OF RIGHT LEG SUBSEQUENT ENCOUNTER FOR CLOSED FRACTURE WITH MALUNION
Malunion	S82.899P	ICD-10 CM	OTHER FRACTURE OF UNSPECIFIED LOWER LEG SUBSEQUENT ENCOUNTER FOR CLOSED FRACTURE WITH MALUNION
Malunion	S82.876Q	ICD-10 CM	NONDISPLACED PILON FRACTURE OF UNSPECIFIED TIBIA SUBSEQUENT ENCOUNTER FOR OPEN FRACTURE TYPE I OR II WITH MALUNION
Malunion	S82.863R	ICD-10 CM	DISPLACED MAISONNEUVE'S FRACTURE OF UNSPECIFIED LEG SUBSEQUENT ENCOUNTER FOR OPEN FRACTURE TYPE IIIA IIIB OR IIIC WITH MALUNION
Malunion	S82.846Q	ICD-10 CM	NONDISPLACED BIMALLEOLAR FRACTURE OF UNSPECIFIED LOWER LEG SUBSEQUENT ENCOUNTER FOR OPEN FRACTURE TYPE I OR II WITH MALUNION
Malunion	S82.302Q	ICD-10 CM	UNSPECIFIED FRACTURE OF LOWER END OF LEFT TIBIA SUBSEQUENT ENCOUNTER FOR OPEN FRACTURE TYPE I OR II WITH MALUNION
Malunion	S82.854P	ICD-10 CM	NONDISPLACED TRIMALLEOLAR FRACTURE OF RIGHT LOWER LEG SUBSEQUENT ENCOUNTER FOR CLOSED FRACTURE WITH MALUNION
Malunion	S82.61XP	ICD-10 CM	DISPLACED FRACTURE OF LATERAL MALLEOLUS OF RIGHT FIBULA SUBSEQUENT ENCOUNTER FOR CLOSED FRACTURE WITH MALUNION
Malunion	S82.846P	ICD-10 CM	NONDISPLACED BIMALLEOLAR FRACTURE OF UNSPECIFIED LOWER LEG SUBSEQUENT ENCOUNTER FOR CLOSED FRACTURE WITH MALUNION
Malunion	S82.301R	ICD-10 CM	UNSPECIFIED FRACTURE OF LOWER END OF RIGHT TIBIA SUBSEQUENT ENCOUNTER FOR OPEN FRACTURE TYPE IIIA IIIB OR IIIC WITH MALUNION
Malunion	S82.64XP	ICD-10 CM	NONDISPLACED FRACTURE OF LATERAL MALLEOLUS OF RIGHT FIBULA SUBSEQUENT ENCOUNTER FOR CLOSED FRACTURE WITH MALUNION
Malunion	S82.845P	ICD-10 CM	NONDISPLACED BIMALLEOLAR FRACTURE OF LEFT LOWER LEG SUBSEQUENT ENCOUNTER FOR CLOSED FRACTURE WITH MALUNION
Malunion	S82.855Q	ICD-10 CM	NONDISPLACED TRIMALLEOLAR FRACTURE OF LEFT LOWER LEG SUBSEQUENT ENCOUNTER FOR OPEN FRACTURE TYPE I OR II WITH MALUNION
Malunion	S82.871R	ICD-10 CM	DISPLACED PILON FRACTURE OF RIGHT TIBIA SUBSEQUENT ENCOUNTER FOR OPEN FRACTURE TYPE IIIA IIIB OR IIIC WITH MALUNION
Malunion	S82.873Q	ICD-10 CM	DISPLACED PILON FRACTURE OF UNSPECIFIED TIBIA SUBSEQUENT ENCOUNTER FOR OPEN FRACTURE TYPE I OR II WITH MALUNION
Malunion	S82.866P	ICD-10 CM	NONDISPLACED MAISONNEUVE'S FRACTURE OF UNSPECIFIED LEG SUBSEQUENT ENCOUNTER FOR CLOSED FRACTURE WITH MALUNION
Malunion	S82.875Q	ICD-10 CM	NONDISPLACED PILON FRACTURE OF LEFT TIBIA SUBSEQUENT ENCOUNTER FOR OPEN FRACTURE TYPE I OR II WITH MALUNION
Malunion	S82.843Q	ICD-10 CM	DISPLACED BIMALLEOLAR FRACTURE OF UNSPECIFIED LOWER LEG SUBSEQUENT ENCOUNTER FOR OPEN FRACTURE TYPE I OR II WITH MALUNION
Malunion	S82.309R	ICD-10 CM	UNSPECIFIED FRACTURE OF LOWER END OF UNSPECIFIED TIBIA SUBSEQUENT ENCOUNTER FOR OPEN FRACTURE TYPE IIIA IIIB OR IIIC WITH MALUNION
Malunion	S82.891P	ICD-10 CM	OTHER FRACTURE OF RIGHT LOWER LEG SUBSEQUENT ENCOUNTER FOR CLOSED FRACTURE WITH MALUNION
Malunion	S82.851R	ICD-10 CM	DISPLACED TRIMALLEOLAR FRACTURE OF RIGHT LOWER LEG SUBSEQUENT ENCOUNTER FOR OPEN FRACTURE TYPE IIIA IIIB OR IIIC WITH MALUNION
Malunion	S82.309P	ICD-10 CM	UNSPECIFIED FRACTURE OF LOWER END OF UNSPECIFIED TIBIA SUBSEQUENT ENCOUNTER FOR CLOSED FRACTURE WITH MALUNION
Malunion	S82.891Q	ICD-10 CM	OTHER FRACTURE OF RIGHT LOWER LEG SUBSEQUENT ENCOUNTER FOR OPEN FRACTURE TYPE I OR II WITH MALUNION
Malunion	S82.842R	ICD-10 CM	DISPLACED BIMALLEOLAR FRACTURE OF LEFT LOWER LEG SUBSEQUENT ENCOUNTER FOR OPEN FRACTURE TYPE IIIA IIIB OR IIIC WITH MALUNION
Malunion	S82.844R	ICD-10 CM	NONDISPLACED BIMALLEOLAR FRACTURE OF RIGHT LOWER LEG SUBSEQUENT ENCOUNTER FOR OPEN FRACTURE TYPE IIIA IIIB OR IIIC WITH MALUNION
Malunion	S82.54XP	ICD-10 CM	NONDISPLACED FRACTURE OF MEDIAL MALLEOLUS OF RIGHT TIBIA SUBSEQUENT ENCOUNTER FOR CLOSED FRACTURE WITH MALUNION
Malunion	S82.865Q	ICD-10 CM	NONDISPLACED MAISONNEUVE'S FRACTURE OF LEFT LEG SUBSEQUENT ENCOUNTER FOR OPEN FRACTURE TYPE I OR II WITH MALUNION
Malunion	S82.63XR	ICD-10 CM	DISPLACED FRACTURE OF LATERAL MALLEOLUS OF UNSPECIFIED FIBULA SUBSEQUENT ENCOUNTER FOR OPEN FRACTURE TYPE IIIA IIIB OR IIIC WITH MALUNION
Malunion	S82.899R	ICD-10 CM	OTHER FRACTURE OF UNSPECIFIED LOWER LEG SUBSEQUENT ENCOUNTER FOR OPEN FRACTURE TYPE IIIA IIIB OR IIIC WITH MALUNION
Malunion	S82.865P	ICD-10 CM	NONDISPLACED MAISONNEUVE'S FRACTURE OF LEFT LEG SUBSEQUENT ENCOUNTER FOR CLOSED FRACTURE WITH MALUNION
Malunion	S82.319P	ICD-10 CM	TORUS FRACTURE OF LOWER END OF UNSPECIFIED TIBIA SUBSEQUENT ENCOUNTER FOR FRACTURE WITH MALUNION
Malunion	S82.52XR	ICD-10 CM	DISPLACED FRACTURE OF MEDIAL MALLEOLUS OF LEFT TIBIA SUBSEQUENT ENCOUNTER FOR OPEN FRACTURE TYPE IIIA IIIB OR IIIC WITH MALUNION
Malunion	S82.866R	ICD-10 CM	NONDISPLACED MAISONNEUVE'S FRACTURE OF UNSPECIFIED LEG SUBSEQUENT ENCOUNTER FOR OPEN FRACTURE TYPE IIIA IIIB OR IIIC WITH MALUNION

Malunion	S82.392Q	ICD-10 CM	OTHER FRACTURE OF LOWER END OF LEFT TIBIA SUBSEQUENT ENCOUNTER FOR OPEN FRACTURE TYPE I OR II WITH MALUNION
Malunion	S82.845Q	ICD-10 CM	NONDISPLACED BIMALLEOLAR FRACTURE OF LEFT LOWER LEG SUBSEQUENT ENCOUNTER FOR OPEN FRACTURE TYPE I OR II WITH MALUNION
Malunion	S82.309Q	ICD-10 CM	UNSPECIFIED FRACTURE OF LOWER END OF UNSPECIFIED TIBIA SUBSEQUENT ENCOUNTER FOR OPEN FRACTURE TYPE I OR II WITH MALUNION
Malunion	S82.53XQ	ICD-10 CM	DISPLACED FRACTURE OF MEDIAL MALLEOLUS OF UNSPECIFIED TIBIA SUBSEQUENT ENCOUNTER FOR OPEN FRACTURE TYPE I OR II WITH MALUNION
Malunion	S82.392P	ICD-10 CM	OTHER FRACTURE OF LOWER END OF LEFT TIBIA SUBSEQUENT ENCOUNTER FOR CLOSED FRACTURE WITH MALUNION
Malunion	S82.874Q	ICD-10 CM	NONDISPLACED PILON FRACTURE OF RIGHT TIBIA SUBSEQUENT ENCOUNTER FOR OPEN FRACTURE TYPE I OR II WITH MALUNION
Malunion	S82.312P	ICD-10 CM	TORUS FRACTURE OF LOWER END OF LEFT TIBIA SUBSEQUENT ENCOUNTER FOR FRACTURE WITH MALUNION
Malunion	S82.392R	ICD-10 CM	OTHER FRACTURE OF LOWER END OF LEFT TIBIA SUBSEQUENT ENCOUNTER FOR OPEN FRACTURE TYPE IIIA IIIB OR IIIC WITH MALUNION
Malunion	S82.53XR	ICD-10 CM	DISPLACED FRACTURE OF MEDIAL MALLEOLUS OF UNSPECIFIED TIBIA SUBSEQUENT ENCOUNTER FOR OPEN FRACTURE TYPE IIIA IIIB OR IIIC WITH MALUNION
Malunion	S82.52XQ	ICD-10 CM	DISPLACED FRACTURE OF MEDIAL MALLEOLUS OF LEFT TIBIA SUBSEQUENT ENCOUNTER FOR OPEN FRACTURE TYPE I OR II WITH MALUNION
Malunion	S82.301Q	ICD-10 CM	UNSPECIFIED FRACTURE OF LOWER END OF RIGHT TIBIA SUBSEQUENT ENCOUNTER FOR OPEN FRACTURE TYPE I OR II WITH MALUNION
Malunion	S82.851P	ICD-10 CM	DISPLACED TRIMALLEOLAR FRACTURE OF RIGHT LOWER LEG SUBSEQUENT ENCOUNTER FOR CLOSED FRACTURE WITH MALUNION
Malunion	S82.876P	ICD-10 CM	NONDISPLACED PILON FRACTURE OF UNSPECIFIED TIBIA SUBSEQUENT ENCOUNTER FOR CLOSED FRACTURE WITH MALUNION
Malunion	S82.891R	ICD-10 CM	OTHER FRACTURE OF RIGHT LOWER LEG SUBSEQUENT ENCOUNTER FOR OPEN FRACTURE TYPE IIIA IIIB OR IIIC WITH MALUNION
Malunion	S82.856P	ICD-10 CM	NONDISPLACED TRIMALLEOLAR FRACTURE OF UNSPECIFIED LOWER LEG SUBSEQUENT ENCOUNTER FOR CLOSED FRACTURE WITH MALUNION
Malunion	S82.892P	ICD-10 CM	OTHER FRACTURE OF LEFT LOWER LEG SUBSEQUENT ENCOUNTER FOR CLOSED FRACTURE WITH MALUNION
Malunion	S82.841P	ICD-10 CM	DISPLACED BIMALLEOLAR FRACTURE OF RIGHT LOWER LEG SUBSEQUENT ENCOUNTER FOR CLOSED FRACTURE WITH MALUNION
Malunion	S82.872R	ICD-10 CM	DISPLACED PILON FRACTURE OF LEFT TIBIA SUBSEQUENT ENCOUNTER FOR OPEN FRACTURE TYPE IIIA IIIB OR IIIC WITH MALUNION
Malunion	S82.66XP	ICD-10 CM	NONDISPLACED FRACTURE OF LATERAL MALLEOLUS OF UNSPECIFIED FIBULA SUBSEQUENT ENCOUNTER FOR CLOSED FRACTURE WITH MALUNION
Malunion	S82.873R	ICD-10 CM	DISPLACED PILON FRACTURE OF UNSPECIFIED TIBIA SUBSEQUENT ENCOUNTER FOR OPEN FRACTURE TYPE IIIA IIIB OR IIIC WITH MALUNION
Malunion	S82.66XQ	ICD-10 CM	NONDISPLACED FRACTURE OF LATERAL MALLEOLUS OF UNSPECIFIED FIBULA SUBSEQUENT ENCOUNTER FOR OPEN FRACTURE TYPE I OR II WITH MALUNION
Malunion	S82.391Q	ICD-10 CM	OTHER FRACTURE OF LOWER END OF RIGHT TIBIA SUBSEQUENT ENCOUNTER FOR OPEN FRACTURE TYPE I OR II WITH MALUNION
Malunion	S82.62XP	ICD-10 CM	DISPLACED FRACTURE OF LATERAL MALLEOLUS OF LEFT FIBULA SUBSEQUENT ENCOUNTER FOR CLOSED FRACTURE WITH MALUNION
Malunion	S82.399R	ICD-10 CM	OTHER FRACTURE OF LOWER END OF UNSPECIFIED TIBIA SUBSEQUENT ENCOUNTER FOR OPEN FRACTURE TYPE IIIA IIIB OR IIIC WITH MALUNION
Malunion	S82.855P	ICD-10 CM	NONDISPLACED TRIMALLEOLAR FRACTURE OF LEFT LOWER LEG SUBSEQUENT ENCOUNTER FOR CLOSED FRACTURE WITH MALUNION
Malunion	S82.63XQ	ICD-10 CM	DISPLACED FRACTURE OF LATERAL MALLEOLUS OF UNSPECIFIED FIBULA SUBSEQUENT ENCOUNTER FOR OPEN FRACTURE TYPE I OR II WITH MALUNION
Malunion	S82.842P	ICD-10 CM	DISPLACED BIMALLEOLAR FRACTURE OF LEFT LOWER LEG SUBSEQUENT ENCOUNTER FOR CLOSED FRACTURE WITH MALUNION
Malunion	S82.399P	ICD-10 CM	OTHER FRACTURE OF LOWER END OF UNSPECIFIED TIBIA SUBSEQUENT ENCOUNTER FOR CLOSED FRACTURE WITH MALUNION
Malunion	S82.844P	ICD-10 CM	NONDISPLACED BIMALLEOLAR FRACTURE OF RIGHT LOWER LEG SUBSEQUENT ENCOUNTER FOR CLOSED FRACTURE WITH MALUNION
Malunion	S82.871Q	ICD-10 CM	DISPLACED PILON FRACTURE OF RIGHT TIBIA SUBSEQUENT ENCOUNTER FOR OPEN FRACTURE TYPE I OR II WITH MALUNION
Malunion	S82.862P	ICD-10 CM	DISPLACED MAISONNEUVE'S FRACTURE OF LEFT LEG SUBSEQUENT ENCOUNTER FOR CLOSED FRACTURE WITH MALUNION
Malunion	S82.846R	ICD-10 CM	NONDISPLACED BIMALLEOLAR FRACTURE OF UNSPECIFIED LOWER LEG SUBSEQUENT ENCOUNTER FOR OPEN FRACTURE TYPE IIIA IIIB OR IIIC WITH MALUNION
Malunion	S82.876R	ICD-10 CM	NONDISPLACED PILON FRACTURE OF UNSPECIFIED TIBIA SUBSEQUENT ENCOUNTER FOR OPEN FRACTURE TYPE IIIA IIIB OR IIIC WITH MALUNION
Malunion	S82.61XQ	ICD-10 CM	DISPLACED FRACTURE OF LATERAL MALLEOLUS OF RIGHT FIBULA SUBSEQUENT ENCOUNTER FOR OPEN FRACTURE TYPE I OR II WITH MALUNION

Malunion	S82.864Q	ICD-10 CM	NONDISPLACED MAISONNEUVE'S FRACTURE OF RIGHT LEG SUBSEQUENT ENCOUNTER FOR OPEN FRACTURE TYPE I OR II WITH MALUNION
Malunion	S82.391R	ICD-10 CM	OTHER FRACTURE OF LOWER END OF RIGHT TIBIA SUBSEQUENT ENCOUNTER FOR OPEN FRACTURE TYPE IIIA IIIB OR IIIC WITH MALUNION
Malunion	S82.56XP	ICD-10 CM	NONDISPLACED FRACTURE OF MEDIAL MALLEOLUS OF UNSPECIFIED TIBIA SUBSEQUENT ENCOUNTER FOR CLOSED FRACTURE WITH MALUNION
Malunion	S82.302P	ICD-10 CM	UNSPECIFIED FRACTURE OF LOWER END OF LEFT TIBIA SUBSEQUENT ENCOUNTER FOR CLOSED FRACTURE WITH MALUNION
Malunion	S82.853Q	ICD-10 CM	DISPLACED TRIMALLEOLAR FRACTURE OF UNSPECIFIED LOWER LEG SUBSEQUENT ENCOUNTER FOR OPEN FRACTURE TYPE I OR II WITH MALUNION
Malunion	S82.845R	ICD-10 CM	NONDISPLACED BIMALLEOLAR FRACTURE OF LEFT LOWER LEG SUBSEQUENT ENCOUNTER FOR OPEN FRACTURE TYPE IIIA IIIB OR IIIC WITH MALUNION
Malunion	S82.853P	ICD-10 CM	DISPLACED TRIMALLEOLAR FRACTURE OF UNSPECIFIED LOWER LEG SUBSEQUENT ENCOUNTER FOR CLOSED FRACTURE WITH MALUNION
Malunion	S82.863P	ICD-10 CM	DISPLACED MAISONNEUVE'S FRACTURE OF UNSPECIFIED LEG SUBSEQUENT ENCOUNTER FOR CLOSED FRACTURE WITH MALUNION
Malunion	S82.52XP	ICD-10 CM	DISPLACED FRACTURE OF MEDIAL MALLEOLUS OF LEFT TIBIA SUBSEQUENT ENCOUNTER FOR CLOSED FRACTURE WITH MALUNION
Malunion	S82.54XR	ICD-10 CM	NONDISPLACED FRACTURE OF MEDIAL MALLEOLUS OF RIGHT TIBIA SUBSEQUENT ENCOUNTER FOR OPEN FRACTURE TYPE IIIA IIIB OR IIIC WITH MALUNION
Malunion	S82.843P	ICD-10 CM	DISPLACED BIMALLEOLAR FRACTURE OF UNSPECIFIED LOWER LEG SUBSEQUENT ENCOUNTER FOR CLOSED FRACTURE WITH MALUNION
Malunion	S82.864R	ICD-10 CM	NONDISPLACED MAISONNEUVE'S FRACTURE OF RIGHT LEG SUBSEQUENT ENCOUNTER FOR OPEN FRACTURE TYPE IIIA IIIB OR IIIC WITH MALUNION
Malunion	S82.875P	ICD-10 CM	NONDISPLACED PILON FRACTURE OF LEFT TIBIA SUBSEQUENT ENCOUNTER FOR CLOSED FRACTURE WITH MALUNION
Malunion	S82.892R	ICD-10 CM	OTHER FRACTURE OF LEFT LOWER LEG SUBSEQUENT ENCOUNTER FOR OPEN FRACTURE TYPE IIIA IIIB OR IIIC WITH MALUNION
Malunion	S82.856R	ICD-10 CM	NONDISPLACED TRIMALLEOLAR FRACTURE OF UNSPECIFIED LOWER LEG SUBSEQUENT ENCOUNTER FOR OPEN FRACTURE TYPE IIIA IIIB OR IIIC WITH MALUNION
Malunion	S82.64XQ	ICD-10 CM	NONDISPLACED FRACTURE OF LATERAL MALLEOLUS OF RIGHT FIBULA SUBSEQUENT ENCOUNTER FOR OPEN FRACTURE TYPE I OR II WITH MALUNION
Malunion	S82.51XQ	ICD-10 CM	DISPLACED FRACTURE OF MEDIAL MALLEOLUS OF RIGHT TIBIA SUBSEQUENT ENCOUNTER FOR OPEN FRACTURE TYPE I OR II WITH MALUNION
Mechanical Complication	T84.298D	ICD-10 CM	OTHER MECHANICAL COMPLICATION OF INTERNAL FIXATION DEVICE OF OTHER BONES SUBSEQUENT ENCOUNTER
Mechanical Complication	T84.116D	ICD-10 CM	BREAKDOWN (MECHANICAL) OF INTERNAL FIXATION DEVICE OF BONE OF RIGHT LOWER LEG SUBSEQUENT ENCOUNTER
Mechanical Complication	T84.126D	ICD-10 CM	DISPLACEMENT OF INTERNAL FIXATION DEVICE OF BONE OF RIGHT LOWER LEG SUBSEQUENT ENCOUNTER
Mechanical Complication	T84.398D	ICD-10 CM	OTHER MECHANICAL COMPLICATION OF OTHER BONE DEVICES IMPLANTS AND GRAFTS SUBSEQUENT ENCOUNTER
Mechanical Complication	T84.398A	ICD-10 CM	OTHER MECHANICAL COMPLICATION OF OTHER BONE DEVICES IMPLANTS AND GRAFTS INITIAL ENCOUNTER
Mechanical Complication	T84.213A	ICD-10 CM	BREAKDOWN (MECHANICAL) OF INTERNAL FIXATION DEVICE OF BONES OF FOOT AND TOES INITIAL ENCOUNTER
Mechanical Complication	T84.127A	ICD-10 CM	DISPLACEMENT OF INTERNAL FIXATION DEVICE OF BONE OF LEFT LOWER LEG INITIAL ENCOUNTER
Mechanical Complication	T84.117D	ICD-10 CM	BREAKDOWN (MECHANICAL) OF INTERNAL FIXATION DEVICE OF BONE OF LEFT LOWER LEG SUBSEQUENT ENCOUNTER
Mechanical Complication	T84.218S	ICD-10 CM	BREAKDOWN (MECHANICAL) OF INTERNAL FIXATION DEVICE OF OTHER BONES SEQUELA
Mechanical Complication	T84.228D	ICD-10 CM	DISPLACEMENT OF INTERNAL FIXATION DEVICE OF OTHER BONES SUBSEQUENT ENCOUNTER
Mechanical Complication	T84.328S	ICD-10 CM	DISPLACEMENT OF OTHER BONE DEVICES IMPLANTS AND GRAFTS SEQUELA
Mechanical Complication	T84.418A	ICD-10 CM	BREAKDOWN (MECHANICAL) OF OTHER INTERNAL ORTHOPEDIC DEVICES IMPLANTS AND GRAFTS INITIAL ENCOUNTER
Mechanical Complication	T84.418D	ICD-10 CM	BREAKDOWN (MECHANICAL) OF OTHER INTERNAL ORTHOPEDIC DEVICES IMPLANTS AND GRAFTS SUBSEQUENT ENCOUNTER
Mechanical Complication	T84.293S	ICD-10 CM	OTHER MECHANICAL COMPLICATION OF INTERNAL FIXATION DEVICE OF BONES OF FOOT AND TOES SEQUELA
Mechanical Complication	T84.428S	ICD-10 CM	DISPLACEMENT OF OTHER INTERNAL ORTHOPEDIC DEVICES IMPLANTS AND GRAFTS SEQUELA
Mechanical Complication	T84.418S	ICD-10 CM	BREAKDOWN (MECHANICAL) OF OTHER INTERNAL ORTHOPEDIC DEVICES IMPLANTS AND GRAFTS SEQUELA
Mechanical Complication	T84.298S	ICD-10 CM	OTHER MECHANICAL COMPLICATION OF INTERNAL FIXATION DEVICE OF OTHER BONES SEQUELA
Mechanical Complication	T84.223S	ICD-10 CM	DISPLACEMENT OF INTERNAL FIXATION DEVICE OF BONES OF FOOT AND TOES SEQUELA
Mechanical Complication	T84.228S	ICD-10 CM	DISPLACEMENT OF INTERNAL FIXATION DEVICE OF OTHER BONES SEQUELA
Mechanical Complication	T84.223A	ICD-10 CM	DISPLACEMENT OF INTERNAL FIXATION DEVICE OF BONES OF FOOT AND TOES INITIAL ENCOUNTER

Mechanical Complication	T84.117S	ICD-10 CM	BREAKDOWN (MECHANICAL) OF INTERNAL FIXATION DEVICE OF BONE OF LEFT LOWER LEG SEQUELA
Mechanical Complication	T84.126A	ICD-10 CM	DISPLACEMENT OF INTERNAL FIXATION DEVICE OF BONE OF RIGHT LOWER LEG INITIAL ENCOUNTER
Mechanical Complication	T84.218D	ICD-10 CM	BREAKDOWN (MECHANICAL) OF INTERNAL FIXATION DEVICE OF OTHER BONES SUBSEQUENT ENCOUNTER
Mechanical Complication	T84.293A	ICD-10 CM	OTHER MECHANICAL COMPLICATION OF INTERNAL FIXATION DEVICE OF BONES OF FOOT AND TOES INITIAL ENCOUNTER
Mechanical Complication	T84.196D	ICD-10 CM	OTHER MECHANICAL COMPLICATION OF INTERNAL FIXATION DEVICE OF BONE OF RIGHT LOWER LEG SUBSEQUENT ENCOUNTER
Mechanical Complication	T84.293D	ICD-10 CM	OTHER MECHANICAL COMPLICATION OF INTERNAL FIXATION DEVICE OF BONES OF FOOT AND TOES SUBSEQUENT ENCOUNTER
Mechanical Complication	T84.196S	ICD-10 CM	OTHER MECHANICAL COMPLICATION OF INTERNAL FIXATION DEVICE OF BONE OF RIGHT LOWER LEG SEQUELA
Mechanical Complication	T84.428A	ICD-10 CM	DISPLACEMENT OF OTHER INTERNAL ORTHOPEDIC DEVICES IMPLANTS AND GRAFTS INITIAL ENCOUNTER
Mechanical Complication	T84.328A	ICD-10 CM	DISPLACEMENT OF OTHER BONE DEVICES IMPLANTS AND GRAFTS INITIAL ENCOUNTER
Mechanical Complication	T84.318D	ICD-10 CM	BREAKDOWN (MECHANICAL) OF OTHER BONE DEVICES IMPLANTS AND GRAFTS SUBSEQUENT ENCOUNTER
Mechanical Complication	T84.196A	ICD-10 CM	OTHER MECHANICAL COMPLICATION OF INTERNAL FIXATION DEVICE OF BONE OF RIGHT LOWER LEG INITIAL ENCOUNTER
Mechanical Complication	T84.116A	ICD-10 CM	BREAKDOWN (MECHANICAL) OF INTERNAL FIXATION DEVICE OF BONE OF RIGHT LOWER LEG INITIAL ENCOUNTER
Mechanical Complication	T84.228A	ICD-10 CM	DISPLACEMENT OF INTERNAL FIXATION DEVICE OF OTHER BONES INITIAL ENCOUNTER
Mechanical Complication	T84.127D	ICD-10 CM	DISPLACEMENT OF INTERNAL FIXATION DEVICE OF BONE OF LEFT LOWER LEG SUBSEQUENT ENCOUNTER
Mechanical Complication	T84.298A	ICD-10 CM	OTHER MECHANICAL COMPLICATION OF INTERNAL FIXATION DEVICE OF OTHER BONES INITIAL ENCOUNTER
Mechanical Complication	T84.223D	ICD-10 CM	DISPLACEMENT OF INTERNAL FIXATION DEVICE OF BONES OF FOOT AND TOES SUBSEQUENT ENCOUNTER
Mechanical Complication	T84.498S	ICD-10 CM	OTHER MECHANICAL COMPLICATION OF OTHER INTERNAL ORTHOPEDIC DEVICES IMPLANTS AND GRAFTS SEQUELA
Mechanical Complication	T84.218A	ICD-10 CM	BREAKDOWN (MECHANICAL) OF INTERNAL FIXATION DEVICE OF OTHER BONES INITIAL ENCOUNTER
Mechanical Complication	T84.498A	ICD-10 CM	OTHER MECHANICAL COMPLICATION OF OTHER INTERNAL ORTHOPEDIC DEVICES IMPLANTS AND GRAFTS INITIAL ENCOUNTER
Mechanical Complication	T84.197D	ICD-10 CM	OTHER MECHANICAL COMPLICATION OF INTERNAL FIXATION DEVICE OF BONE OF LEFT LOWER LEG SUBSEQUENT ENCOUNTER
Mechanical Complication	T84.116S	ICD-10 CM	BREAKDOWN (MECHANICAL) OF INTERNAL FIXATION DEVICE OF BONE OF RIGHT LOWER LEG SEQUELA
Mechanical Complication	T84.328D	ICD-10 CM	DISPLACEMENT OF OTHER BONE DEVICES IMPLANTS AND GRAFTS SUBSEQUENT ENCOUNTER
Mechanical Complication	T84.428D	ICD-10 CM	DISPLACEMENT OF OTHER INTERNAL ORTHOPEDIC DEVICES IMPLANTS AND GRAFTS SUBSEQUENT ENCOUNTER
Mechanical Complication	T84.318A	ICD-10 CM	BREAKDOWN (MECHANICAL) OF OTHER BONE DEVICES IMPLANTS AND GRAFTS INITIAL ENCOUNTER
Mechanical Complication	T84.213S	ICD-10 CM	BREAKDOWN (MECHANICAL) OF INTERNAL FIXATION DEVICE OF BONES OF FOOT AND TOES SEQUELA
Mechanical Complication	T84.126S	ICD-10 CM	DISPLACEMENT OF INTERNAL FIXATION DEVICE OF BONE OF RIGHT LOWER LEG SEQUELA
Mechanical Complication	T84.197A	ICD-10 CM	OTHER MECHANICAL COMPLICATION OF INTERNAL FIXATION DEVICE OF BONE OF LEFT LOWER LEG INITIAL ENCOUNTER
Mechanical Complication	T84.127S	ICD-10 CM	DISPLACEMENT OF INTERNAL FIXATION DEVICE OF BONE OF LEFT LOWER LEG SEQUELA
Mechanical Complication	T84.398S	ICD-10 CM	OTHER MECHANICAL COMPLICATION OF OTHER BONE DEVICES IMPLANTS AND GRAFTS SEQUELA
Mechanical Complication	T84.197S	ICD-10 CM	OTHER MECHANICAL COMPLICATION OF INTERNAL FIXATION DEVICE OF BONE OF LEFT LOWER LEG SEQUELA
Mechanical Complication	T84.498D	ICD-10 CM	OTHER MECHANICAL COMPLICATION OF OTHER INTERNAL ORTHOPEDIC DEVICES IMPLANTS AND GRAFTS SUBSEQUENT ENCOUNTER
Mechanical Complication	T84.117A	ICD-10 CM	BREAKDOWN (MECHANICAL) OF INTERNAL FIXATION DEVICE OF BONE OF LEFT LOWER LEG INITIAL ENCOUNTER
Mechanical Complication	T84.213D	ICD-10 CM	BREAKDOWN (MECHANICAL) OF INTERNAL FIXATION DEVICE OF BONES OF FOOT AND TOES SUBSEQUENT ENCOUNTER
Mechanical Complication	T84.318S	ICD-10 CM	BREAKDOWN (MECHANICAL) OF OTHER BONE DEVICES IMPLANTS AND GRAFTS SEQUELA
Necrosis	M87.073	ICD-10 CM	IDIOPATHIC ASEPTIC NECROSIS OF UNSPECIFIED ANKLE
Necrosis	M87.273	ICD-10 CM	OSTEONECROSIS DUE TO PREVIOUS TRAUMA UNSPECIFIED ANKLE
Necrosis	M87.171	ICD-10 CM	OSTEONECROSIS DUE TO DRUGS RIGHT ANKLE
Necrosis	M87.372	ICD-10 CM	OTHER SECONDARY OSTEONECROSIS LEFT ANKLE
Necrosis	M87.173	ICD-10 CM	OSTEONECROSIS DUE TO DRUGS UNSPECIFIED ANKLE
Necrosis	M87.872	ICD-10 CM	OTHER OSTEONECROSIS LEFT ANKLE

Necrosis	M87.871	ICD-10 CM	OTHER OSTEONECROSIS RIGHT ANKLE
Necrosis	M87.172	ICD-10 CM	OSTEONECROSIS DUE TO DRUGS LEFT ANKLE
Necrosis	M87.272	ICD-10 CM	OSTEONECROSIS DUE TO PREVIOUS TRAUMA LEFT ANKLE
Necrosis	M87.873	ICD-10 CM	OTHER OSTEONECROSIS UNSPECIFIED ANKLE
Necrosis	M87.071	ICD-10 CM	IDIOPATHIC ASEPTIC NECROSIS OF RIGHT ANKLE
Necrosis	M87.271	ICD-10 CM	OSTEONECROSIS DUE TO PREVIOUS TRAUMA RIGHT ANKLE
Necrosis	M87.072	ICD-10 CM	IDIOPATHIC ASEPTIC NECROSIS OF LEFT ANKLE
Necrosis	M87.373	ICD-10 CM	OTHER SECONDARY OSTEONECROSIS UNSPECIFIED ANKLE
Necrosis	M87.371	ICD-10 CM	OTHER SECONDARY OSTEONECROSIS RIGHT ANKLE
Nonunion	S82.302K	ICD-10 CM	UNSPECIFIED FRACTURE OF LOWER END OF LEFT TIBIA SUBSEQUENT ENCOUNTER FOR CLOSED FRACTURE WITH NONUNION
Nonunion	S82.844N	ICD-10 CM	NONDISPLACED BIMALLEOLAR FRACTURE OF RIGHT LOWER LEG SUBSEQUENT ENCOUNTER FOR OPEN FRACTURE TYPE IIIA IIIB OR IIIC WITH NONUNION
Nonunion	S82.892K	ICD-10 CM	OTHER FRACTURE OF LEFT LOWER LEG SUBSEQUENT ENCOUNTER FOR CLOSED FRACTURE WITH NONUNION
Nonunion	S82.841K	ICD-10 CM	DISPLACED BIMALLEOLAR FRACTURE OF RIGHT LOWER LEG SUBSEQUENT ENCOUNTER FOR CLOSED FRACTURE WITH NONUNION
Nonunion	S82.892N	ICD-10 CM	OTHER FRACTURE OF LEFT LOWER LEG SUBSEQUENT ENCOUNTER FOR OPEN FRACTURE TYPE IIIA IIIB OR IIIC WITH NONUNION
Nonunion	S82.61XM	ICD-10 CM	DISPLACED FRACTURE OF LATERAL MALLEOLUS OF RIGHT FIBULA SUBSEQUENT ENCOUNTER FOR OPEN FRACTURE TYPE I OR II WITH NONUNION
Nonunion	S82.61XK	ICD-10 CM	DISPLACED FRACTURE OF LATERAL MALLEOLUS OF RIGHT FIBULA SUBSEQUENT ENCOUNTER FOR CLOSED FRACTURE WITH NONUNION
Nonunion	S82.391N	ICD-10 CM	OTHER FRACTURE OF LOWER END OF RIGHT TIBIA SUBSEQUENT ENCOUNTER FOR OPEN FRACTURE TYPE IIIA IIIB OR IIIC WITH NONUNION
Nonunion	S82.309K	ICD-10 CM	UNSPECIFIED FRACTURE OF LOWER END OF UNSPECIFIED TIBIA SUBSEQUENT ENCOUNTER FOR CLOSED FRACTURE WITH NONUNION
Nonunion	S82.865M	ICD-10 CM	NONDISPLACED MAISONNEUVE'S FRACTURE OF LEFT LEG SUBSEQUENT ENCOUNTER FOR OPEN FRACTURE TYPE I OR II WITH NONUNION
Nonunion	S82.61XN	ICD-10 CM	DISPLACED FRACTURE OF LATERAL MALLEOLUS OF RIGHT FIBULA SUBSEQUENT ENCOUNTER FOR OPEN FRACTURE TYPE IIIA IIIB OR IIIC WITH NONUNION
Nonunion	S82.56XM	ICD-10 CM	NONDISPLACED FRACTURE OF MEDIAL MALLEOLUS OF UNSPECIFIED TIBIA SUBSEQUENT ENCOUNTER FOR OPEN FRACTURE TYPE I OR II WITH NONUNION
Nonunion	S82.51XM	ICD-10 CM	DISPLACED FRACTURE OF MEDIAL MALLEOLUS OF RIGHT TIBIA SUBSEQUENT ENCOUNTER FOR OPEN FRACTURE TYPE I OR II WITH NONUNION
Nonunion	S82.846K	ICD-10 CM	NONDISPLACED BIMALLEOLAR FRACTURE OF UNSPECIFIED LOWER LEG SUBSEQUENT ENCOUNTER FOR CLOSED FRACTURE WITH NONUNION
Nonunion	S82.856N	ICD-10 CM	NONDISPLACED TRIMALLEOLAR FRACTURE OF UNSPECIFIED LOWER LEG SUBSEQUENT ENCOUNTER FOR OPEN FRACTURE TYPE IIIA IIIB OR IIIC WITH NONUNION
Nonunion	S82.312K	ICD-10 CM	TORUS FRACTURE OF LOWER END OF LEFT TIBIA SUBSEQUENT ENCOUNTER FOR FRACTURE WITH NONUNION
Nonunion	S82.301M	ICD-10 CM	UNSPECIFIED FRACTURE OF LOWER END OF RIGHT TIBIA SUBSEQUENT ENCOUNTER FOR OPEN FRACTURE TYPE I OR II WITH NONUNION
Nonunion	S82.64XN	ICD-10 CM	NONDISPLACED FRACTURE OF LATERAL MALLEOLUS OF RIGHT FIBULA SUBSEQUENT ENCOUNTER FOR OPEN FRACTURE TYPE IIIA IIIB OR IIIC WITH NONUNION
Nonunion	S82.843N	ICD-10 CM	DISPLACED BIMALLEOLAR FRACTURE OF UNSPECIFIED LOWER LEG SUBSEQUENT ENCOUNTER FOR OPEN FRACTURE TYPE IIIA IIIB OR IIIC WITH NONUNION
Nonunion	S82.391M	ICD-10 CM	OTHER FRACTURE OF LOWER END OF RIGHT TIBIA SUBSEQUENT ENCOUNTER FOR OPEN FRACTURE TYPE I OR II WITH NONUNION
Nonunion	S82.311K	ICD-10 CM	TORUS FRACTURE OF LOWER END OF RIGHT TIBIA SUBSEQUENT ENCOUNTER FOR FRACTURE WITH NONUNION
Nonunion	S82.876K	ICD-10 CM	NONDISPLACED PILON FRACTURE OF UNSPECIFIED TIBIA SUBSEQUENT ENCOUNTER FOR CLOSED FRACTURE WITH NONUNION
Nonunion	S82.841N	ICD-10 CM	DISPLACED BIMALLEOLAR FRACTURE OF RIGHT LOWER LEG SUBSEQUENT ENCOUNTER FOR OPEN FRACTURE TYPE IIIA IIIB OR IIIC WITH NONUNION
Nonunion	S82.874K	ICD-10 CM	NONDISPLACED PILON FRACTURE OF RIGHT TIBIA SUBSEQUENT ENCOUNTER FOR CLOSED FRACTURE WITH NONUNION
Nonunion	S82.862M	ICD-10 CM	DISPLACED MAISONNEUVE'S FRACTURE OF LEFT LEG SUBSEQUENT ENCOUNTER FOR OPEN FRACTURE TYPE I OR II WITH NONUNION
Nonunion	S82.876N	ICD-10 CM	NONDISPLACED PILON FRACTURE OF UNSPECIFIED TIBIA SUBSEQUENT ENCOUNTER FOR OPEN FRACTURE TYPE IIIA IIIB OR IIIC WITH NONUNION
Nonunion	S82.51XK	ICD-10 CM	DISPLACED FRACTURE OF MEDIAL MALLEOLUS OF RIGHT TIBIA SUBSEQUENT ENCOUNTER FOR CLOSED FRACTURE WITH NONUNION
Nonunion	S82.845M	ICD-10 CM	NONDISPLACED BIMALLEOLAR FRACTURE OF LEFT LOWER LEG SUBSEQUENT ENCOUNTER FOR OPEN FRACTURE TYPE I OR II WITH NONUNION
Nonunion	S82.62XN	ICD-10 CM	DISPLACED FRACTURE OF LATERAL MALLEOLUS OF LEFT FIBULA SUBSEQUENT ENCOUNTER FOR OPEN FRACTURE TYPE IIIA IIIB OR IIIC WITH NONUNION

Nonunion	S82.302M	ICD-10 CM	UNSPECIFIED FRACTURE OF LOWER END OF LEFT TIBIA SUBSEQUENT ENCOUNTER FOR OPEN FRACTURE TYPE I OR II WITH NONUNION
Nonunion	S82.62XM	ICD-10 CM	DISPLACED FRACTURE OF LATERAL MALLEOLUS OF LEFT FIBULA SUBSEQUENT ENCOUNTER FOR OPEN FRACTURE TYPE I OR II WITH NONUNION
Nonunion	S82.865K	ICD-10 CM	NONDISPLACED MAISONNEUVE'S FRACTURE OF LEFT LEG SUBSEQUENT ENCOUNTER FOR CLOSED FRACTURE WITH NONUNION
Nonunion	S82.842N	ICD-10 CM	DISPLACED BIMALLEOLAR FRACTURE OF LEFT LOWER LEG SUBSEQUENT ENCOUNTER FOR OPEN FRACTURE TYPE IIIA IIIB OR IIIC WITH NONUNION
Nonunion	S82.66XK	ICD-10 CM	NONDISPLACED FRACTURE OF LATERAL MALLEOLUS OF UNSPECIFIED FIBULA SUBSEQUENT ENCOUNTER FOR CLOSED FRACTURE WITH NONUNION
Nonunion	S82.871N	ICD-10 CM	DISPLACED PILON FRACTURE OF RIGHT TIBIA SUBSEQUENT ENCOUNTER FOR OPEN FRACTURE TYPE IIIA IIIB OR IIIC WITH NONUNION
Nonunion	S82.301N	ICD-10 CM	UNSPECIFIED FRACTURE OF LOWER END OF RIGHT TIBIA SUBSEQUENT ENCOUNTER FOR OPEN FRACTURE TYPE IIIA IIIB OR IIIC WITH NONUNION
Nonunion	S82.845N	ICD-10 CM	NONDISPLACED BIMALLEOLAR FRACTURE OF LEFT LOWER LEG SUBSEQUENT ENCOUNTER FOR OPEN FRACTURE TYPE IIIA IIIB OR IIIC WITH NONUNION
Nonunion	S82.856M	ICD-10 CM	NONDISPLACED TRIMALLEOLAR FRACTURE OF UNSPECIFIED LOWER LEG SUBSEQUENT ENCOUNTER FOR OPEN FRACTURE TYPE I OR II WITH NONUNION
Nonunion	S82.873M	ICD-10 CM	DISPLACED PILON FRACTURE OF UNSPECIFIED TIBIA SUBSEQUENT ENCOUNTER FOR OPEN FRACTURE TYPE I OR II WITH NONUNION
Nonunion	S82.53XK	ICD-10 CM	DISPLACED FRACTURE OF MEDIAL MALLEOLUS OF UNSPECIFIED TIBIA SUBSEQUENT ENCOUNTER FOR CLOSED FRACTURE WITH NONUNION
Nonunion	S82.899M	ICD-10 CM	OTHER FRACTURE OF UNSPECIFIED LOWER LEG SUBSEQUENT ENCOUNTER FOR OPEN FRACTURE TYPE I OR II WITH NONUNION
Nonunion	S82.846M	ICD-10 CM	NONDISPLACED BIMALLEOLAR FRACTURE OF UNSPECIFIED LOWER LEG SUBSEQUENT ENCOUNTER FOR OPEN FRACTURE TYPE I OR II WITH NONUNION
Nonunion	S82.855M	ICD-10 CM	NONDISPLACED TRIMALLEOLAR FRACTURE OF LEFT LOWER LEG SUBSEQUENT ENCOUNTER FOR OPEN FRACTURE TYPE I OR II WITH NONUNION
Nonunion	S82.302N	ICD-10 CM	UNSPECIFIED FRACTURE OF LOWER END OF LEFT TIBIA SUBSEQUENT ENCOUNTER FOR OPEN FRACTURE TYPE IIIA IIIB OR IIIC WITH NONUNION
Nonunion	S82.872K	ICD-10 CM	DISPLACED PILON FRACTURE OF LEFT TIBIA SUBSEQUENT ENCOUNTER FOR CLOSED FRACTURE WITH NONUNION
Nonunion	S82.54XM	ICD-10 CM	NONDISPLACED FRACTURE OF MEDIAL MALLEOLUS OF RIGHT TIBIA SUBSEQUENT ENCOUNTER FOR OPEN FRACTURE TYPE I OR II WITH NONUNION
Nonunion	S82.54XN	ICD-10 CM	NONDISPLACED FRACTURE OF MEDIAL MALLEOLUS OF RIGHT TIBIA SUBSEQUENT ENCOUNTER FOR OPEN FRACTURE TYPE IIIA IIIB OR IIIC WITH NONUNION
Nonunion	S82.899K	ICD-10 CM	OTHER FRACTURE OF UNSPECIFIED LOWER LEG SUBSEQUENT ENCOUNTER FOR CLOSED FRACTURE WITH NONUNION
Nonunion	S82.875N	ICD-10 CM	NONDISPLACED PILON FRACTURE OF LEFT TIBIA SUBSEQUENT ENCOUNTER FOR OPEN FRACTURE TYPE IIIA IIIB OR IIIC WITH NONUNION
Nonunion	S82.844M	ICD-10 CM	NONDISPLACED BIMALLEOLAR FRACTURE OF RIGHT LOWER LEG SUBSEQUENT ENCOUNTER FOR OPEN FRACTURE TYPE I OR II WITH NONUNION
Nonunion	S82.876M	ICD-10 CM	NONDISPLACED PILON FRACTURE OF UNSPECIFIED TIBIA SUBSEQUENT ENCOUNTER FOR OPEN FRACTURE TYPE I OR II WITH NONUNION
Nonunion	S82.52XK	ICD-10 CM	DISPLACED FRACTURE OF MEDIAL MALLEOLUS OF LEFT TIBIA SUBSEQUENT ENCOUNTER FOR CLOSED FRACTURE WITH NONUNION
Nonunion	S82.845K	ICD-10 CM	NONDISPLACED BIMALLEOLAR FRACTURE OF LEFT LOWER LEG SUBSEQUENT ENCOUNTER FOR CLOSED FRACTURE WITH NONUNION
Nonunion	S82.855N	ICD-10 CM	NONDISPLACED TRIMALLEOLAR FRACTURE OF LEFT LOWER LEG SUBSEQUENT ENCOUNTER FOR OPEN FRACTURE TYPE IIIA IIIB OR IIIC WITH NONUNION
Nonunion	S82.853N	ICD-10 CM	DISPLACED TRIMALLEOLAR FRACTURE OF UNSPECIFIED LOWER LEG SUBSEQUENT ENCOUNTER FOR OPEN FRACTURE TYPE IIIA IIIB OR IIIC WITH NONUNION
Nonunion	S82.53XM	ICD-10 CM	DISPLACED FRACTURE OF MEDIAL MALLEOLUS OF UNSPECIFIED TIBIA SUBSEQUENT ENCOUNTER FOR OPEN FRACTURE TYPE I OR II WITH NONUNION
Nonunion	S82.891M	ICD-10 CM	OTHER FRACTURE OF RIGHT LOWER LEG SUBSEQUENT ENCOUNTER FOR OPEN FRACTURE TYPE I OR II WITH NONUNION
Nonunion	S82.844K	ICD-10 CM	NONDISPLACED BIMALLEOLAR FRACTURE OF RIGHT LOWER LEG SUBSEQUENT ENCOUNTER FOR CLOSED FRACTURE WITH NONUNION
Nonunion	S82.66XM	ICD-10 CM	NONDISPLACED FRACTURE OF LATERAL MALLEOLUS OF UNSPECIFIED FIBULA SUBSEQUENT ENCOUNTER FOR OPEN FRACTURE TYPE I OR II WITH NONUNION
Nonunion	S82.392M	ICD-10 CM	OTHER FRACTURE OF LOWER END OF LEFT TIBIA SUBSEQUENT ENCOUNTER FOR OPEN FRACTURE TYPE I OR II WITH NONUNION
Nonunion	S82.851M	ICD-10 CM	DISPLACED TRIMALLEOLAR FRACTURE OF RIGHT LOWER LEG SUBSEQUENT ENCOUNTER FOR OPEN FRACTURE TYPE I OR II WITH NONUNION
Nonunion	S82.52XN	ICD-10 CM	DISPLACED FRACTURE OF MEDIAL MALLEOLUS OF LEFT TIBIA SUBSEQUENT ENCOUNTER FOR OPEN FRACTURE TYPE IIIA IIIB OR IIIC WITH NONUNION
Nonunion	S82.843K	ICD-10 CM	DISPLACED BIMALLEOLAR FRACTURE OF UNSPECIFIED LOWER LEG SUBSEQUENT ENCOUNTER FOR CLOSED FRACTURE WITH NONUNION

Nonunion	S82.53XN	ICD-10 CM	DISPLACED FRACTURE OF MEDIAL MALLEOLUS OF UNSPECIFIED TIBIA SUBSEQUENT ENCOUNTER FOR OPEN FRACTURE TYPE IIIA IIIB OR IIIC WITH NONUNION
Nonunion	S82.874N	ICD-10 CM	NONDISPLACED PILON FRACTURE OF RIGHT TIBIA SUBSEQUENT ENCOUNTER FOR OPEN FRACTURE TYPE IIIA IIIB OR IIIC WITH NONUNION
Nonunion	S82.55XN	ICD-10 CM	NONDISPLACED FRACTURE OF MEDIAL MALLEOLUS OF LEFT TIBIA SUBSEQUENT ENCOUNTER FOR OPEN FRACTURE TYPE IIIA IIIB OR IIIC WITH NONUNION
Nonunion	S82.873N	ICD-10 CM	DISPLACED PILON FRACTURE OF UNSPECIFIED TIBIA SUBSEQUENT ENCOUNTER FOR OPEN FRACTURE TYPE IIIA IIIB OR IIIC WITH NONUNION
Nonunion	S82.853M	ICD-10 CM	DISPLACED TRIMALLEOLAR FRACTURE OF UNSPECIFIED LOWER LEG SUBSEQUENT ENCOUNTER FOR OPEN FRACTURE TYPE I OR II WITH NONUNION
Nonunion	S82.65XN	ICD-10 CM	NONDISPLACED FRACTURE OF LATERAL MALLEOLUS OF LEFT FIBULA SUBSEQUENT ENCOUNTER FOR OPEN FRACTURE TYPE IIIA IIIB OR IIIC WITH NONUNION
Nonunion	S82.864N	ICD-10 CM	NONDISPLACED MAISONNEUVE'S FRACTURE OF RIGHT LEG SUBSEQUENT ENCOUNTER FOR OPEN FRACTURE TYPE IIIA IIIB OR IIIC WITH NONUNION
Nonunion	S82.872N	ICD-10 CM	DISPLACED PILON FRACTURE OF LEFT TIBIA SUBSEQUENT ENCOUNTER FOR OPEN FRACTURE TYPE IIIA IIIB OR IIIC WITH NONUNION
Nonunion	S82.65XK	ICD-10 CM	NONDISPLACED FRACTURE OF LATERAL MALLEOLUS OF LEFT FIBULA SUBSEQUENT ENCOUNTER FOR CLOSED FRACTURE WITH NONUNION
Nonunion	S82.863K	ICD-10 CM	DISPLACED MAISONNEUVE'S FRACTURE OF UNSPECIFIED LEG SUBSEQUENT ENCOUNTER FOR CLOSED FRACTURE WITH NONUNION
Nonunion	S82.55XK	ICD-10 CM	NONDISPLACED FRACTURE OF MEDIAL MALLEOLUS OF LEFT TIBIA SUBSEQUENT ENCOUNTER FOR CLOSED FRACTURE WITH NONUNION
Nonunion	S82.856K	ICD-10 CM	NONDISPLACED TRIMALLEOLAR FRACTURE OF UNSPECIFIED LOWER LEG SUBSEQUENT ENCOUNTER FOR CLOSED FRACTURE WITH NONUNION
Nonunion	S82.64XK	ICD-10 CM	NONDISPLACED FRACTURE OF LATERAL MALLEOLUS OF RIGHT FIBULA SUBSEQUENT ENCOUNTER FOR CLOSED FRACTURE WITH NONUNION
Nonunion	S82.66XN	ICD-10 CM	NONDISPLACED FRACTURE OF LATERAL MALLEOLUS OF UNSPECIFIED FIBULA SUBSEQUENT ENCOUNTER FOR OPEN FRACTURE TYPE IIIA IIIB OR IIIC WITH NONUNION
Nonunion	S82.842M	ICD-10 CM	DISPLACED BIMALLEOLAR FRACTURE OF LEFT LOWER LEG SUBSEQUENT ENCOUNTER FOR OPEN FRACTURE TYPE I OR II WITH NONUNION
Nonunion	S82.854K	ICD-10 CM	NONDISPLACED TRIMALLEOLAR FRACTURE OF RIGHT LOWER LEG SUBSEQUENT ENCOUNTER FOR CLOSED FRACTURE WITH NONUNION
Nonunion	S82.875K	ICD-10 CM	NONDISPLACED PILON FRACTURE OF LEFT TIBIA SUBSEQUENT ENCOUNTER FOR CLOSED FRACTURE WITH NONUNION
Nonunion	S82.866K	ICD-10 CM	NONDISPLACED MAISONNEUVE'S FRACTURE OF UNSPECIFIED LEG SUBSEQUENT ENCOUNTER FOR CLOSED FRACTURE WITH NONUNION
Nonunion	S82.864M	ICD-10 CM	NONDISPLACED MAISONNEUVE'S FRACTURE OF RIGHT LEG SUBSEQUENT ENCOUNTER FOR OPEN FRACTURE TYPE I OR II WITH NONUNION
Nonunion	S82.851N	ICD-10 CM	DISPLACED TRIMALLEOLAR FRACTURE OF RIGHT LOWER LEG SUBSEQUENT ENCOUNTER FOR OPEN FRACTURE TYPE IIIA IIIB OR IIIC WITH NONUNION
Nonunion	S82.54XK	ICD-10 CM	NONDISPLACED FRACTURE OF MEDIAL MALLEOLUS OF RIGHT TIBIA SUBSEQUENT ENCOUNTER FOR CLOSED FRACTURE WITH NONUNION
Nonunion	S82.852N	ICD-10 CM	DISPLACED TRIMALLEOLAR FRACTURE OF LEFT LOWER LEG SUBSEQUENT ENCOUNTER FOR OPEN FRACTURE TYPE IIIA IIIB OR IIIC WITH NONUNION
Nonunion	S82.399N	ICD-10 CM	OTHER FRACTURE OF LOWER END OF UNSPECIFIED TIBIA SUBSEQUENT ENCOUNTER FOR OPEN FRACTURE TYPE IIIA IIIB OR IIIC WITH NONUNION
Nonunion	S82.65XM	ICD-10 CM	NONDISPLACED FRACTURE OF LATERAL MALLEOLUS OF LEFT FIBULA SUBSEQUENT ENCOUNTER FOR OPEN FRACTURE TYPE I OR II WITH NONUNION
Nonunion	S82.392N	ICD-10 CM	OTHER FRACTURE OF LOWER END OF LEFT TIBIA SUBSEQUENT ENCOUNTER FOR OPEN FRACTURE TYPE IIIA IIIB OR IIIC WITH NONUNION
Nonunion	S82.861M	ICD-10 CM	DISPLACED MAISONNEUVE'S FRACTURE OF RIGHT LEG SUBSEQUENT ENCOUNTER FOR OPEN FRACTURE TYPE I OR II WITH NONUNION
Nonunion	S82.899N	ICD-10 CM	OTHER FRACTURE OF UNSPECIFIED LOWER LEG SUBSEQUENT ENCOUNTER FOR OPEN FRACTURE TYPE IIIA IIIB OR IIIC WITH NONUNION
Nonunion	S82.391K	ICD-10 CM	OTHER FRACTURE OF LOWER END OF RIGHT TIBIA SUBSEQUENT ENCOUNTER FOR CLOSED FRACTURE WITH NONUNION
Nonunion	S82.874M	ICD-10 CM	NONDISPLACED PILON FRACTURE OF RIGHT TIBIA SUBSEQUENT ENCOUNTER FOR OPEN FRACTURE TYPE I OR II WITH NONUNION
Nonunion	S82.863M	ICD-10 CM	DISPLACED MAISONNEUVE'S FRACTURE OF UNSPECIFIED LEG SUBSEQUENT ENCOUNTER FOR OPEN FRACTURE TYPE I OR II WITH NONUNION
Nonunion	S82.855K	ICD-10 CM	NONDISPLACED TRIMALLEOLAR FRACTURE OF LEFT LOWER LEG SUBSEQUENT ENCOUNTER FOR CLOSED FRACTURE WITH NONUNION
Nonunion	S82.854M	ICD-10 CM	NONDISPLACED TRIMALLEOLAR FRACTURE OF RIGHT LOWER LEG SUBSEQUENT ENCOUNTER FOR OPEN FRACTURE TYPE I OR II WITH NONUNION
Nonunion	S82.891N	ICD-10 CM	OTHER FRACTURE OF RIGHT LOWER LEG SUBSEQUENT ENCOUNTER FOR OPEN FRACTURE TYPE IIIA IIIB OR IIIC WITH NONUNION
Nonunion	S82.399M	ICD-10 CM	OTHER FRACTURE OF LOWER END OF UNSPECIFIED TIBIA SUBSEQUENT ENCOUNTER FOR OPEN FRACTURE TYPE I OR II WITH NONUNION

Nonunion	S82.875M	ICD-10 CM	NONDISPLACED PILON FRACTURE OF LEFT TIBIA SUBSEQUENT ENCOUNTER FOR OPEN FRACTURE TYPE I OR II WITH NONUNION
Nonunion	S82.854N	ICD-10 CM	NONDISPLACED TRIMALLEOLAR FRACTURE OF RIGHT LOWER LEG SUBSEQUENT ENCOUNTER FOR OPEN FRACTURE TYPE IIIA IIIB OR IIIC WITH NONUNION
Nonunion	S82.865N	ICD-10 CM	NONDISPLACED MAISONNEUVE'S FRACTURE OF LEFT LEG SUBSEQUENT ENCOUNTER FOR OPEN FRACTURE TYPE IIIA IIIB OR IIIC WITH NONUNION
Nonunion	S82.853K	ICD-10 CM	DISPLACED TRIMALLEOLAR FRACTURE OF UNSPECIFIED LOWER LEG SUBSEQUENT ENCOUNTER FOR CLOSED FRACTURE WITH NONUNION
Nonunion	S82.863N	ICD-10 CM	DISPLACED MAISONNEUVE'S FRACTURE OF UNSPECIFIED LEG SUBSEQUENT ENCOUNTER FOR OPEN FRACTURE TYPE IIIA IIIB OR IIIC WITH NONUNION
Nonunion	S82.862N	ICD-10 CM	DISPLACED MAISONNEUVE'S FRACTURE OF LEFT LEG SUBSEQUENT ENCOUNTER FOR OPEN FRACTURE TYPE IIIA IIIB OR IIIC WITH NONUNION
Nonunion	S82.851K	ICD-10 CM	DISPLACED TRIMALLEOLAR FRACTURE OF RIGHT LOWER LEG SUBSEQUENT ENCOUNTER FOR CLOSED FRACTURE WITH NONUNION
Nonunion	S82.872M	ICD-10 CM	DISPLACED PILON FRACTURE OF LEFT TIBIA SUBSEQUENT ENCOUNTER FOR OPEN FRACTURE TYPE I OR II WITH NONUNION
Nonunion	S82.842K	ICD-10 CM	DISPLACED BIMALLEOLAR FRACTURE OF LEFT LOWER LEG SUBSEQUENT ENCOUNTER FOR CLOSED FRACTURE WITH NONUNION
Nonunion	S82.309N	ICD-10 CM	UNSPECIFIED FRACTURE OF LOWER END OF UNSPECIFIED TIBIA SUBSEQUENT ENCOUNTER FOR OPEN FRACTURE TYPE IIIA IIIB OR IIIC WITH NONUNION
Nonunion	S82.841M	ICD-10 CM	DISPLACED BIMALLEOLAR FRACTURE OF RIGHT LOWER LEG SUBSEQUENT ENCOUNTER FOR OPEN FRACTURE TYPE I OR II WITH NONUNION
Nonunion	S82.63XM	ICD-10 CM	DISPLACED FRACTURE OF LATERAL MALLEOLUS OF UNSPECIFIED FIBULA SUBSEQUENT ENCOUNTER FOR OPEN FRACTURE TYPE I OR II WITH NONUNION
Nonunion	S82.871M	ICD-10 CM	DISPLACED PILON FRACTURE OF RIGHT TIBIA SUBSEQUENT ENCOUNTER FOR OPEN FRACTURE TYPE I OR II WITH NONUNION
Nonunion	S82.63XN	ICD-10 CM	DISPLACED FRACTURE OF LATERAL MALLEOLUS OF UNSPECIFIED FIBULA SUBSEQUENT ENCOUNTER FOR OPEN FRACTURE TYPE IIIA IIIB OR IIIC WITH NONUNION
Nonunion	S82.56XN	ICD-10 CM	NONDISPLACED FRACTURE OF MEDIAL MALLEOLUS OF UNSPECIFIED TIBIA SUBSEQUENT ENCOUNTER FOR OPEN FRACTURE TYPE IIIA IIIB OR IIIC WITH NONUNION
Nonunion	S82.64XM	ICD-10 CM	NONDISPLACED FRACTURE OF LATERAL MALLEOLUS OF RIGHT FIBULA SUBSEQUENT ENCOUNTER FOR OPEN FRACTURE TYPE I OR II WITH NONUNION
Nonunion	S82.892M	ICD-10 CM	OTHER FRACTURE OF LEFT LOWER LEG SUBSEQUENT ENCOUNTER FOR OPEN FRACTURE TYPE I OR II WITH NONUNION
Nonunion	S82.309M	ICD-10 CM	UNSPECIFIED FRACTURE OF LOWER END OF UNSPECIFIED TIBIA SUBSEQUENT ENCOUNTER FOR OPEN FRACTURE TYPE I OR II WITH NONUNION
Nonunion	S82.301K	ICD-10 CM	UNSPECIFIED FRACTURE OF LOWER END OF RIGHT TIBIA SUBSEQUENT ENCOUNTER FOR CLOSED FRACTURE WITH NONUNION
Nonunion	S82.55XM	ICD-10 CM	NONDISPLACED FRACTURE OF MEDIAL MALLEOLUS OF LEFT TIBIA SUBSEQUENT ENCOUNTER FOR OPEN FRACTURE TYPE I OR II WITH NONUNION
Nonunion	S82.51XN	ICD-10 CM	DISPLACED FRACTURE OF MEDIAL MALLEOLUS OF RIGHT TIBIA SUBSEQUENT ENCOUNTER FOR OPEN FRACTURE TYPE IIIA IIIB OR IIIC WITH NONUNION
Nonunion	S82.861N	ICD-10 CM	DISPLACED MAISONNEUVE'S FRACTURE OF RIGHT LEG SUBSEQUENT ENCOUNTER FOR OPEN FRACTURE TYPE IIIA IIIB OR IIIC WITH NONUNION
Nonunion	S82.52XM	ICD-10 CM	DISPLACED FRACTURE OF MEDIAL MALLEOLUS OF LEFT TIBIA SUBSEQUENT ENCOUNTER FOR OPEN FRACTURE TYPE I OR II WITH NONUNION
Nonunion	S82.319K	ICD-10 CM	TORUS FRACTURE OF LOWER END OF UNSPECIFIED TIBIA SUBSEQUENT ENCOUNTER FOR FRACTURE WITH NONUNION
Nonunion	S82.866N	ICD-10 CM	NONDISPLACED MAISONNEUVE'S FRACTURE OF UNSPECIFIED LEG SUBSEQUENT ENCOUNTER FOR OPEN FRACTURE TYPE IIIA IIIB OR IIIC WITH NONUNION
Nonunion	S82.846N	ICD-10 CM	NONDISPLACED BIMALLEOLAR FRACTURE OF UNSPECIFIED LOWER LEG SUBSEQUENT ENCOUNTER FOR OPEN FRACTURE TYPE IIIA IIIB OR IIIC WITH NONUNION
Nonunion	S82.852K	ICD-10 CM	DISPLACED TRIMALLEOLAR FRACTURE OF LEFT LOWER LEG SUBSEQUENT ENCOUNTER FOR CLOSED FRACTURE WITH NONUNION
Nonunion	S82.62XK	ICD-10 CM	DISPLACED FRACTURE OF LATERAL MALLEOLUS OF LEFT FIBULA SUBSEQUENT ENCOUNTER FOR CLOSED FRACTURE WITH NONUNION
Nonunion	S82.861K	ICD-10 CM	DISPLACED MAISONNEUVE'S FRACTURE OF RIGHT LEG SUBSEQUENT ENCOUNTER FOR CLOSED FRACTURE WITH NONUNION
Nonunion	S82.843M	ICD-10 CM	DISPLACED BIMALLEOLAR FRACTURE OF UNSPECIFIED LOWER LEG SUBSEQUENT ENCOUNTER FOR OPEN FRACTURE TYPE I OR II WITH NONUNION
Nonunion	S82.866M	ICD-10 CM	NONDISPLACED MAISONNEUVE'S FRACTURE OF UNSPECIFIED LEG SUBSEQUENT ENCOUNTER FOR OPEN FRACTURE TYPE I OR II WITH NONUNION
Nonunion	S82.852M	ICD-10 CM	DISPLACED TRIMALLEOLAR FRACTURE OF LEFT LOWER LEG SUBSEQUENT ENCOUNTER FOR OPEN FRACTURE TYPE I OR II WITH NONUNION
Nonunion	S82.392K	ICD-10 CM	OTHER FRACTURE OF LOWER END OF LEFT TIBIA SUBSEQUENT ENCOUNTER FOR CLOSED FRACTURE WITH NONUNION
Nonunion	S82.862K	ICD-10 CM	DISPLACED MAISONNEUVE'S FRACTURE OF LEFT LEG SUBSEQUENT ENCOUNTER FOR CLOSED FRACTURE WITH NONUNION

Nonunion	S82.873K	ICD-10 CM	DISPLACED PILON FRACTURE OF UNSPECIFIED TIBIA SUBSEQUENT ENCOUNTER FOR CLOSED FRACTURE WITH NONUNION
Nonunion	S82.56XK	ICD-10 CM	NONDISPLACED FRACTURE OF MEDIAL MALLEOLUS OF UNSPECIFIED TIBIA SUBSEQUENT ENCOUNTER FOR CLOSED FRACTURE WITH NONUNION
Nonunion	S82.864K	ICD-10 CM	NONDISPLACED MAISONNEUVE'S FRACTURE OF RIGHT LEG SUBSEQUENT ENCOUNTER FOR CLOSED FRACTURE WITH NONUNION
Nonunion	S82.871K	ICD-10 CM	DISPLACED PILON FRACTURE OF RIGHT TIBIA SUBSEQUENT ENCOUNTER FOR CLOSED FRACTURE WITH NONUNION
Nonunion	S82.891K	ICD-10 CM	OTHER FRACTURE OF RIGHT LOWER LEG SUBSEQUENT ENCOUNTER FOR CLOSED FRACTURE WITH NONUNION
Nonunion	S82.399K	ICD-10 CM	OTHER FRACTURE OF LOWER END OF UNSPECIFIED TIBIA SUBSEQUENT ENCOUNTER FOR CLOSED FRACTURE WITH NONUNION
Nonunion	S82.63XK	ICD-10 CM	DISPLACED FRACTURE OF LATERAL MALLEOLUS OF UNSPECIFIED FIBULA SUBSEQUENT ENCOUNTER FOR CLOSED FRACTURE WITH NONUNION
Other Complication	T84.89XA	ICD-10 CM	OTHER SPECIFIED COMPLICATION OF INTERNAL ORTHOPEDIC PROSTHETIC DEVICES IMPLANTS AND GRAFTS INITIAL ENCOUNTER
Other Complication	M67.00	ICD-10 CM	SHORT ACHILLES TENDON (ACQUIRED) UNSPECIFIED ANKLE
Other Complication	T79.A29D	ICD-10 CM	TRAUMATIC COMPARTMENT SYNDROME OF UNSPECIFIED LOWER EXTREMITY SUBSEQUENT ENCOUNTER
Other Complication	T84.83XD	ICD-10 CM	HEMORRHAGE DUE TO INTERNAL ORTHOPEDIC PROSTHETIC DEVICES IMPLANTS AND GRAFTS SUBSEQUENT ENCOUNTER
Other Complication	M71.572	ICD-10 CM	OTHER BURSITIS NOT ELSEWHERE CLASSIFIED LEFT ANKLE AND FOOT
Other Complication	M85.179	ICD-10 CM	SKELETAL FLUOROSIS UNSPECIFIED ANKLE AND FOOT
Other Complication	M62.472	ICD-10 CM	CONTRACTURE OF MUSCLE LEFT ANKLE AND FOOT
Other Complication	M61.271	ICD-10 CM	PARALYTIC CALCIFICATION AND OSSIFICATION OF MUSCLE RIGHT ANKLE AND FOOT
Other Complication	M67.272	ICD-10 CM	SYNOVIAL HYPERTROPHY NOT ELSEWHERE CLASSIFIED LEFT ANKLE AND FOOT
Other Complication	T79.A29A	ICD-10 CM	TRAUMATIC COMPARTMENT SYNDROME OF UNSPECIFIED LOWER EXTREMITY INITIAL ENCOUNTER
Other Complication	M71.472	ICD-10 CM	CALCIUM DEPOSIT IN BURSA LEFT ANKLE AND FOOT
Other Complication	M61.071	ICD-10 CM	MYOSITIS OSSIFICANS TRAUMATICA RIGHT ANKLE AND FOOT
Other Complication	T84.86XD	ICD-10 CM	THROMBOSIS DUE TO INTERNAL ORTHOPEDIC PROSTHETIC DEVICES IMPLANTS AND GRAFTS SUBSEQUENT ENCOUNTER
Other Complication	M93.279	ICD-10 CM	OSTEOCHONDRITIS DISSECANS UNSPECIFIED ANKLE AND JOINTS OF FOOT
Other Complication	M60.279	ICD-10 CM	FOREIGN BODY GRANULOMA OF SOFT TISSUE NOT ELSEWHERE CLASSIFIED UNSPECIFIED ANKLE AND FOOT
Other Complication	M93.979	ICD-10 CM	OSTEOCHONDROPATHY UNSPECIFIED UNSPECIFIED ANKLE AND FOOT
Other Complication	M67.872	ICD-10 CM	OTHER SPECIFIED DISORDERS OF SYNOVIUM LEFT ANKLE AND FOOT
Other Complication	M65.279	ICD-10 CM	CALCIFIC TENDINITIS UNSPECIFIED ANKLE AND FOOT
Other Complication	T84.81XS	ICD-10 CM	EMBOLISM DUE TO INTERNAL ORTHOPEDIC PROSTHETIC DEVICES IMPLANTS AND GRAFTS SEQUELA
Other Complication	T84.83XA	ICD-10 CM	HEMORRHAGE DUE TO INTERNAL ORTHOPEDIC PROSTHETIC DEVICES IMPLANTS AND GRAFTS INITIAL ENCOUNTER
Other Complication	M93.972	ICD-10 CM	OSTEOCHONDROPATHY UNSPECIFIED LEFT ANKLE AND FOOT
Other Complication	M90.572	ICD-10 CM	OSTEONECROSIS IN DISEASES CLASSIFIED ELSEWHERE LEFT ANKLE AND FOOT
Other Complication	M89.079	ICD-10 CM	ALGONEURODYSTROPHY UNSPECIFIED ANKLE AND FOOT
Other Complication	M71.871	ICD-10 CM	OTHER SPECIFIED BURSOPATHIES RIGHT ANKLE AND FOOT
Other Complication	M60.871	ICD-10 CM	OTHER MYOSITIS RIGHT ANKLE AND FOOT
Other Complication	M67.871	ICD-10 CM	OTHER SPECIFIED DISORDERS OF SYNOVIUM RIGHT ANKLE AND FOOT
Other Complication	M61.079	ICD-10 CM	MYOSITIS OSSIFICANS TRAUMATICA UNSPECIFIED ANKLE AND FOOT
Other Complication	M94.271	ICD-10 CM	CHONDROMALACIA RIGHT ANKLE AND JOINTS OF RIGHT FOOT
Other Complication	M61.479	ICD-10 CM	OTHER CALCIFICATION OF MUSCLE UNSPECIFIED ANKLE AND FOOT
Other Complication	M93.872	ICD-10 CM	OTHER SPECIFIED OSTEOCHONDROPATHIES LEFT ANKLE AND FOOT
Other Complication	M62.172	ICD-10 CM	OTHER RUPTURE OF MUSCLE (NONTRAUMATIC) LEFT ANKLE AND FOOT
Other Complication	M71.372	ICD-10 CM	OTHER BURSAL CYST LEFT ANKLE AND FOOT
Other Complication	T84.82XS	ICD-10 CM	FIBROSIS DUE TO INTERNAL ORTHOPEDIC PROSTHETIC DEVICES IMPLANTS AND GRAFTS SEQUELA
Other Complication	T84.86XS	ICD-10 CM	THROMBOSIS DUE TO INTERNAL ORTHOPEDIC PROSTHETIC DEVICES IMPLANTS AND GRAFTS SEQUELA
Other Complication	M61.272	ICD-10 CM	PARALYTIC CALCIFICATION AND OSSIFICATION OF MUSCLE LEFT ANKLE AND FOOT
Other Complication	M61.472	ICD-10 CM	OTHER CALCIFICATION OF MUSCLE LEFT ANKLE AND FOOT
Other Complication	M71.379	ICD-10 CM	OTHER BURSAL CYST UNSPECIFIED ANKLE AND FOOT
Other Complication	M89.579	ICD-10 CM	OSTEOLYSIS UNSPECIFIED ANKLE AND FOOT
Other Complication	M61.572	ICD-10 CM	OTHER OSSIFICATION OF MUSCLE LEFT ANKLE AND FOOT
Other Complication	M94.8X7	ICD-10 CM	OTHER SPECIFIED DISORDERS OF CARTILAGE ANKLE AND FOOT
Other Complication	M85.679	ICD-10 CM	OTHER CYST OF BONE UNSPECIFIED ANKLE AND FOOT
Other Complication	M61.571	ICD-10 CM	OTHER OSSIFICATION OF MUSCLE RIGHT ANKLE AND FOOT
Other Complication	M85.079	ICD-10 CM	FIBROUS DYSPLASIA (MONOSTOTIC) UNSPECIFIED ANKLE AND FOOT

Other Complication	T84.82XA	ICD-10 CM	FIBROSIS DUE TO INTERNAL ORTHOPEDIC PROSTHETIC DEVICES IMPLANTS AND GRAFTS INITIAL ENCOUNTER
Other Complication	M90.879	ICD-10 CM	OSTEOPATHY IN DISEASES CLASSIFIED ELSEWHERE UNSPECIFIED ANKLE AND FOOT
Other Complication	M93.272	ICD-10 CM	OSTEOCHONDRITIS DISSECANS LEFT ANKLE AND JOINTS OF LEFT FOOT
Other Complication	M85.871	ICD-10 CM	OTHER SPECIFIED DISORDERS OF BONE DENSITY AND STRUCTURE RIGHT ANKLE AND FOOT
Other Complication	M67.371	ICD-10 CM	TRANSIENT SYNOVITIS RIGHT ANKLE AND FOOT
Other Complication	T84.84XA	ICD-10 CM	PAIN DUE TO INTERNAL ORTHOPEDIC PROSTHETIC DEVICES IMPLANTS AND GRAFTS INITIAL ENCOUNTER
Other Complication	T79.A22S	ICD-10 CM	TRAUMATIC COMPARTMENT SYNDROME OF LEFT LOWER EXTREMITY SEQUELA
Other Complication	M71.471	ICD-10 CM	CALCIUM DEPOSIT IN BURSA RIGHT ANKLE AND FOOT
Other Complication	M66.379	ICD-10 CM	SPONTANEOUS RUPTURE OF FLEXOR TENDONS UNSPECIFIED ANKLE AND FOOT
Other Complication	M88.872	ICD-10 CM	OSTEITIS DEFORMANS OF LEFT ANKLE AND FOOT
Other Complication	M85.472	ICD-10 CM	SOLITARY BONE CYST LEFT ANKLE AND FOOT
Other Complication	M62.271	ICD-10 CM	NONTRAUMATIC ISCHEMIC INFARCTION OF MUSCLE RIGHT ANKLE AND FOOT
Other Complication	M65.872	ICD-10 CM	OTHER SYNOVITIS AND TENOSYNOVITIS LEFT ANKLE AND FOOT
Other Complication	T84.81XD	ICD-10 CM	EMBOLISM DUE TO INTERNAL ORTHOPEDIC PROSTHETIC DEVICES IMPLANTS AND GRAFTS SUBSEQUENT ENCOUNTER
Other Complication	M96.89	ICD-10 CM	OTHER INTRAOPERATIVE AND POSTPROCEDURAL COMPLICATIONS AND DISORDERS OF THE MUSCULOSKELETAL SYSTEM
Other Complication	T84.82XD	ICD-10 CM	FIBROSIS DUE TO INTERNAL ORTHOPEDIC PROSTHETIC DEVICES IMPLANTS AND GRAFTS SUBSEQUENT ENCOUNTER
Other Complication	M77.51	ICD-10 CM	OTHER ENTHESOPATHY OF RIGHT FOOT
Other Complication	M90.872	ICD-10 CM	OSTEOPATHY IN DISEASES CLASSIFIED ELSEWHERE LEFT ANKLE AND FOOT
Other Complication	M60.171	ICD-10 CM	INTERSTITIAL MYOSITIS RIGHT ANKLE AND FOOT
Other Complication	M66.272	ICD-10 CM	SPONTANEOUS RUPTURE OF EXTENSOR TENDONS LEFT ANKLE AND FOOT
Other Complication	T84.85XA	ICD-10 CM	STENOSIS DUE TO INTERNAL ORTHOPEDIC PROSTHETIC DEVICES IMPLANTS AND GRAFTS INITIAL ENCOUNTER
Other Complication	T79.A22D	ICD-10 CM	TRAUMATIC COMPARTMENT SYNDROME OF LEFT LOWER EXTREMITY SUBSEQUENT ENCOUNTER
Other Complication	M85.671	ICD-10 CM	OTHER CYST OF BONE RIGHT ANKLE AND FOOT
Other Complication	M61.179	ICD-10 CM	MYOSITIS OSSIFICANS PROGRESSIVA UNSPECIFIED TOE(S)
Other Complication	M96.831	ICD-10 CM	POSTPROCEDURAL HEMORRHAGE AND HEMATOMA OF A MUSCULOSKELETAL STRUCTURE FOLLOWING OTHER PROCEDURE
Other Complication	M62.572	ICD-10 CM	MUSCLE WASTING AND ATROPHY NOT ELSEWHERE CLASSIFIED LEFT ANKLE AND FOOT
Other Complication	M62.171	ICD-10 CM	OTHER RUPTURE OF MUSCLE (NONTRAUMATIC) RIGHT ANKLE AND FOOT
Other Complication	M92.60	ICD-10 CM	JUVENILE OSTEOCHONDROSIS OF TARSUS UNSPECIFIED ANKLE
Other Complication	T84.85XD	ICD-10 CM	STENOSIS DUE TO INTERNAL ORTHOPEDIC PROSTHETIC DEVICES IMPLANTS AND GRAFTS SUBSEQUENT ENCOUNTER
Other Complication	M67.279	ICD-10 CM	SYNOVIAL HYPERTROPHY NOT ELSEWHERE CLASSIFIED UNSPECIFIED ANKLE AND FOOT
Other Complication	M60.272	ICD-10 CM	FOREIGN BODY GRANULOMA OF SOFT TISSUE NOT ELSEWHERE CLASSIFIED LEFT ANKLE AND FOOT
Other Complication	T84.81XA	ICD-10 CM	EMBOLISM DUE TO INTERNAL ORTHOPEDIC PROSTHETIC DEVICES IMPLANTS AND GRAFTS INITIAL ENCOUNTER
Other Complication	M66.372	ICD-10 CM	SPONTANEOUS RUPTURE OF FLEXOR TENDONS LEFT ANKLE AND FOOT
Other Complication	T79.A21S	ICD-10 CM	TRAUMATIC COMPARTMENT SYNDROME OF RIGHT LOWER EXTREMITY SEQUELA
Other Complication	M67.02	ICD-10 CM	SHORT ACHILLES TENDON (ACQUIRED) LEFT ANKLE
Other Complication	M70.879	ICD-10 CM	OTHER SOFT TISSUE DISORDERS RELATED TO USE OVERUSE AND PRESSURE UNSPECIFIED ANKLE AND FOOT
Other Complication	T84.84XD	ICD-10 CM	PAIN DUE TO INTERNAL ORTHOPEDIC PROSTHETIC DEVICES IMPLANTS AND GRAFTS SUBSEQUENT ENCOUNTER
Other Complication	M76.60	ICD-10 CM	ACHILLES TENDINITIS UNSPECIFIED LEG
Other Complication	M90.571	ICD-10 CM	OSTEONECROSIS IN DISEASES CLASSIFIED ELSEWHERE RIGHT ANKLE AND FOOT
Other Complication	M84.872	ICD-10 CM	OTHER DISORDERS OF CONTINUITY OF BONE LEFT ANKLE AND FOOT
Other Complication	M88.871	ICD-10 CM	OSTEITIS DEFORMANS OF RIGHT ANKLE AND FOOT
Other Complication	M62.179	ICD-10 CM	OTHER RUPTURE OF MUSCLE (NONTRAUMATIC) UNSPECIFIED ANKLE AND FOOT
Other Complication	M89.571	ICD-10 CM	OSTEOLYSIS RIGHT ANKLE AND FOOT
Other Complication	M89.572	ICD-10 CM	OSTEOLYSIS LEFT ANKLE AND FOOT
Other Complication	M67.471	ICD-10 CM	GANGLION RIGHT ANKLE AND FOOT
Other Complication	M70.872	ICD-10 CM	OTHER SOFT TISSUE DISORDERS RELATED TO USE OVERUSE AND PRESSURE LEFT ANKLE AND FOOT
Other Complication	M88.879	ICD-10 CM	OSTEITIS DEFORMANS OF UNSPECIFIED ANKLE AND FOOT
Other Complication	M60.071	ICD-10 CM	INFECTIVE MYOSITIS LEFT ANKLE
Other Complication	M67.879	ICD-10 CM	OTHER SPECIFIED DISORDERS OF SYNOVIUM AND TENDON UNSPECIFIED ANKLE AND FOOT
Other Complication	M61.372	ICD-10 CM	CALCIFICATION AND OSSIFICATION OF MUSCLES ASSOCIATED WITH BURNS LEFT ANKLE AND FOOT
Other Complication	M65.272	ICD-10 CM	CALCIFIC TENDINITIS LEFT ANKLE AND FOOT
Other Complication	M67.979	ICD-10 CM	UNSPECIFIED DISORDER OF SYNOVIUM AND TENDON UNSPECIFIED ANKLE AND FOOT
Other Complication	M70.979	ICD-10 CM	UNSPECIFIED SOFT TISSUE DISORDER RELATED TO USE OVERUSE AND PRESSURE UNSPECIFIED ANKLE AND FOOT

Other Complication	M63.872	ICD-10 CM	DISORDERS OF MUSCLE IN DISEASES CLASSIFIED ELSEWHERE LEFT ANKLE AND FOOT
Other Complication	M67.372	ICD-10 CM	TRANSIENT SYNOVITIS LEFT ANKLE AND FOOT
Other Complication	M85.371	ICD-10 CM	OSTEITIS CONDENSANS RIGHT ANKLE AND FOOT
Other Complication	M61.172	ICD-10 CM	MYOSITIS OSSIFICANS PROGRESSIVA LEFT ANKLE
Other Complication	M90.579	ICD-10 CM	OSTEONECROSIS IN DISEASES CLASSIFIED ELSEWHERE UNSPECIFIED ANKLE AND FOOT
Other Complication	M61.171	ICD-10 CM	MYOSITIS OSSIFICANS PROGRESSIVA RIGHT ANKLE
Other Complication	M62.079	ICD-10 CM	SEPARATION OF MUSCLE (NONTRAUMATIC) UNSPECIFIED ANKLE AND FOOT
Other Complication	M61.471	ICD-10 CM	OTHER CALCIFICATION OF MUSCLE RIGHT ANKLE AND FOOT
Other Complication	M84.879	ICD-10 CM	OTHER DISORDERS OF CONTINUITY OF BONE UNSPECIFIED ANKLE AND FOOT
Other Complication	M66.173	ICD-10 CM	RUPTURE OF SYNOVIUM UNSPECIFIED ANKLE
Other Complication	M67.874	ICD-10 CM	OTHER SPECIFIED DISORDERS OF TENDON LEFT ANKLE AND FOOT
Other Complication	M60.879	ICD-10 CM	OTHER MYOSITIS UNSPECIFIED ANKLE AND FOOT
Other Complication	M85.879	ICD-10 CM	OTHER SPECIFIED DISORDERS OF BONE DENSITY AND STRUCTURE UNSPECIFIED ANKLE AND FOOT
Other Complication	M93.971	ICD-10 CM	OSTEOCHONDROPATHY UNSPECIFIED RIGHT ANKLE AND FOOT
Other Complication	M96.811	ICD-10 CM	INTRAOPERATIVE HEMORRHAGE AND HEMATOMA OF A MUSCULOSKELETAL STRUCTURE COMPLICATING OTHER PROCEDURE
Other Complication	M94.272	ICD-10 CM	CHONDROMALACIA LEFT ANKLE AND JOINTS OF LEFT FOOT
Other Complication	M67.271	ICD-10 CM	SYNOVIAL HYPERTROPHY NOT ELSEWHERE CLASSIFIED RIGHT ANKLE AND FOOT
Other Complication	M71.571	ICD-10 CM	OTHER BURSITIS NOT ELSEWHERE CLASSIFIED RIGHT ANKLE AND FOOT
Other Complication	M70.972	ICD-10 CM	UNSPECIFIED SOFT TISSUE DISORDER RELATED TO USE OVERUSE AND PRESSURE LEFT ANKLE AND FOOT
Other Complication	M77.52	ICD-10 CM	OTHER ENTHESOPATHY OF LEFT FOOT
Other Complication	M66.171	ICD-10 CM	RUPTURE OF SYNOVIUM RIGHT ANKLE
Other Complication	M66.371	ICD-10 CM	SPONTANEOUS RUPTURE OF FLEXOR TENDONS RIGHT ANKLE AND FOOT
Other Complication	M60.172	ICD-10 CM	INTERSTITIAL MYOSITIS LEFT ANKLE AND FOOT
Other Complication	M66.172	ICD-10 CM	RUPTURE OF SYNOVIUM LEFT ANKLE
Other Complication	M61.379	ICD-10 CM	CALCIFICATION AND OSSIFICATION OF MUSCLES ASSOCIATED WITH BURNS UNSPECIFIED ANKLE AND FOOT
Other Complication	M84.871	ICD-10 CM	OTHER DISORDERS OF CONTINUITY OF BONE RIGHT ANKLE AND FOOT
Other Complication	M96.810	ICD-10 CM	INTRAOPERATIVE HEMORRHAGE AND HEMATOMA OF A MUSCULOSKELETAL STRUCTURE COMPLICATING A MUSCULOSKELETAL SYSTEM PROCEDURE
Other Complication	M62.579	ICD-10 CM	MUSCLE WASTING AND ATROPHY NOT ELSEWHERE CLASSIFIED UNSPECIFIED ANKLE AND FOOT
Other Complication	M90.671	ICD-10 CM	OSTEITIS DEFORMANS IN NEOPLASTIC DISEASES RIGHT ANKLE AND FOOT
Other Complication	M92.62	ICD-10 CM	JUVENILE OSTEOCHONDROSIS OF TARSUS LEFT ANKLE
Other Complication	T84.84XS	ICD-10 CM	PAIN DUE TO INTERNAL ORTHOPEDIC PROSTHETIC DEVICES IMPLANTS AND GRAFTS SEQUELA
Other Complication	M85.572	ICD-10 CM	ANEURYSMAL BONE CYST LEFT ANKLE AND FOOT
Other Complication	M60.872	ICD-10 CM	OTHER MYOSITIS LEFT ANKLE AND FOOT
Other Complication	T79.A21D	ICD-10 CM	TRAUMATIC COMPARTMENT SYNDROME OF RIGHT LOWER EXTREMITY SUBSEQUENT ENCOUNTER
Other Complication	M63.871	ICD-10 CM	DISORDERS OF MUSCLE IN DISEASES CLASSIFIED ELSEWHERE RIGHT ANKLE AND FOOT
Other Complication	M89.071	ICD-10 CM	ALGONEURODYSTROPHY RIGHT ANKLE AND FOOT
Other Complication	M89.8X7	ICD-10 CM	OTHER SPECIFIED DISORDERS OF BONE ANKLE AND FOOT
Other Complication	M67.479	ICD-10 CM	GANGLION UNSPECIFIED ANKLE AND FOOT
Other Complication	M62.072	ICD-10 CM	SEPARATION OF MUSCLE (NONTRAUMATIC) LEFT ANKLE AND FOOT
Other Complication	M60.271	ICD-10 CM	FOREIGN BODY GRANULOMA OF SOFT TISSUE NOT ELSEWHERE CLASSIFIED RIGHT ANKLE AND FOOT
Other Complication	M85.379	ICD-10 CM	OSTEITIS CONDENSANS UNSPECIFIED ANKLE AND FOOT
Other Complication	M94.279	ICD-10 CM	CHONDROMALACIA UNSPECIFIED ANKLE AND JOINTS OF FOOT
Other Complication	M70.971	ICD-10 CM	UNSPECIFIED SOFT TISSUE DISORDER RELATED TO USE OVERUSE AND PRESSURE RIGHT ANKLE AND FOOT
Other Complication	M63.879	ICD-10 CM	DISORDERS OF MUSCLE IN DISEASES CLASSIFIED ELSEWHERE UNSPECIFIED ANKLE AND FOOT
Other Complication	M70.871	ICD-10 CM	OTHER SOFT TISSUE DISORDERS RELATED TO USE OVERUSE AND PRESSURE RIGHT ANKLE AND FOOT
Other Complication	M93.871	ICD-10 CM	OTHER SPECIFIED OSTEOCHONDROPATHIES RIGHT ANKLE AND FOOT
Other Complication	M90.672	ICD-10 CM	OSTEITIS DEFORMANS IN NEOPLASTIC DISEASES LEFT ANKLE AND FOOT
Other Complication	M65.271	ICD-10 CM	CALCIFIC TENDINITIS RIGHT ANKLE AND FOOT
Other Complication	M89.772	ICD-10 CM	MAJOR OSSEOUS DEFECT LEFT ANKLE AND FOOT
Other Complication	M65.871	ICD-10 CM	OTHER SYNOVITIS AND TENOSYNOVITIS RIGHT ANKLE AND FOOT
Other Complication	M60.179	ICD-10 CM	INTERSTITIAL MYOSITIS UNSPECIFIED ANKLE AND FOOT
Other Complication	M96.821	ICD-10 CM	ACCIDENTAL PUNCTURE AND LACERATION OF A MUSCULOSKELETAL STRUCTURE DURING OTHER PROCEDURE
Other Complication	M92.61	ICD-10 CM	JUVENILE OSTEOCHONDROSIS OF TARSUS RIGHT ANKLE
Other Complication	M85.479	ICD-10 CM	SOLITARY BONE CYST UNSPECIFIED ANKLE AND FOOT
Other Complication	M85.872	ICD-10 CM	OTHER SPECIFIED DISORDERS OF BONE DENSITY AND STRUCTURE LEFT ANKLE AND FOOT
Other Complication	T79.A22A	ICD-10 CM	TRAUMATIC COMPARTMENT SYNDROME OF LEFT LOWER EXTREMITY INITIAL ENCOUNTER
Other Complication	M90.871	ICD-10 CM	OSTEOPATHY IN DISEASES CLASSIFIED ELSEWHERE RIGHT ANKLE AND FOOT
Other Complication	M93.879	ICD-10 CM	OTHER SPECIFIED OSTEOCHONDROPATHIES UNSPECIFIED ANKLE AND FOOT
Other Complication	M85.672	ICD-10 CM	OTHER CYST OF BONE LEFT ANKLE AND FOOT

Other Complication	M61.279	ICD-10 CM	PARALYTIC CALCIFICATION AND OSSIFICATION OF MUSCLE UNSPECIFIED ANKLE AND FOOT
Other Complication	M66.271	ICD-10 CM	SPONTANEOUS RUPTURE OF EXTENSOR TENDONS RIGHT ANKLE AND FOOT
Other Complication	M65.879	ICD-10 CM	OTHER SYNOVITIS AND TENOSYNOVITIS UNSPECIFIED ANKLE AND FOOT
Other Complication	M67.472	ICD-10 CM	GANGLION LEFT ANKLE AND FOOT
Other Complication	M67.01	ICD-10 CM	SHORT ACHILLES TENDON (ACQUIRED) RIGHT ANKLE
Other Complication	M67.971	ICD-10 CM	UNSPECIFIED DISORDER OF SYNOVIUM AND TENDON RIGHT ANKLE AND FOOT
Other Complication	M77.50	ICD-10 CM	OTHER ENTHESOPATHY OF UNSPECIFIED FOOT
Other Complication	M71.872	ICD-10 CM	OTHER SPECIFIED BURSOPATHIES LEFT ANKLE AND FOOT
Other Complication	M90.679	ICD-10 CM	OSTEITIS DEFORMANS IN NEOPLASTIC DISEASES UNSPECIFIED ANKLE AND FOOT
Other Complication	T79.A21A	ICD-10 CM	TRAUMATIC COMPARTMENT SYNDROME OF RIGHT LOWER EXTREMITY INITIAL ENCOUNTER
Other Complication	M60.072	ICD-10 CM	INFECTIVE MYOSITIS UNSPECIFIED ANKLE
Other Complication	M89.779	ICD-10 CM	MAJOR OSSEOUS DEFECT UNSPECIFIED ANKLE AND FOOT
Other Complication	M62.571	ICD-10 CM	MUSCLE WASTING AND ATROPHY NOT ELSEWHERE CLASSIFIED RIGHT ANKLE AND FOOT
Other Complication	M62.479	ICD-10 CM	CONTRACTURE OF MUSCLE UNSPECIFIED ANKLE AND FOOT
Other Complication	M85.171	ICD-10 CM	SKELETAL FLUOROSIS RIGHT ANKLE AND FOOT
Other Complication	M61.072	ICD-10 CM	MYOSITIS OSSIFICANS TRAUMATICA LEFT ANKLE AND FOOT
Other Complication	M61.579	ICD-10 CM	OTHER OSSIFICATION OF MUSCLE UNSPECIFIED ANKLE AND FOOT
Other Complication	M71.371	ICD-10 CM	OTHER BURSAL CYST RIGHT ANKLE AND FOOT
Other Complication	M61.371	ICD-10 CM	CALCIFICATION AND OSSIFICATION OF MUSCLES ASSOCIATED WITH BURNS RIGHT ANKLE AND FOOT
Other Complication	M62.279	ICD-10 CM	NONTRAUMATIC ISCHEMIC INFARCTION OF MUSCLE UNSPECIFIED ANKLE AND FOOT
Other Complication	T84.85XS	ICD-10 CM	STENOSIS DUE TO INTERNAL ORTHOPEDIC PROSTHETIC DEVICES IMPLANTS AND GRAFTS SEQUELA
Other Complication	M89.072	ICD-10 CM	ALGONEURODYSTROPHY LEFT ANKLE AND FOOT
Other Complication	M67.379	ICD-10 CM	TRANSIENT SYNOVITIS UNSPECIFIED ANKLE AND FOOT
Other Complication	M85.571	ICD-10 CM	ANEURYSMAL BONE CYST RIGHT ANKLE AND FOOT
Other Complication	M96.830	ICD-10 CM	POSTPROCEDURAL HEMORRHAGE AND HEMATOMA OF A MUSCULOSKELETAL STRUCTURE FOLLOWING A MUSCULOSKELETAL SYSTEM PROCEDURE
Other Complication	M66.279	ICD-10 CM	SPONTANEOUS RUPTURE OF EXTENSOR TENDONS UNSPECIFIED ANKLE AND FOOT
Other Complication	T84.89XS	ICD-10 CM	OTHER SPECIFIED COMPLICATION OF INTERNAL ORTHOPEDIC PROSTHETIC DEVICES IMPLANTS AND GRAFTS SEQUELA
Other Complication	T79.A29S	ICD-10 CM	TRAUMATIC COMPARTMENT SYNDROME OF UNSPECIFIED LOWER EXTREMITY SEQUELA
Other Complication	M71.879	ICD-10 CM	OTHER SPECIFIED BURSOPATHIES UNSPECIFIED ANKLE AND FOOT
Other Complication	T84.83XS	ICD-10 CM	HEMORRHAGE DUE TO INTERNAL ORTHOPEDIC PROSTHETIC DEVICES IMPLANTS AND GRAFTS SEQUELA
Other Complication	M89.771	ICD-10 CM	MAJOR OSSEOUS DEFECT RIGHT ANKLE AND FOOT
Other Complication	M67.972	ICD-10 CM	UNSPECIFIED DISORDER OF SYNOVIUM AND TENDON LEFT ANKLE AND FOOT
Other Complication	M85.071	ICD-10 CM	FIBROUS DYSPLASIA (MONOSTOTIC) RIGHT ANKLE AND FOOT
Other Complication	M76.61	ICD-10 CM	ACHILLES TENDINITIS RIGHT LEG
Other Complication	M76.62	ICD-10 CM	ACHILLES TENDINITIS LEFT LEG
Other Complication	M62.471	ICD-10 CM	CONTRACTURE OF MUSCLE RIGHT ANKLE AND FOOT
Other Complication	T84.89XD	ICD-10 CM	OTHER SPECIFIED COMPLICATION OF INTERNAL ORTHOPEDIC PROSTHETIC DEVICES IMPLANTS AND GRAFTS SUBSEQUENT ENCOUNTER
Other Complication	M85.372	ICD-10 CM	OSTEITIS CONDENSANS LEFT ANKLE AND FOOT
Other Complication	M71.579	ICD-10 CM	OTHER BURSITIS NOT ELSEWHERE CLASSIFIED UNSPECIFIED ANKLE AND FOOT
Other Complication	M93.271	ICD-10 CM	OSTEOCHONDRITIS DISSECANS RIGHT ANKLE AND JOINTS OF RIGHT FOOT
Other Complication	T84.86XA	ICD-10 CM	THROMBOSIS DUE TO INTERNAL ORTHOPEDIC PROSTHETIC DEVICES IMPLANTS AND GRAFTS INITIAL ENCOUNTER
Other Complication	M67.873	ICD-10 CM	OTHER SPECIFIED DISORDERS OF TENDON RIGHT ANKLE AND FOOT
Other Complication	M71.479	ICD-10 CM	CALCIUM DEPOSIT IN BURSA UNSPECIFIED ANKLE AND FOOT
Other Complication	M62.272	ICD-10 CM	NONTRAUMATIC ISCHEMIC INFARCTION OF MUSCLE LEFT ANKLE AND FOOT
Other Complication	M85.471	ICD-10 CM	SOLITARY BONE CYST RIGHT ANKLE AND FOOT
Other Complication	M85.579	ICD-10 CM	ANEURYSMAL BONE CYST UNSPECIFIED ANKLE AND FOOT
Other Complication	M85.172	ICD-10 CM	SKELETAL FLUOROSIS LEFT ANKLE AND FOOT
Other Complication	M62.071	ICD-10 CM	SEPARATION OF MUSCLE (NONTRAUMATIC) RIGHT ANKLE AND FOOT
Other Complication	M85.072	ICD-10 CM	FIBROUS DYSPLASIA (MONOSTOTIC) LEFT ANKLE AND FOOT
Other Complication	M96.820	ICD-10 CM	ACCIDENTAL PUNCTURE AND LACERATION OF A MUSCULOSKELETAL STRUCTURE DURING A MUSCULOSKELETAL SYSTEM PROCEDURE
Pain	M79.662	ICD-10 CM	PAIN IN LEFT LOWER LEG
Pain	M25.571	ICD-10 CM	PAIN IN RIGHT ANKLE AND JOINTS OF RIGHT FOOT
Pain	G89.11	ICD-10 CM	ACUTE PAIN DUE TO TRAUMA
Pain	M25.579	ICD-10 CM	PAIN IN UNSPECIFIED ANKLE AND JOINTS OF UNSPECIFIED FOOT
Pain	G89.29	ICD-10 CM	OTHER CHRONIC PAIN
Pain	G89.22	ICD-10 CM	CHRONIC POST-THORACOTOMY PAIN
Pain	M25.572	ICD-10 CM	PAIN IN LEFT ANKLE AND JOINTS OF LEFT FOOT
Pain	M79.669	ICD-10 CM	PAIN IN UNSPECIFIED LOWER LEG
Pain	G89.18	ICD-10 CM	OTHER ACUTE POSTPROCEDURAL PAIN
Pain	M79.604	ICD-10 CM	PAIN IN RIGHT LEG
Pain	M79.606	ICD-10 CM	PAIN IN LEG UNSPECIFIED

Pain	M79.605	ICD-10 CM	PAIN IN LEFT LEG
Pain	G89.12	ICD-10 CM	ACUTE POST-THORACOTOMY PAIN
Pain	G89.28	ICD-10 CM	OTHER CHRONIC POSTPROCEDURAL PAIN
Pain	M79.661	ICD-10 CM	PAIN IN RIGHT LOWER LEG
Pain	G89.21	ICD-10 CM	CHRONIC PAIN DUE TO TRAUMA
Refracture	M84.573G	ICD-10 CM	PATHOLOGICAL FRACTURE IN NEOPLASTIC DISEASE UNSPECIFIED ANKLE SUBSEQUENT ENCOUNTER FOR FRACTURE WITH DELAYED HEALING
Refracture	M84.671P	ICD-10 CM	PATHOLOGICAL FRACTURE IN OTHER DISEASE RIGHT ANKLE SUBSEQUENT ENCOUNTER FOR FRACTURE WITH MALUNION
Refracture	M84.572S	ICD-10 CM	PATHOLOGICAL FRACTURE IN NEOPLASTIC DISEASE LEFT ANKLE SEQUELA
Refracture	M84.673S	ICD-10 CM	PATHOLOGICAL FRACTURE IN OTHER DISEASE UNSPECIFIED ANKLE SEQUELA
Refracture	M84.471G	ICD-10 CM	PATHOLOGICAL FRACTURE RIGHT ANKLE SUBSEQUENT ENCOUNTER FOR FRACTURE WITH DELAYED HEALING
Refracture	M84.372D	ICD-10 CM	STRESS FRACTURE LEFT ANKLE SUBSEQUENT ENCOUNTER FOR FRACTURE WITH ROUTINE HEALING
Refracture	M84.673D	ICD-10 CM	PATHOLOGICAL FRACTURE IN OTHER DISEASE UNSPECIFIED ANKLE SUBSEQUENT ENCOUNTER FOR FRACTURE WITH ROUTINE HEALING
Refracture	M84.471A	ICD-10 CM	PATHOLOGICAL FRACTURE RIGHT ANKLE INITIAL ENCOUNTER FOR FRACTURE
Refracture	M84.371A	ICD-10 CM	STRESS FRACTURE RIGHT ANKLE INITIAL ENCOUNTER FOR FRACTURE
Refracture	M84.372P	ICD-10 CM	STRESS FRACTURE LEFT ANKLE SUBSEQUENT ENCOUNTER FOR FRACTURE WITH MALUNION
Refracture	M84.473K	ICD-10 CM	PATHOLOGICAL FRACTURE UNSPECIFIED ANKLE SUBSEQUENT ENCOUNTER FOR FRACTURE WITH NONUNION
Refracture	M80.872K	ICD-10 CM	OTHER OSTEOPOROSIS WITH CURRENT PATHOLOGICAL FRACTURE LEFT ANKLE AND FOOT SUBSEQUENT ENCOUNTER FOR FRACTURE WITH NONUNION
Refracture	M84.671A	ICD-10 CM	PATHOLOGICAL FRACTURE IN OTHER DISEASE RIGHT ANKLE INITIAL ENCOUNTER FOR FRACTURE
Refracture	M80.071A	ICD-10 CM	AGE-RELATED OSTEOPOROSIS WITH CURRENT PATHOLOGICAL FRACTURE RIGHT ANKLE AND FOOT INITIAL ENCOUNTER FOR FRACTURE
Refracture	M84.372S	ICD-10 CM	STRESS FRACTURE LEFT ANKLE SEQUELA
Refracture	M80.079P	ICD-10 CM	AGE-RELATED OSTEOPOROSIS WITH CURRENT PATHOLOGICAL FRACTURE UNSPECIFIED ANKLE AND FOOT SUBSEQUENT ENCOUNTER FOR FRACTURE WITH MALUNION
Refracture	M80.879P	ICD-10 CM	OTHER OSTEOPOROSIS WITH CURRENT PATHOLOGICAL FRACTURE UNSPECIFIED ANKLE AND FOOT SUBSEQUENT ENCOUNTER FOR FRACTURE WITH MALUNION
Refracture	M84.473D	ICD-10 CM	PATHOLOGICAL FRACTURE UNSPECIFIED ANKLE SUBSEQUENT ENCOUNTER FOR FRACTURE WITH ROUTINE HEALING
Refracture	M84.573P	ICD-10 CM	PATHOLOGICAL FRACTURE IN NEOPLASTIC DISEASE UNSPECIFIED ANKLE SUBSEQUENT ENCOUNTER FOR FRACTURE WITH MALUNION
Refracture	M80.879K	ICD-10 CM	OTHER OSTEOPOROSIS WITH CURRENT PATHOLOGICAL FRACTURE UNSPECIFIED ANKLE AND FOOT SUBSEQUENT ENCOUNTER FOR FRACTURE WITH NONUNION
Refracture	M80.872P	ICD-10 CM	OTHER OSTEOPOROSIS WITH CURRENT PATHOLOGICAL FRACTURE LEFT ANKLE AND FOOT SUBSEQUENT ENCOUNTER FOR FRACTURE WITH MALUNION
Refracture	M84.671D	ICD-10 CM	PATHOLOGICAL FRACTURE IN OTHER DISEASE RIGHT ANKLE SUBSEQUENT ENCOUNTER FOR FRACTURE WITH ROUTINE HEALING
Refracture	M84.471S	ICD-10 CM	PATHOLOGICAL FRACTURE RIGHT ANKLE SEQUELA
Refracture	M84.373G	ICD-10 CM	STRESS FRACTURE UNSPECIFIED ANKLE SUBSEQUENT ENCOUNTER FOR FRACTURE WITH DELAYED HEALING
Refracture	M80.872G	ICD-10 CM	OTHER OSTEOPOROSIS WITH CURRENT PATHOLOGICAL FRACTURE LEFT ANKLE AND FOOT SUBSEQUENT ENCOUNTER FOR FRACTURE WITH DELAYED HEALING
Refracture	M84.472P	ICD-10 CM	PATHOLOGICAL FRACTURE LEFT ANKLE SUBSEQUENT ENCOUNTER FOR FRACTURE WITH MALUNION
Refracture	M84.572K	ICD-10 CM	PATHOLOGICAL FRACTURE IN NEOPLASTIC DISEASE LEFT ANKLE SUBSEQUENT ENCOUNTER FOR FRACTURE WITH NONUNION
Refracture	M80.871D	ICD-10 CM	OTHER OSTEOPOROSIS WITH CURRENT PATHOLOGICAL FRACTURE RIGHT ANKLE AND FOOT SUBSEQUENT ENCOUNTER FOR FRACTURE WITH ROUTINE HEALING
Refracture	M84.573S	ICD-10 CM	PATHOLOGICAL FRACTURE IN NEOPLASTIC DISEASE UNSPECIFIED ANKLE SEQUELA
Refracture	M84.571D	ICD-10 CM	PATHOLOGICAL FRACTURE IN NEOPLASTIC DISEASE RIGHT ANKLE SUBSEQUENT ENCOUNTER FOR FRACTURE WITH ROUTINE HEALING
Refracture	M84.472S	ICD-10 CM	PATHOLOGICAL FRACTURE LEFT ANKLE SEQUELA
Refracture	M84.371D	ICD-10 CM	STRESS FRACTURE RIGHT ANKLE SUBSEQUENT ENCOUNTER FOR FRACTURE WITH ROUTINE HEALING
Refracture	M84.672G	ICD-10 CM	PATHOLOGICAL FRACTURE IN OTHER DISEASE LEFT ANKLE SUBSEQUENT ENCOUNTER FOR FRACTURE WITH DELAYED HEALING
Refracture	M80.872A	ICD-10 CM	OTHER OSTEOPOROSIS WITH CURRENT PATHOLOGICAL FRACTURE LEFT ANKLE AND FOOT INITIAL ENCOUNTER FOR FRACTURE
Refracture	M84.473P	ICD-10 CM	PATHOLOGICAL FRACTURE UNSPECIFIED ANKLE SUBSEQUENT ENCOUNTER FOR FRACTURE WITH MALUNION
Refracture	M84.571K	ICD-10 CM	PATHOLOGICAL FRACTURE IN NEOPLASTIC DISEASE RIGHT ANKLE SUBSEQUENT ENCOUNTER FOR FRACTURE WITH NONUNION

Refracture	M84.672A	ICD-10 CM	PATHOLOGICAL FRACTURE IN OTHER DISEASE LEFT ANKLE INITIAL ENCOUNTER FOR FRACTURE
Refracture	M84.373P	ICD-10 CM	STRESS FRACTURE UNSPECIFIED ANKLE SUBSEQUENT ENCOUNTER FOR FRACTURE WITH MALUNION
Refracture	M84.672P	ICD-10 CM	PATHOLOGICAL FRACTURE IN OTHER DISEASE LEFT ANKLE SUBSEQUENT ENCOUNTER FOR FRACTURE WITH MALUNION
Refracture	M84.372K	ICD-10 CM	STRESS FRACTURE LEFT ANKLE SUBSEQUENT ENCOUNTER FOR FRACTURE WITH NONUNION
Refracture	M84.671K	ICD-10 CM	PATHOLOGICAL FRACTURE IN OTHER DISEASE RIGHT ANKLE SUBSEQUENT ENCOUNTER FOR FRACTURE WITH NONUNION
Refracture	M84.572A	ICD-10 CM	PATHOLOGICAL FRACTURE IN NEOPLASTIC DISEASE LEFT ANKLE INITIAL ENCOUNTER FOR FRACTURE
Refracture	M84.372G	ICD-10 CM	STRESS FRACTURE LEFT ANKLE SUBSEQUENT ENCOUNTER FOR FRACTURE WITH DELAYED HEALING
Refracture	M84.571P	ICD-10 CM	PATHOLOGICAL FRACTURE IN NEOPLASTIC DISEASE RIGHT ANKLE SUBSEQUENT ENCOUNTER FOR FRACTURE WITH MALUNION
Refracture	M80.872S	ICD-10 CM	OTHER OSTEOPOROSIS WITH CURRENT PATHOLOGICAL FRACTURE LEFT ANKLE AND FOOT SEQUELA
Refracture	M84.472A	ICD-10 CM	PATHOLOGICAL FRACTURE LEFT ANKLE INITIAL ENCOUNTER FOR FRACTURE
Refracture	M80.879G	ICD-10 CM	OTHER OSTEOPOROSIS WITH CURRENT PATHOLOGICAL FRACTURE UNSPECIFIED ANKLE AND FOOT SUBSEQUENT ENCOUNTER FOR FRACTURE WITH DELAYED HEALING
Refracture	M80.071K	ICD-10 CM	AGE-RELATED OSTEOPOROSIS WITH CURRENT PATHOLOGICAL FRACTURE RIGHT ANKLE AND FOOT SUBSEQUENT ENCOUNTER FOR FRACTURE WITH NONUNION
Refracture	M80.071D	ICD-10 CM	AGE-RELATED OSTEOPOROSIS WITH CURRENT PATHOLOGICAL FRACTURE RIGHT ANKLE AND FOOT SUBSEQUENT ENCOUNTER FOR FRACTURE WITH ROUTINE HEALING
Refracture	M80.079A	ICD-10 CM	AGE-RELATED OSTEOPOROSIS WITH CURRENT PATHOLOGICAL FRACTURE UNSPECIFIED ANKLE AND FOOT INITIAL ENCOUNTER FOR FRACTURE
Refracture	M84.673A	ICD-10 CM	PATHOLOGICAL FRACTURE IN OTHER DISEASE UNSPECIFIED ANKLE INITIAL ENCOUNTER FOR FRACTURE
Refracture	M80.879A	ICD-10 CM	OTHER OSTEOPOROSIS WITH CURRENT PATHOLOGICAL FRACTURE UNSPECIFIED ANKLE AND FOOT INITIAL ENCOUNTER FOR FRACTURE
Refracture	M84.572G	ICD-10 CM	PATHOLOGICAL FRACTURE IN NEOPLASTIC DISEASE LEFT ANKLE SUBSEQUENT ENCOUNTER FOR FRACTURE WITH DELAYED HEALING
Refracture	M80.072A	ICD-10 CM	AGE-RELATED OSTEOPOROSIS WITH CURRENT PATHOLOGICAL FRACTURE LEFT ANKLE AND FOOT INITIAL ENCOUNTER FOR FRACTURE
Refracture	M84.673P	ICD-10 CM	PATHOLOGICAL FRACTURE IN OTHER DISEASE UNSPECIFIED ANKLE SUBSEQUENT ENCOUNTER FOR FRACTURE WITH MALUNION
Refracture	M80.871G	ICD-10 CM	OTHER OSTEOPOROSIS WITH CURRENT PATHOLOGICAL FRACTURE RIGHT ANKLE AND FOOT SUBSEQUENT ENCOUNTER FOR FRACTURE WITH DELAYED HEALING
Refracture	M84.471D	ICD-10 CM	PATHOLOGICAL FRACTURE RIGHT ANKLE SUBSEQUENT ENCOUNTER FOR FRACTURE WITH ROUTINE HEALING
Refracture	M80.879S	ICD-10 CM	OTHER OSTEOPOROSIS WITH CURRENT PATHOLOGICAL FRACTURE UNSPECIFIED ANKLE AND FOOT SEQUELA
Refracture	M84.371P	ICD-10 CM	STRESS FRACTURE RIGHT ANKLE SUBSEQUENT ENCOUNTER FOR FRACTURE WITH MALUNION
Refracture	M84.372A	ICD-10 CM	STRESS FRACTURE LEFT ANKLE INITIAL ENCOUNTER FOR FRACTURE
Refracture	M80.879D	ICD-10 CM	OTHER OSTEOPOROSIS WITH CURRENT PATHOLOGICAL FRACTURE UNSPECIFIED ANKLE AND FOOT SUBSEQUENT ENCOUNTER FOR FRACTURE WITH ROUTINE HEALING
Refracture	M84.672S	ICD-10 CM	PATHOLOGICAL FRACTURE IN OTHER DISEASE LEFT ANKLE SEQUELA
Refracture	M84.472G	ICD-10 CM	PATHOLOGICAL FRACTURE LEFT ANKLE SUBSEQUENT ENCOUNTER FOR FRACTURE WITH DELAYED HEALING
Refracture	M80.871S	ICD-10 CM	OTHER OSTEOPOROSIS WITH CURRENT PATHOLOGICAL FRACTURE RIGHT ANKLE AND FOOT SEQUELA
Refracture	M80.079D	ICD-10 CM	AGE-RELATED OSTEOPOROSIS WITH CURRENT PATHOLOGICAL FRACTURE UNSPECIFIED ANKLE AND FOOT SUBSEQUENT ENCOUNTER FOR FRACTURE WITH ROUTINE HEALING
Refracture	M84.473S	ICD-10 CM	PATHOLOGICAL FRACTURE UNSPECIFIED ANKLE SEQUELA
Refracture	M84.471K	ICD-10 CM	PATHOLOGICAL FRACTURE RIGHT ANKLE SUBSEQUENT ENCOUNTER FOR FRACTURE WITH NONUNION
Refracture	M80.072D	ICD-10 CM	AGE-RELATED OSTEOPOROSIS WITH CURRENT PATHOLOGICAL FRACTURE LEFT ANKLE AND FOOT SUBSEQUENT ENCOUNTER FOR FRACTURE WITH ROUTINE HEALING
Refracture	M84.573A	ICD-10 CM	PATHOLOGICAL FRACTURE IN NEOPLASTIC DISEASE UNSPECIFIED ANKLE INITIAL ENCOUNTER FOR FRACTURE
Refracture	M80.079K	ICD-10 CM	AGE-RELATED OSTEOPOROSIS WITH CURRENT PATHOLOGICAL FRACTURE UNSPECIFIED ANKLE AND FOOT SUBSEQUENT ENCOUNTER FOR FRACTURE WITH NONUNION
Refracture	M84.373S	ICD-10 CM	STRESS FRACTURE UNSPECIFIED ANKLE SEQUELA
Refracture	M84.371K	ICD-10 CM	STRESS FRACTURE RIGHT ANKLE SUBSEQUENT ENCOUNTER FOR FRACTURE WITH NONUNION
Refracture	M84.373K	ICD-10 CM	STRESS FRACTURE UNSPECIFIED ANKLE SUBSEQUENT ENCOUNTER FOR FRACTURE WITH NONUNION
Refracture	M84.571S	ICD-10 CM	PATHOLOGICAL FRACTURE IN NEOPLASTIC DISEASE RIGHT ANKLE SEQUELA
Refracture	M84.472K	ICD-10 CM	PATHOLOGICAL FRACTURE LEFT ANKLE SUBSEQUENT ENCOUNTER FOR FRACTURE WITH NONUNION

Refracture	M84.571A	ICD-10 CM	PATHOLOGICAL FRACTURE IN NEOPLASTIC DISEASE RIGHT ANKLE INITIAL ENCOUNTER FOR FRACTURE
Refracture	M80.871A	ICD-10 CM	OTHER OSTEOPOROSIS WITH CURRENT PATHOLOGICAL FRACTURE RIGHT ANKLE AND FOOT INITIAL ENCOUNTER FOR FRACTURE
Refracture	M80.071G	ICD-10 CM	AGE-RELATED OSTEOPOROSIS WITH CURRENT PATHOLOGICAL FRACTURE RIGHT ANKLE AND FOOT SUBSEQUENT ENCOUNTER FOR FRACTURE WITH DELAYED HEALING
Refracture	M84.673G	ICD-10 CM	PATHOLOGICAL FRACTURE IN OTHER DISEASE UNSPECIFIED ANKLE SUBSEQUENT ENCOUNTER FOR FRACTURE WITH DELAYED HEALING
Refracture	M84.672D	ICD-10 CM	PATHOLOGICAL FRACTURE IN OTHER DISEASE LEFT ANKLE SUBSEQUENT ENCOUNTER FOR FRACTURE WITH ROUTINE HEALING
Refracture	M80.072P	ICD-10 CM	AGE-RELATED OSTEOPOROSIS WITH CURRENT PATHOLOGICAL FRACTURE LEFT ANKLE AND FOOT SUBSEQUENT ENCOUNTER FOR FRACTURE WITH MALUNION
Refracture	M84.471P	ICD-10 CM	PATHOLOGICAL FRACTURE RIGHT ANKLE SUBSEQUENT ENCOUNTER FOR FRACTURE WITH MALUNION
Refracture	M80.072K	ICD-10 CM	AGE-RELATED OSTEOPOROSIS WITH CURRENT PATHOLOGICAL FRACTURE LEFT ANKLE AND FOOT SUBSEQUENT ENCOUNTER FOR FRACTURE WITH NONUNION
Refracture	M84.572D	ICD-10 CM	PATHOLOGICAL FRACTURE IN NEOPLASTIC DISEASE LEFT ANKLE SUBSEQUENT ENCOUNTER FOR FRACTURE WITH ROUTINE HEALING
Refracture	M80.079G	ICD-10 CM	AGE-RELATED OSTEOPOROSIS WITH CURRENT PATHOLOGICAL FRACTURE UNSPECIFIED ANKLE AND FOOT SUBSEQUENT ENCOUNTER FOR FRACTURE WITH DELAYED HEALING
Refracture	M80.872D	ICD-10 CM	OTHER OSTEOPOROSIS WITH CURRENT PATHOLOGICAL FRACTURE LEFT ANKLE AND FOOT SUBSEQUENT ENCOUNTER FOR FRACTURE WITH ROUTINE HEALING
Refracture	M96.672	ICD-10 CM	FRACTURE OF TIBIA OR FIBULA FOLLOWING INSERTION OF ORTHOPEDIC IMPLANT JOINT PROSTHESIS OR BONE PLATE LEFT LEG
Refracture	M84.671G	ICD-10 CM	PATHOLOGICAL FRACTURE IN OTHER DISEASE RIGHT ANKLE SUBSEQUENT ENCOUNTER FOR FRACTURE WITH DELAYED HEALING
Refracture	M84.573K	ICD-10 CM	PATHOLOGICAL FRACTURE IN NEOPLASTIC DISEASE UNSPECIFIED ANKLE SUBSEQUENT ENCOUNTER FOR FRACTURE WITH NONUNION
Refracture	M84.473A	ICD-10 CM	PATHOLOGICAL FRACTURE UNSPECIFIED ANKLE INITIAL ENCOUNTER FOR FRACTURE
Refracture	M84.672K	ICD-10 CM	PATHOLOGICAL FRACTURE IN OTHER DISEASE LEFT ANKLE SUBSEQUENT ENCOUNTER FOR FRACTURE WITH NONUNION
Refracture	M80.079S	ICD-10 CM	AGE-RELATED OSTEOPOROSIS WITH CURRENT PATHOLOGICAL FRACTURE UNSPECIFIED ANKLE AND FOOT SEQUELA
Refracture	M80.071S	ICD-10 CM	AGE-RELATED OSTEOPOROSIS WITH CURRENT PATHOLOGICAL FRACTURE RIGHT ANKLE AND FOOT SEQUELA
Refracture	M80.071P	ICD-10 CM	AGE-RELATED OSTEOPOROSIS WITH CURRENT PATHOLOGICAL FRACTURE RIGHT ANKLE AND FOOT SUBSEQUENT ENCOUNTER FOR FRACTURE WITH MALUNION
Refracture	M84.373A	ICD-10 CM	STRESS FRACTURE UNSPECIFIED ANKLE INITIAL ENCOUNTER FOR FRACTURE
Refracture	M84.371G	ICD-10 CM	STRESS FRACTURE RIGHT ANKLE SUBSEQUENT ENCOUNTER FOR FRACTURE WITH DELAYED HEALING
Refracture	M84.572P	ICD-10 CM	PATHOLOGICAL FRACTURE IN NEOPLASTIC DISEASE LEFT ANKLE SUBSEQUENT ENCOUNTER FOR FRACTURE WITH MALUNION
Refracture	M84.571G	ICD-10 CM	PATHOLOGICAL FRACTURE IN NEOPLASTIC DISEASE RIGHT ANKLE SUBSEQUENT ENCOUNTER FOR FRACTURE WITH DELAYED HEALING
Refracture	M80.072G	ICD-10 CM	AGE-RELATED OSTEOPOROSIS WITH CURRENT PATHOLOGICAL FRACTURE LEFT ANKLE AND FOOT SUBSEQUENT ENCOUNTER FOR FRACTURE WITH DELAYED HEALING
Refracture	M84.473G	ICD-10 CM	PATHOLOGICAL FRACTURE UNSPECIFIED ANKLE SUBSEQUENT ENCOUNTER FOR FRACTURE WITH DELAYED HEALING
Refracture	M84.371S	ICD-10 CM	STRESS FRACTURE RIGHT ANKLE SEQUELA
Refracture	M84.671S	ICD-10 CM	PATHOLOGICAL FRACTURE IN OTHER DISEASE RIGHT ANKLE SEQUELA
Refracture	M84.573D	ICD-10 CM	PATHOLOGICAL FRACTURE IN NEOPLASTIC DISEASE UNSPECIFIED ANKLE SUBSEQUENT ENCOUNTER FOR FRACTURE WITH ROUTINE HEALING
Refracture	M84.472D	ICD-10 CM	PATHOLOGICAL FRACTURE LEFT ANKLE SUBSEQUENT ENCOUNTER FOR FRACTURE WITH ROUTINE HEALING
Refracture	M84.373D	ICD-10 CM	STRESS FRACTURE UNSPECIFIED ANKLE SUBSEQUENT ENCOUNTER FOR FRACTURE WITH ROUTINE HEALING
Refracture	M80.871P	ICD-10 CM	OTHER OSTEOPOROSIS WITH CURRENT PATHOLOGICAL FRACTURE RIGHT ANKLE AND FOOT SUBSEQUENT ENCOUNTER FOR FRACTURE WITH MALUNION
Refracture	M84.673K	ICD-10 CM	PATHOLOGICAL FRACTURE IN OTHER DISEASE UNSPECIFIED ANKLE SUBSEQUENT ENCOUNTER FOR FRACTURE WITH NONUNION
Refracture	M80.871K	ICD-10 CM	OTHER OSTEOPOROSIS WITH CURRENT PATHOLOGICAL FRACTURE RIGHT ANKLE AND FOOT SUBSEQUENT ENCOUNTER FOR FRACTURE WITH NONUNION
Refracture	M96.679	ICD-10 CM	FRACTURE OF TIBIA OR FIBULA FOLLOWING INSERTION OF ORTHOPEDIC IMPLANT JOINT PROSTHESIS OR BONE PLATE UNSPECIFIED LEG
Refracture	M96.671	ICD-10 CM	FRACTURE OF TIBIA OR FIBULA FOLLOWING INSERTION OF ORTHOPEDIC IMPLANT JOINT PROSTHESIS OR BONE PLATE RIGHT LEG
Refracture	M80.072S	ICD-10 CM	AGE-RELATED OSTEOPOROSIS WITH CURRENT PATHOLOGICAL FRACTURE LEFT ANKLE AND FOOT SEQUELA

Sequela	S82.64XS	ICD-10 CM	NONDISPLACED FRACTURE OF LATERAL MALLEOLUS OF RIGHT FIBULA SEQUELA
Sequela	S82.54XS	ICD-10 CM	NONDISPLACED FRACTURE OF MEDIAL MALLEOLUS OF RIGHT TIBIA SEQUELA
Sequela	S82.52XS	ICD-10 CM	DISPLACED FRACTURE OF MEDIAL MALLEOLUS OF LEFT TIBIA SEQUELA
Sequela	S82.871S	ICD-10 CM	DISPLACED PILON FRACTURE OF RIGHT TIBIA SEQUELA
Sequela	S82.845S	ICD-10 CM	NONDISPLACED BIMALLEOLAR FRACTURE OF LEFT LOWER LEG SEQUELA
Sequela	S82.301S	ICD-10 CM	UNSPECIFIED FRACTURE OF LOWER END OF RIGHT TIBIA SEQUELA
Sequela	S82.66XS	ICD-10 CM	NONDISPLACED FRACTURE OF LATERAL MALLEOLUS OF UNSPECIFIED FIBULA SEQUELA
Sequela	S82.861S	ICD-10 CM	DISPLACED MAISONNEUVE'S FRACTURE OF RIGHT LEG SEQUELA
Sequela	S82.392S	ICD-10 CM	OTHER FRACTURE OF LOWER END OF LEFT TIBIA SEQUELA
Sequela	S82.856S	ICD-10 CM	NONDISPLACED TRIMALLEOLAR FRACTURE OF UNSPECIFIED LOWER LEG SEQUELA
Sequela	S82.846S	ICD-10 CM	NONDISPLACED BIMALLEOLAR FRACTURE OF UNSPECIFIED LOWER LEG SEQUELA
Sequela	S82.865S	ICD-10 CM	NONDISPLACED MAISONNEUVE'S FRACTURE OF LEFT LEG SEQUELA
Sequela	S82.319S	ICD-10 CM	TORUS FRACTURE OF LOWER END OF UNSPECIFIED TIBIA SEQUELA
Sequela	S82.899S	ICD-10 CM	OTHER FRACTURE OF UNSPECIFIED LOWER LEG SEQUELA
Sequela	S82.891S	ICD-10 CM	OTHER FRACTURE OF RIGHT LOWER LEG SEQUELA
Sequela	S82.863S	ICD-10 CM	DISPLACED MAISONNEUVE'S FRACTURE OF UNSPECIFIED LEG SEQUELA
Sequela	S82.841S	ICD-10 CM	DISPLACED BIMALLEOLAR FRACTURE OF RIGHT LOWER LEG SEQUELA
Sequela	S82.874S	ICD-10 CM	NONDISPLACED PILON FRACTURE OF RIGHT TIBIA SEQUELA
Sequela	S82.852S	ICD-10 CM	DISPLACED TRIMALLEOLAR FRACTURE OF LEFT LOWER LEG SEQUELA
Sequela	S82.56XS	ICD-10 CM	NONDISPLACED FRACTURE OF MEDIAL MALLEOLUS OF UNSPECIFIED TIBIA SEQUELA
Sequela	S82.892S	ICD-10 CM	OTHER FRACTURE OF LEFT LOWER LEG SEQUELA
Sequela	S82.873S	ICD-10 CM	DISPLACED PILON FRACTURE OF UNSPECIFIED TIBIA SEQUELA
Sequela	S82.53XS	ICD-10 CM	DISPLACED FRACTURE OF MEDIAL MALLEOLUS OF UNSPECIFIED TIBIA SEQUELA
Sequela	S82.65XS	ICD-10 CM	NONDISPLACED FRACTURE OF LATERAL MALLEOLUS OF LEFT FIBULA SEQUELA
Sequela	S82.854S	ICD-10 CM	NONDISPLACED TRIMALLEOLAR FRACTURE OF RIGHT LOWER LEG SEQUELA
Sequela	S82.311S	ICD-10 CM	TORUS FRACTURE OF LOWER END OF RIGHT TIBIA SEQUELA
Sequela	S82.51XS	ICD-10 CM	DISPLACED FRACTURE OF MEDIAL MALLEOLUS OF RIGHT TIBIA SEQUELA
Sequela	S82.399S	ICD-10 CM	OTHER FRACTURE OF LOWER END OF UNSPECIFIED TIBIA SEQUELA
Sequela	S82.862S	ICD-10 CM	DISPLACED MAISONNEUVE'S FRACTURE OF LEFT LEG SEQUELA
Sequela	S82.842S	ICD-10 CM	DISPLACED BIMALLEOLAR FRACTURE OF LEFT LOWER LEG SEQUELA
Sequela	S82.309S	ICD-10 CM	UNSPECIFIED FRACTURE OF LOWER END OF UNSPECIFIED TIBIA SEQUELA
Sequela	S82.62XS	ICD-10 CM	DISPLACED FRACTURE OF LATERAL MALLEOLUS OF LEFT FIBULA SEQUELA
Sequela	S82.855S	ICD-10 CM	NONDISPLACED TRIMALLEOLAR FRACTURE OF LEFT LOWER LEG SEQUELA
Sequela	S82.864S	ICD-10 CM	NONDISPLACED MAISONNEUVE'S FRACTURE OF RIGHT LEG SEQUELA
Sequela	S82.853S	ICD-10 CM	DISPLACED TRIMALLEOLAR FRACTURE OF UNSPECIFIED LOWER LEG SEQUELA
Sequela	S82.876S	ICD-10 CM	NONDISPLACED PILON FRACTURE OF UNSPECIFIED TIBIA SEQUELA
Sequela	S82.312S	ICD-10 CM	TORUS FRACTURE OF LOWER END OF LEFT TIBIA SEQUELA
Sequela	S82.391S	ICD-10 CM	OTHER FRACTURE OF LOWER END OF RIGHT TIBIA SEQUELA
Sequela	S82.875S	ICD-10 CM	NONDISPLACED PILON FRACTURE OF LEFT TIBIA SEQUELA
Sequela	S82.63XS	ICD-10 CM	DISPLACED FRACTURE OF LATERAL MALLEOLUS OF UNSPECIFIED FIBULA SEQUELA
Sequela	S82.851S	ICD-10 CM	DISPLACED TRIMALLEOLAR FRACTURE OF RIGHT LOWER LEG SEQUELA
Sequela	S82.55XS	ICD-10 CM	NONDISPLACED FRACTURE OF MEDIAL MALLEOLUS OF LEFT TIBIA SEQUELA
Sequela	S82.866S	ICD-10 CM	NONDISPLACED MAISONNEUVE'S FRACTURE OF UNSPECIFIED LEG SEQUELA
Sequela	S82.61XS	ICD-10 CM	DISPLACED FRACTURE OF LATERAL MALLEOLUS OF RIGHT FIBULA SEQUELA
Sequela	S82.843S	ICD-10 CM	DISPLACED BIMALLEOLAR FRACTURE OF UNSPECIFIED LOWER LEG SEQUELA
Sequela	S82.302S	ICD-10 CM	UNSPECIFIED FRACTURE OF LOWER END OF LEFT TIBIA SEQUELA
Sequela	S82.872S	ICD-10 CM	DISPLACED PILON FRACTURE OF LEFT TIBIA SEQUELA
Sequela	S82.844S	ICD-10 CM	NONDISPLACED BIMALLEOLAR FRACTURE OF RIGHT LOWER LEG SEQUELA
Reoperation	27600	CPT	Decompression fasciotomy leg; anterior and/or lateral compartmentsonly
Reoperation	27601	CPT	Decompression fasciotomy leg; posterior compartment(s) only
Reoperation	27602	CPT	Decompression fasciotomy leg; anterior and/or lateral and posteriorcompartment(s)
Reoperation	27603	CPT	Incision and drainage leg or ankle; deep abscess or hematoma
Reoperation	27604	CPT	Incision and drainage leg or ankle; infected bursa
Reoperation	27605	CPT	Tenotomy percutaneous Achilles tendon (separate procedure); localanesthesia
Reoperation	27606	CPT	Tenotomy percutaneous Achilles tendon (separate procedure); generalanesthesia
Reoperation	27607	CPT	Incision (eg osteomyelitis or bone abscess) leg or ankle
Reoperation	27610	CPT	Arthrotomy ankle including exploration drainage or removal offoreign body
Reoperation	27612	CPT	Arthrotomy posterior capsular release ankle with or withoutAchilles tendon lengthening
Reoperation	27613	CPT	Biopsy soft tissue of leg or ankle area; superficial
Reoperation	27614	CPT	Biopsy soft tissue of leg or ankle area; deep (subfascial orintramuscular)
Reoperation	27615	CPT	Radical resection of tumor (eg sarcoma) soft tissue of leg or anklearea; less than 5 cm
Reoperation	27616	CPT	Radical resection of tumor (eg sarcoma) soft tissue of leg or anklearea; 5 cm or greater
Reoperation	27618	CPT	Excision tumor soft tissue of leg or ankle area subcutaneous; lessthan 3 cm
Reoperation	27619	CPT	Excision tumor soft tissue of leg or ankle area subfascial (eg intramuscular); less than 5 cm
Reoperation	27620	CPT	Arthrotomy ankle with joint exploration with or without biopsy with or without removal of loose or foreign body
Reoperation	27625	CPT	Arthrotomy with synovectomy ankle;
Reoperation	27626	CPT	Arthrotomy with synovectomy ankle; including tenosynovectomy
Reoperation	27630	CPT	Excision of lesion of tendon sheath or capsule (eg cyst or ganglion) leg and/or ankle

Reoperation	27632	CPT	Excision tumor soft tissue of leg or ankle area subcutaneous; 3 cmor greater
Reoperation	27634	CPT	Excision tumor soft tissue of leg or ankle area subfascial (eg intramuscular); 5 cm or greater
Reoperation	27635	CPT	Excision or curettage of bone cyst or benign tumor tibia or fibula;
Reoperation	27637	CPT	Excision or curettage of bone cyst or benign tumor tibia or fibula;with autograft (includes obtaining graft)
Reoperation	27638	CPT	Excision or curettage of bone cyst or benign tumor tibia or fibula;with allograft
Reoperation	27640	CPT	Partial excision (craterization saucerization or diaphysectomy) bone (eg osteomyelitis); tibia
Reoperation	27641	CPT	Partial excision (craterization saucerization or diaphysectomy) bone (eg osteomyelitis); fibula
Reoperation	27645	CPT	Radical resection of tumor; tibia
Reoperation	27646	CPT	Radical resection of tumor; fibula
Reoperation	27647	CPT	Radical resection of tumor; talus or calcaneus
Reoperation	27648	CPT	Injection procedure for ankle arthrography
Reoperation	27650	CPT	Repair primary open or percutaneous ruptured Achilles tendon;
Reoperation	27652	CPT	Repair primary open or percutaneous ruptured Achilles tendon; withgraft (includes obtaining graft)
Reoperation	27654	CPT	Repair secondary Achilles tendon with or without graft
Reoperation	27656	CPT	Repair fascial defect of leg
Reoperation	27658	CPT	Repair flexor tendon leg; primary without graft each tendon
Reoperation	27659	CPT	Repair flexor tendon leg; secondary with or without graft eachtendon
Reoperation	27664	CPT	Repair extensor tendon leg; primary without graft each tendon
Reoperation	27665	CPT	Repair extensor tendon leg; secondary with or without graft eachtendon
Reoperation	27675	CPT	Repair dislocating peroneal tendons; without fibular osteotomy
Reoperation	27676	CPT	Repair dislocating peroneal tendons; with fibular osteotomy
Reoperation	27680	CPT	Tenolysis flexor or extensor tendon leg and/or ankle; single eachtendon
Reoperation	27681	CPT	Tenolysis flexor or extensor tendon leg and/or ankle; multipletendons (through separate incision[s])
Reoperation	27685	CPT	Lengthening or shortening of tendon leg or ankle; single tendon(separate procedure)
Reoperation	27686	CPT	Lengthening or shortening of tendon leg or ankle; multiple tendons(through same incision) each
Reoperation	27687	CPT	Gastrocnemius recession (eg Strayer procedure)
Reoperation	27690	CPT	Transfer or transplant of single tendon (with muscle redirection orrerouting); superficial (eg anterior tibial extensors into midfoot)
Reoperation	27691	CPT	Transfer or transplant of single tendon (with muscle redirection orrerouting); deep (eg anterior tibial or posterior tibial throughinterosseous space flexor digitorum longus flexor hallucis longus
Reoperation	27692	CPT	Transfer or transplant of single tendon (with muscle redirection orrerouting); each additional tendon (List separately in addition tocode for primary procedure)
Reoperation	27695	CPT	Repair primary disrupted ligament ankle; collateral
Reoperation	27696	CPT	Repair primary disrupted ligament ankle; both collateral ligaments
Reoperation	27698	CPT	Repair secondary disrupted ligament ankle collateral (eg Watson-Jones procedure)
Reoperation	27700	CPT	Arthroplasty ankle;
Reoperation	27702	CPT	Arthroplasty ankle; with implant (total ankle)
Reoperation	27703	CPT	Arthroplasty ankle; revision total ankle
Reoperation	27704	CPT	Removal of ankle implant
Reoperation	27705	CPT	Osteotomy; tibia
Reoperation	27707	CPT	Osteotomy; fibula
Reoperation	27709	CPT	Osteotomy; tibia and fibula
Reoperation	27712	CPT	Osteotomy; multiple with realignment on intramedullary rod (eg Sofield type procedure)
Reoperation	27715	CPT	Osteoplasty tibia and fibula lengthening or shortening
Reoperation	27720	CPT	Repair of nonunion or malunion tibia; without graft (eg compressiontechnique)
Reoperation	27722	CPT	Repair of nonunion or malunion tibia; with sliding graft
Reoperation	27724	CPT	Repair of nonunion or malunion tibia; with iliac or other autograft(includes obtaining graft)
Reoperation	27725	CPT	Repair of nonunion or malunion tibia; by synostosis with fibula anymethod
Reoperation	27726	CPT	Repair of fibula nonunion and/or malunion with internal fixation
Reoperation	27727	CPT	Repair of congenital pseudarthrosis tibia
Reoperation	27730	CPT	Arrest epiphyseal (epiphysiodesis) open; distal tibia
Reoperation	27732	CPT	Arrest epiphyseal (epiphysiodesis) open; distal fibula
Reoperation	27734	CPT	Arrest epiphyseal (epiphysiodesis) open; distal tibia and fibula
Reoperation	27740	CPT	Arrest epiphyseal (epiphysiodesis) any method combined proximaland distal tibia and fibula;
Reoperation	27742	CPT	Arrest epiphyseal (epiphysiodesis) any method combined proximaland distal tibia and fibula; and distal femur
Reoperation	27745	CPT	Prophylactic treatment (nailing pinning plating or wiring) with orwithout methylmethacrylate tibia
Reoperation	27756	CPT	Percutaneous skeletal fixation of tibial shaft fracture (with orwithout fibular fracture) (eg pins or screws)
Reoperation	27758	CPT	Open treatment of tibial shaft fracture (with or without fibularfracture) with plate/screws with or without cerclage

Reoperation	27759	CPT	Treatment of tibial shaft fracture (with or without fibular fracture)by intramedullary implant with or without interlocking screws and/orcerclage
Reoperation	27766	CPT	Open treatment of medial malleolus fracture includes internalfixation when performed
Reoperation	27769	CPT	Open treatment of posterior malleolus fracture includes internalfixation when performed
Reoperation	27784	CPT	Open treatment of proximal fibula or shaft fracture includes internalfixation when performed
Reoperation	27792	CPT	Open treatment of distal fibular fracture (lateral malleolus) includes internal fixation when performed
Reoperation	27814	CPT	Open treatment of bimalleolar ankle fracture (eg lateral and medialmalleoli or lateral and posterior malleoli or medial and posteriormalleoli) includes internal fixation when performed
Reoperation	27822	CPT	Open treatment of trimalleolar ankle fracture includes internalfixation when performed medial and/or lateral malleolus; withoutfixation of posterior lip
Reoperation	27823	CPT	Open treatment of trimalleolar ankle fracture includes internalfixation when performed medial and/or lateral malleolus; withfixation of posterior lip
Reoperation	27826	CPT	Open treatment of fracture of weight bearing articular surface/portionof distal tibia (eg pilon or tibial plafond) with internal fixation when performed; of fibula only
Reoperation	27827	CPT	Open treatment of fracture of weight bearing articular surface/portionof distal tibia (eg pilon or tibial plafond) with internal fixation when performed; of tibia only
Reoperation	27828	CPT	Open treatment of fracture of weight bearing articular surface/portionof distal tibia (eg pilon or tibial plafond) with internal fixation when performed; of both tibia and fibula
Reoperation	27829	CPT	Open treatment of distal tibiofibular joint (syndesmosis) disruption includes internal fixation when performed
Reoperation	27831	CPT	Closed treatment of proximal tibiofibular joint dislocation; requiringanesthesia
Reoperation	27832	CPT	Open treatment of proximal tibiofibular joint dislocation includesinternal fixation when performed or with excision of proximal fibula
Reoperation	27842	CPT	Closed treatment of ankle dislocation; requiring anesthesia with orwithout percutaneous skeletal fixation
Reoperation	27846	CPT	Open treatment of ankle dislocation with or without percutaneousskeletal fixation; without repair or internal fixation
Reoperation	27848	CPT	Open treatment of ankle dislocation with or without percutaneousskeletal fixation; with repair or internal or external fixation
Reoperation	27860	CPT	Manipulation of ankle under general anesthesia (includes applicationof traction or other fixation apparatus)
Reoperation	27870	CPT	Arthrodesis ankle open
Reoperation	27871	CPT	Arthrodesis tibiofibular joint proximal or distal
Reoperation	27880	CPT	Amputation leg through tibia and fibula;
Reoperation	27881	CPT	Amputation leg through tibia and fibula; with immediate fittingtechnique including application of first cast
Reoperation	27882	CPT	Amputation leg through tibia and fibula; open circular (guillotine)
Reoperation	27884	CPT	Amputation leg through tibia and fibula; secondary closure or scarrevision
Reoperation	27886	CPT	Amputation leg through tibia and fibula; re-amputation
Reoperation	27888	CPT	Amputation ankle through malleoli of tibia and fibula (eg Syme Pirogoff type procedures) with plastic closure and resection of nerves
Reoperation	27889	CPT	Ankle disarticulation
Reoperation	27892	CPT	Decompression fasciotomy leg; anterior and/or lateral compartmentsonly with debridement of nonviable muscle and/or nerve
Reoperation	27893	CPT	Decompression fasciotomy leg; posterior compartment(s) only withdebridement of nonviable muscle and/or nerve
Reoperation	27894	CPT	Decompression fasciotomy leg; anterior and/or lateral and posteriorcompartment(s) with debridement of nonviable muscle and/or nerve
Reoperation	27899	CPT	Unlisted procedure leg or ankle
Reoperation	29891	CPT	Arthroscopy ankle surgical excision of osteochondral defect oftalus and/or tibia including drilling of the defect
Reoperation	29892	CPT	Arthroscopically aided repair of large osteochondritis dissecanslesion talar dome fracture or tibial plafond fracture with orwithout internal fixation (includes arthroscopy)
Reoperation	29894	CPT	Arthroscopy ankle (tibiotalar and fibulotalar joints) surgical; withremoval of loose body or foreign body
Reoperation	29895	CPT	Arthroscopy ankle (tibiotalar and fibulotalar joints) surgical;synovectomy partial
Reoperation	29897	CPT	Arthroscopy ankle (tibiotalar and fibulotalar joints) surgical;debridement limited
Reoperation	29898	CPT	Arthroscopy ankle (tibiotalar and fibulotalar joints) surgical;debridement extensive
Reoperation	29899	CPT	Arthroscopy ankle (tibiotalar and fibulotalar joints) surgical; withankle arthrodesis
